# Transcriptome Classification Reveals Molecular Subgroups in Patients with Hepatitis B Virus

**DOI:** 10.1155/2021/5543747

**Published:** 2021-03-30

**Authors:** Conghui Zhang, Jie Li, Lan Yang, Fengxia Xu, Huiyuan She, Xinghui Liu

**Affiliations:** ^1^Postgraduate Training Base in Shanghai Gongli Hospital, Ningxia Medical University, Pudong New Area, Shanghai 200135, China; ^2^Department of Clinical Laboratory, Shanghai Gongli Hospital, The Second Military Medical University, Pudong New Area, Shanghai 200135, China; ^3^Department of Infectious Medicine, Shanghai Gongli Hospital, The Second Military Medical University, Pudong New Area, Shanghai 200135, China

## Abstract

Hepatitis B virus (HBV) specifically infects hepatocytes, which can cause progressive liver fibrosis and a significantly increased risk of liver cancer. Multiple studies indicated host genetic, virological, and immunological factors could affect the HBV infection. However, the underlying mechanism involved in HBV infection remained unclear. Based on the analysis of gene expression data of 124 HBV patients (GEO accession: GSE84044), molecular subgroups of patients infected with hepatitis B virus were identified in this study, including C1, C2, and C3 groups. The age, fiber, degree of chemical and inflammation, and gene expression difference were also compared among the three sampling groups. Furthermore, the liver index was calculated using 93 liver-specific genes. The liver-specific gene expression in different molecular subgroups of HBV patients was thoroughly analyzed and then was compared with fibrosis and inflammation levels. Results showed that the C2 group was the youngest and the C3 group had the highest degree of fibrosis and inflammation. Enrichment analysis showed that metabolism-related pathways were mainly expressed in the C1 and C2 groups, and inflammation-related pathways and proteoglycans in cancer were highly expressed in the C1 and C3 groups. The liver index was higher in the C2 group than in the C1 and C3 groups, and it was the lowest in the C3 group. Macrophage M1/M2 and neutrophils were significantly different in the three groups. M1 was mainly abundant in the C3 group, and M2 and neutrophils were mainly abundant in the C2 group. This study provides novel information to understand the mechanisms of HBV infection in chronic hepatitis B (CHB) patients.

## 1. Introduction

Discovered in 1966 [1], the hepatitis B virus (HBV) infection is a public health threat worldwide [2]. Globally, 240 million people are reported to be infected with HBV [3]. HBV infection led to progressive liver fibrosis and a significantly increased risk of liver cancer. About 650,000 people die from HBV-related cirrhosis or hepatocellular carcinoma every year [4]. The situation in China is more serious, with approximately 170 million HBV-infected people [5, 6]. Therefore, in-depth exploration of pathological features and pathogenesis is of great significance for HBV control and prevention.

Nowadays, many regulators related to HBV have been studied, which are involved in the pathogenic process of HBV. Genome-wide association study (GWAS) has been used to identify genetic variants located in genes such as HLA-C [7], NOTCH4 [8], and TCF19 [9]. Moreover, the HBx protein enhanced the invasion and metastasis of liver cancer both *in vivo* and *vitro* [10], and the truncation of this protein can initiate hepatocarcinogenesis [11]. For the therapy of HBV infection, the inhibition of virus replication is one of the major approaches identified by the current researches and exhibits to reduce patient mortality and morbidity [12, 13]. Notably, toll-like receptor (TLR) ligands can be used as one of the promising antiviral drug targets for HBV infection [14]. Specifically, the pathology of HBV disease is closely associated with chronic inflammation, which is a dynamic process orchestrated by the complex interplay between virus replication and host immune response [15]. And multiple key regulators were related to modulate HBV infection and inflammation, such as IFI16, AIM2, and p46 [15–18].

Over the past decades, several antiviral drug targets for HBV infection had been discovered [19, 20]. For example, hnRNPK was identified to modulate the replicative efficiency of HBV [19]. Knockdown of hnRNPK resulted in a reduction of HBV viral load [19]. PLK1 is a key host factor for HBV replication in cells [20]. Blocking PLK1 could inhibit HBV DNA biosynthesis and strongly suppressed HBV infection in a mouse model [20]. However, current HBV treatment still cannot effectively eradicate the virus from chronic hepatitis B patients [21, 22]. HBV treatment options include only nucleoside/nucleotide analogs (NUCs) and the immunomodulatory agent interferon-alpha (IFN-*α*) [23, 24]. Moreover, the risk of HBV reactivation rises when patients receive immunosuppressive or antitumor therapy [25]. Therefore, the identification of drug targets and underlying mechanism for HBV-infected patients is urgently needed. In this study, we collected transcriptome data from public database and conducted a systematic data analysis, aiming to identify genes involved in HBV infection and uncover the underlying mechanism.

## 2. Materials and Methods

### 2.1. Collection of Gene Expression Data of HBV Patients

The gene expression data was obtained from Gene Expression Omnibus (GEO) with accession GSE84044 [26], which included 124 chronic hepatitis B (CHB) patients. The clinical characteristics are summarized in Table 1. The count-based gene expression matrix was used for the analysis in this study.

### 2.2. Consensus Clustering Algorithm

The consensus clustering of samples from the GSE84044 dataset was conducted by the ConsensusClusterPlus R package [27]. The number of clusters was determined by the cumulative distribution function (CDF) and consistency score (greater than 0.8 in all clusters).

### 2.3. Liver Index Calculation

We performed the single-sample gene set enrichment analysis (ssGSEA) using the R gsva package [28] to calculate the liver index using 93 liver-specific genes, which represent the normal liver metabolism capability from previous study [29].

### 2.4. Differential Gene Expression Analysis

The differential expression analysis was conducted by R DESeq2 package [30]. The genes with adjusted *p* value < 0.05 and log_2_ fold change > 1 were considered as differentially expressed.

### 2.5. Evaluation of Tumor-Infiltrating Immune Cells

The proportions of 22 tumor-infiltrating lymphocyte subsets in the liver tissues were calculated by CIBERSORT [31]. Besides, *p* < 0.05 was regarded as an accurate immune cell fraction, and the cell proportions between the groups were compared by the Wilcoxon-rank sum test.

### 2.6. Functional Enrichment Analysis

Gene ontology (GO) and Kyoto Encyclopedia of Genes and Genomes (KEGG) pathways were performed for each selected module by overrepresentation enrichment analysis using R clusterProfiler package [32]. Items with adjusted *p* < 0.05 were regarded to be significant. The enrichment analysis of liver-specific genes was conducted by gene set enrichment analysis. The genes were ranked by the statistics calculated by R DESeq2 [30] package.

### 2.7. Statistical Analysis

Student's *t*-test was used to compare gene expression differences between tumor and normal tissues. All the statistics were done using the R software (version 4.0.2). *p* value < 0.05 was set as statistically significant for all the analyses.

## 3. Results

### 3.1. Identification of Molecular Subgroups in Patients with Hepatitis B Virus

**Figure 1:**
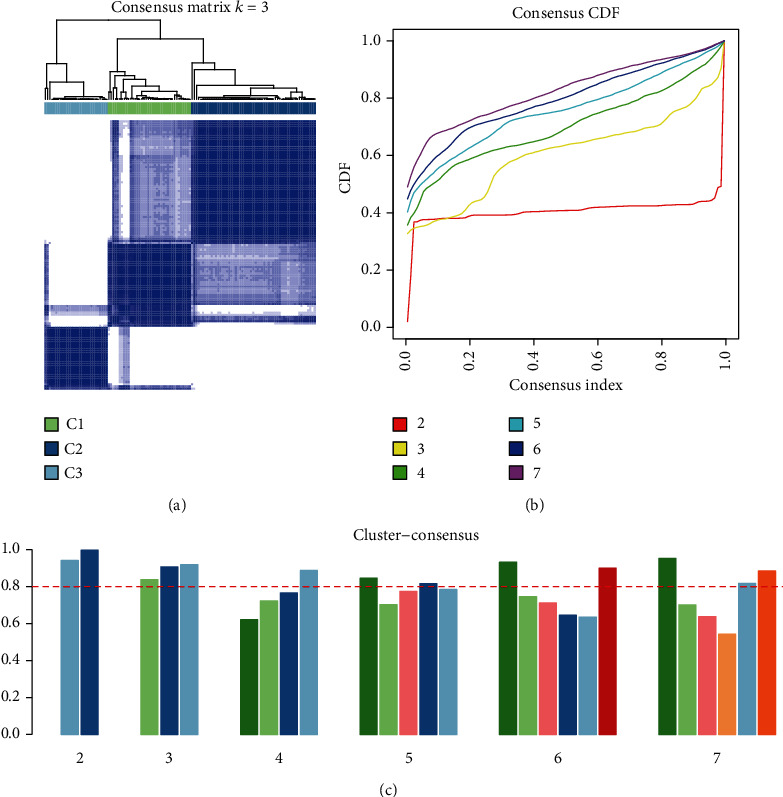
Identification of molecular subgroups in patients with hepatitis B virus. (a) 124 HBV patients were used to divide all samples into three categories, namely, C1, C2, and C3, with 38, 57, and 29 samples, respectively. (b) The CDF analysis of the three categories. (c) The consistency score of each cluster was determined.

Based on the gene expression data of 124 HBV patients (GSE84044), the consensus clustering algorithm was used to divide all samples into three categories, namely, C1, C2, and C3, with 38, 57, and 29 samples, respectively (Figure 1(a)). Samples were divided into three categories based on the cumulative distribution function (CDF) and consistency score. The CDF analysis showed that in the three categories, the area under the CDF curve did not increase significantly (Figure 1(b)); at the same time, the consistency score must be greater than 80% in each cluster (Figure 1(c)). Ultimately, three categories were selected for downstream analysis.

### 3.2. The Three Subgroups Have Significant Differences in Age, Fibrosis, and Inflammatory Levels

**Figure 2:**
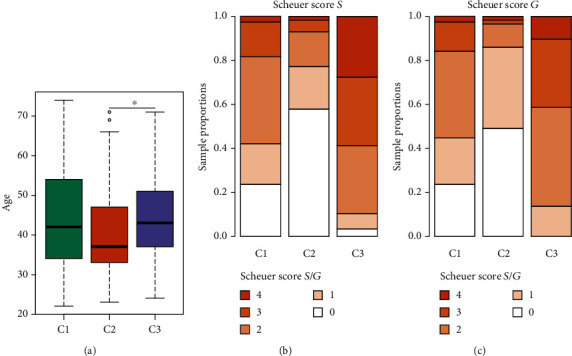
Significant differences in age, fibrosis, and inflammatory levels in three subgroups. (a) The distribution of age in C1, C2, and C3 groups was shown. (b, c) The proportions of fibrosis in C1, C2, and C3 groups were significantly different.

To further explore the clinical significance of the sample classification, the age, fibrosis, and inflammation of the three groups of samples were compared. Specifically, the age of the C2 group was younger than that of the C1 and C3 groups, but no difference was observed between the C1 and C3 groups (Figure 2(a)). The degree of fibrosis and verification was divided into 5 levels, from 0 to 4 points. The higher the score, the more serious the fibrosis. The proportions of the above five levels in the C1, C2, and C3 groups were significantly different (Figures 2(b) and 2(c)). Specifically, the degree of fibrosis and inflammation of the C2 group was lighter than that of the C1 and C3 groups, of which the C3 group was the most severe, followed by the C1 group. These results indicated that C2 might have a higher grade in the pathogenetic process of HBV infection.

### 3.3. Molecular Characterization of the Molecular Subgroups

**Figure 3:**
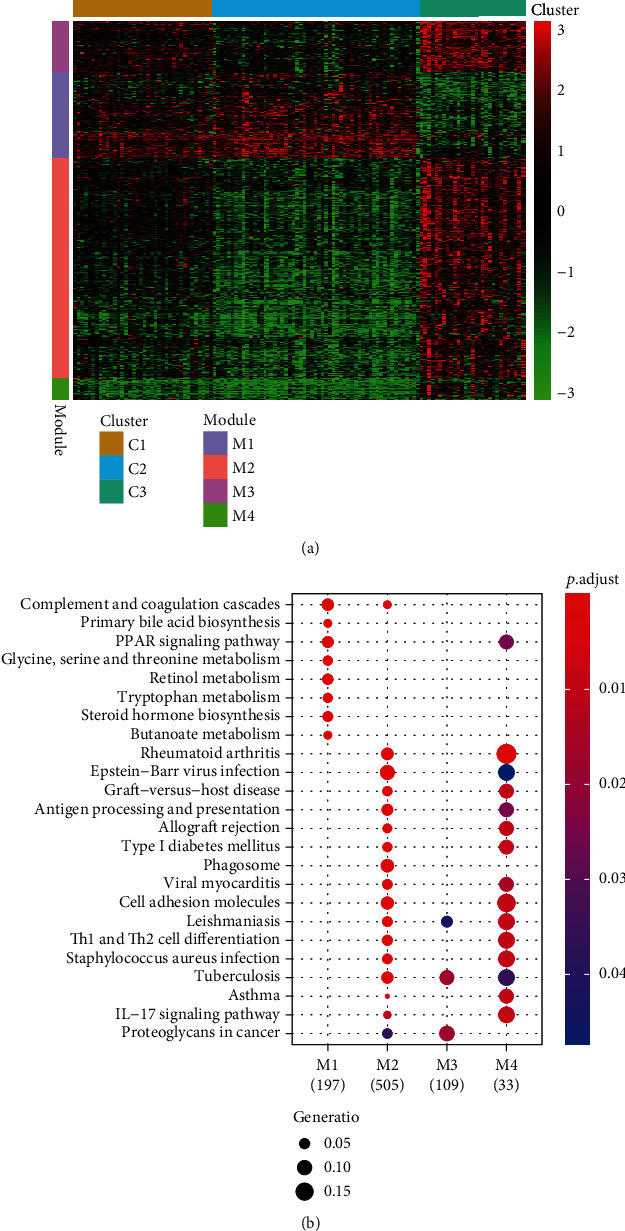
Molecular characterization of the molecular subgroups. (a) Unsupervised clustering of the above 2006 genes can classify these genes into four modules, named M1–M4. (b) Bioinformatics analysis of differently expressed genes in M1–M4 is shown.

To further explore the differences in the molecular level of each group, the DESeq2 package was employed, and three groups were compared with each other. A total of 2006 differential genes were screened out (FDR < 0.1 and log_2_ fold change > 0.5, Supplementary Table S1). Unsupervised clustering of the above 2006 genes can classify these genes into four modules, named M1–M4 (Figure 3(a), Supplementary Table S2). Through gene set enrichment analysis, it was found that M1 was mainly enriched by metabolic pathways and PPAR signaling, which were mainly expressed in the C1 and C2 groups; M2 and M4 were mainly enriched by the inflammation-related pathways, and the specific pathway of M3 was proteoglycans in cancer, and M2, M3, and M4 were mainly expressed in the C1 and C3 groups. The high expression of the two groups (Figure 3(b)) indicated that these two groups of patients had a higher tendency to precancerous lesions.

### 3.4. Subgroup C2 Preserves a Higher Liver Functionality Than C1 and C3

**Figure 4:**
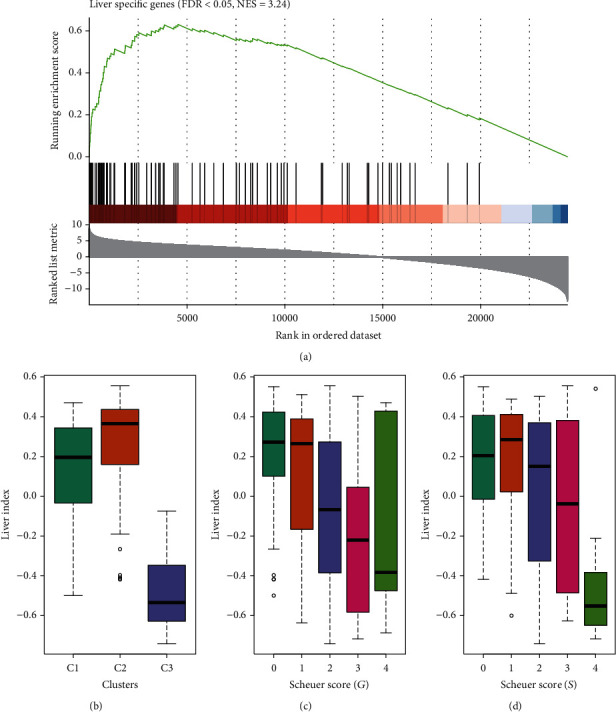
Subgroup C2 preserves a higher liver functionality than C1 and C3. (a) The liver-specifically expressed genes were highly enriched in C2 vs. C1 and C3 highly expressed gene. (b) Liver index was significantly higher in the C2 group than in the C1 and C3 groups, and the liver index of the C3 group was the lowest. Liver index was also highly negatively correlated with (c) fibrosis and (d) inflammation levels.

Since module M1 is mainly a metabolic pathway and is highly expressed in the C2 group, we compared C2 and C1 with C3 and found that the liver-specifically expressed genes [29] were highly enriched in C2 vs. C1 and C3 highly expressed gene (Figure 4(a), FDR < 0.05). Combining the single-sample gene set enrichment analysis (ssGSEA) method and 93 liver-specifically expressed genes, a liver index (liver index) was constructed. Results showed that the liver index was significantly higher in the C2 group than in the C1 and C3 groups, and the liver index of the C3 group was the lowest (Figure 4(b)). At the same time, the liver index was also highly negatively correlated with fibrosis and inflammation levels (Figures 4(c) and 4(d)).

### 3.5. Differential Abundances of Immune Cells in the Molecular Subgroups

**Figure 5:**
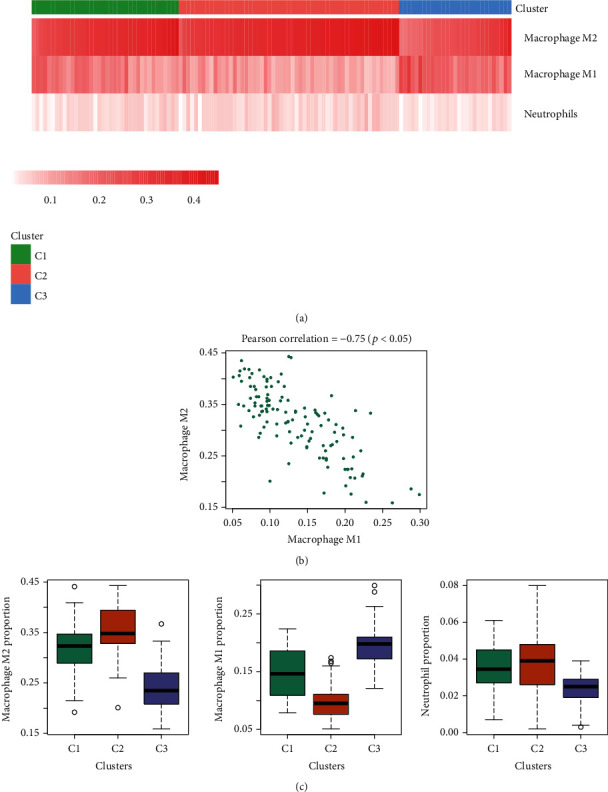
Differential abundances of immune cells in the molecular subgroups. (a) The macrophage M1/M2 and neutrophils showed significant difference between the three groups. (b) M1 and M2 were highly negatively correlated. (c) The levels of macrophage M2, macrophage M1, and neutrophils in C1, C2, and C3 groups are shown.

As the C3 group has a higher level of inflammation, the CIBERSORT was used to calculate the relative proportion of immune cells in each sample. Specifically, the macrophage M1/M2 and neutrophils showed significant difference between the three groups (Figure 5(a)). It is worth noting that M1 and M2 were highly negatively correlated (Figure 5(b)), indicating that these two cell types may be mutually exclusive. Specifically, M1 was mainly abundant in the C3 group, and M2 and neutrophils were mainly abundant in the C2 group (Figure 5(c)).

## 4. Discussion

More than 300 million people worldwide are infected with HBV, with a higher infection rate in developing countries [33–35]. In particular, the incidence of HBV infection exceeds 8 percent in most Asian regions [33]. Besides, three-quarters of persons infected with HBV do not even know they are infected [33]. There was a significant difference in genotypes of infants between the HBV-infected pregnant women and those without HBV infection [36]. This causal relationship may be HBV-driven [36]. Meanwhile, HBV is regarded as a human oncogenic virus, but the molecular mechanism of its tumorigenesis is unclear [37]. In this study, bioinformatics methods were used to analyze the gene expression data of 124 HBV patients to further explore the different molecular subgroups of HBV patients, including age, fibrosis, inflammation degree, and related pathways. Furthermore, the liver index was calculated using 93 liver-specific genes. The liver-specific gene expression in different molecular subgroups of HBV patients was thoroughly analyzed and then compared with fibrosis and inflammation levels.

From the gene expression data of 124 HBV patients, three types of submolecules were identified, namely, C1, C2, and C3. Results showed that the C2 group was the youngest and the C3 group had the most severe liver fibrosis and inflammation. The C1 and C2 groups were closely related to metabolic-related pathways, while inflammation and cancer-related pathways were closely related to the C1 and C3 groups. Many studies have shown that some pathways in HBV patients are different from those in normal people, which is also a starting point for HBV treatment. For example, the HBV-STAT3-miR-328-3p-FOXO4 axis participates in the chronic HBV infection pathogenesis [38]. HBV impairs IFN activity by hijacking the IFN/JAK/STAT pathway through HBeAg [39]. The liver index of the C2 group was higher than that of the other two groups, and the liver index of the C3 group was the lowest. At the same time, the liver index was highly negatively correlated with fibrosis and inflammation, which was consistent with the enrichment pathway analysis.

Of note, we identified 2006 differentially expressed genes among the three types of HBV patients. Using unsupervised clustering analysis, we revealed 2006 genes could be classified into four modules, named M1–M4. Bioinformatics analysis indicated these modules were related to regulate multiple crucial pathways in HBV infection and liver tumorigenesis. For example, M1 was enriched in metabolic pathways and PPAR signaling. Peroxisome proliferator-activated receptor-gamma coactivator 1 alpha (PGC-1alpha), a major metabolic regulator, was identified to strongly coactivate HBV transcription [40, 41]. M3 was especially involved in regulating proteoglycans in cancer. Proteoglycans are characterized by a central protein backbone that is decorated with linear sulfated glycosaminoglycan side chains. Proteoglycans were related to regulate the biochemical and mechanical properties of the interstitial extracellular matrix. Various studies demonstrated proteoglycans enhanced malignant transformation and alter antitumor therapy response [42]. M2 and M4 were mainly enriched by the inflammation-related pathways, such as IL-17 signaling and Th1 and Th2 cell differentiation, which was involved in modulating both HBV infection and liver cancer progression. For example, IL-17 activates the IL-6/STAT3 signal pathway in the proliferation of hepatitis B virus-related hepatocellular carcinoma [43]. Hepatitis B virus induces IL-23 production in antigen-presenting cells and causes liver damage via the IL-23/IL-17 axis [44].

Several limitations should also be noted in this study. Firstly, the conclusion of this study was obtained using bioinformatics analysis and lacking of experimental validation. We will validate the clinical significance of the sample classification by collecting clinical samples. Secondly, several hub signaling pathways were revealed to modulate HBV infection, such as PPAR signaling, and the inflammation-related pathways. Exploring the effects of this signaling on HBV infection with pathway-specific inhibitors could further strengthen the findings of this study. In summary, our study for the first time comprehensively demonstrated the potential mechanisms of HBV promoting liver fibrosis and tumor progression.

## 5. Conclusions

In conclusion, using bioinformatics to complete and analyze the gene expression data of HBV patients, a lot of useful information is obtained, which provides a reference for further understanding of the pathogenic mechanism of HBV and has predictive value.

## Figures and Tables

**Table 1:** The summarized clinical characteristics of the 124 CHB patients.

Clinical factor	# of samples (*n* = 124)	Ratio
*Gender*		
Female	36	29.03%
Male	88	70.97%
*Age*		
<50 years	92	74.19%
>50 years	32	25.81%
*Scheuer score grading*		
0	37	29.84%
1	33	26.61%
2	34	27.42%
3	15	12.10%
4	5	4.03%
*Scheuer score staging*		
0	43	34.68%
1	20	16.13%
2	33	26.61%
3	18	14.52%
4	10	8.06%

## Data Availability

Previously reported gene expression and clinical data were used to support this study and are available at Gene Expression Omnibus (GEO, https://www.ncbi.nlm.nih.gov/gds). These prior studies (and datasets) are cited at relevant places within the text as references.

## Supplementary Materials

Supplementary 1 Supplementary Table S1: the subgroup-specific genes in the three subgroups of CHB patients.

Supplementary 2 Supplementary Table S2: the functional module-specific genes in CHB patients.
