# Supplementary material for: Transcriptome Classification Reveals Molecular Subgroups in Patients with Hepatitis B Virus
Source: Comput Math Methods Med. 2021 Mar 30;2021:5543747. doi: 10.1155/2021/5543747 (PMC8028738; doi:10.1155/2021/5543747)
Supplement: Supplementary 1 — Supplementary Table S1: the subgroup-specific genes in the three subgroups of CHB patients. [file 5543747.f1.pdf]

Table S1. The differentially expressed genes in the subgroups

gene	FDR (C1 vs. C2)	FDR (C1 vs. C3)	FDR (C2 vs. C3)	log2-FoldChange (C1 vs. C2)	log2-FoldChange (C1 vs. C3)	log2-FoldChange (C2 vs. C3)
A1CF	0.039514	9.93E-09	6.34E-09	-0.07873	0.554932	0.633657
AACS	5.23E-05	5.73E-05	1.87E-09	0.454532	-0.45063	-0.90516
AADACP1	0.109525	0.003681	1.33E-05	-0.24134	0.454731	0.69607
AADAT	0.000422	3.24E-09	3.59E-11	-0.23948	0.54361	0.783087
AAED1	0.000107	1.56E-05	2.08E-10	0.239444	-0.28576	-0.5252
AASS	0.118714	7.10E-06	2.85E-07	-0.11915	0.61097	0.730116
ABCA6	0.081872	2.39E-09	3.11E-09	-0.07691	0.544622	0.621536
ABCB1	0.000481	9.05E-08	1.57E-11	0.319941	-0.51188	-0.83182
ABCB4	0.025223	0.00746	6.44E-06	0.235767	-0.33733	-0.5731
ABCC1	1.94E-06	1.62E-07	9.12E-11	0.297857	-0.38921	-0.68707
ABCG2	0.020901	1.08E-07	7.37E-10	-0.1859	0.7734	0.959298
ABHD15	0.385513	3.14E-07	1.29E-08	-0.04885	0.466745	0.515592
abParts	1.79E-08	9.69E-05	5.17E-11	0.929474	-0.96489	-1.89437
ABRACL	0.029467	0.008079	3.90E-05	0.266086	-0.30934	-0.57543
AC004538.3	0.92733	1.37E-07	1.38E-07	0.015908	0.633685	0.617776
AC012065.7	0.149521	2.31E-06	6.50E-07	-0.10922	0.481611	0.590827
AC017002.2	0.022274	0.000223	5.44E-08	0.201984	-0.53709	-0.73908
AC079767.4	0.001294	0.001043	5.97E-08	0.28002	-0.53505	-0.81507
AC128677.4	6.03E-07	0.000575	5.45E-10	0.599582	-0.64625	-1.24583
ACACB	0.050444	1.08E-08	4.03E-09	-0.13212	0.446159	0.578279
ACADL	0.173653	3.63E-09	1.51E-09	-0.07332	0.520692	0.594012
ACADS	0.007314	9.55E-12	1.57E-11	-0.16408	0.589308	0.753384
ACADSB	0.00112	7.37E-10	8.29E-11	-0.21801	0.730699	0.948712
ACE2	0.094243	0.000635	1.02E-06	0.244373	-0.89305	-1.13743
ACLY	0.032081	2.35E-08	1.43E-09	0.156517	-0.48452	-0.64104
ACMSD	0.003147	8.10E-06	1.72E-10	-0.28644	0.458485	0.744921
ACOT13	0.000164	1.48E-07	9.01E-10	-0.16278	0.358757	0.521533
ACOT9	7.74E-07	1.98E-12	1.57E-11	0.221838	-0.44497	-0.66681
ACOX2	0.000742	3.17E-10	2.49E-11	-0.15928	0.471716	0.630997
ACP5	0.009513	0.000667	3.35E-08	0.316956	-0.36536	-0.68231
ACSL4	1.68E-06	2.46E-07	4.42E-10	0.365945	-1.02593	-1.39187
ACSM2A	0.046903	2.03E-05	1.06E-07	-0.12566	0.470927	0.596591
ACSM2B	0.034876	5.39E-06	1.11E-09	-0.16966	0.474304	0.643961
ACSM3	0.022764	3.42E-11	7.58E-11	-0.17781	0.843738	1.021547
ACSM5	0.313409	3.12E-05	3.11E-07	-0.10455	0.44798	0.552526
ACTB	0.002955	2.65E-09	3.23E-10	0.212867	-0.34634	-0.55921
ACTG1	0.009161	1.61E-08	1.29E-09	0.217554	-0.37757	-0.59513
ACYP1	2.88E-05	0.001737	1.23E-09	0.317398	-0.24325	-0.56065
ADA	3.48E-06	8.73E-07	5.62E-11	0.364471	-0.54631	-0.91078
ADAMDEC1	0.000797	6.09E-05	4.91E-09	0.505588	-1.28037	-1.78596
ADAMTS	1.33E-07	6.10E-10	1.57E-11	0.345108	-0.5476	-0.89271
ADAMTSL2	0.003354	0.028974	5.89E-05	0.276379	-0.32946	-0.60584
ADAP2	1.20E-06	0.003236	3.63E-09	0.318618	-0.25159	-0.57021
ADCY1	0.050444	7.36E-09	1.79E-10	-0.12765	0.50463	0.63228
ADCY10	0.002153	1.66E-05	2.42E-09	-0.23518	0.350973	0.586154
ADCY7	4.36E-08	9.27E-06	2.43E-11	0.337494	-0.25301	-0.5905

ADH6	0.518097	6.70E-10	2.92E-10	-0.03377	0.5666	0.60037
ADHFE1	0.042642	2.00E-06	1.87E-09	-0.14813	0.477632	0.625765
ADIRF	0.883163	0.014722	0.005239	0.081742	-0.50479	-0.58653
ADORA2A-AS1	0.009684	0.000128	2.14E-08	-0.21634	0.33944	0.555785
ADORA3	0.357219	0.000171	5.47E-06	0.124399	-0.52969	-0.65408
ADRA1A	0.757034	1.60E-06	3.11E-07	-0.01894	0.539521	0.558458
ADRA2A	8.40E-06	0.004183	2.18E-09	0.421938	-0.53303	-0.95497
ADRBK2	6.94E-08	9.05E-08	1.91E-11	0.338651	-0.35523	-0.69388
AEBP1	8.67E-08	7.02E-08	1.57E-11	0.684969	-1.11383	-1.7988
AF070581	0.130247	3.39E-07	4.23E-09	0.087505	-0.57975	-0.66725
AGA	1.80E-06	2.15E-08	2.58E-11	0.30088	-0.37993	-0.68081
AGBL2	0.460806	7.59E-08	1.43E-08	-0.11048	0.885321	0.995798
AGL	0.205118	4.02E-09	1.87E-07	-0.00499	0.502674	0.507663
AGXT2	0.169737	4.49E-05	1.02E-07	-0.10384	0.457516	0.561358
AIF1	0.00047	0.000161	3.87E-08	0.20164	-0.32685	-0.52849
AIM2	0.000383	0.001386	2.75E-08	0.565048	-0.74199	-1.30704
AJUBA	5.37E-05	0.000342	2.54E-09	0.444096	-0.37766	-0.82175
AKIP1	4.44E-06	3.14E-07	2.38E-11	0.232928	-0.28915	-0.52208
AKR1B1	2.73E-08	1.48E-08	3.48E-11	0.438423	-0.52032	-0.95874
AKR1B10	0.202778	1.48E-06	6.34E-09	0.573202	-2.95599	-3.52919
AKR1D1	0.229723	1.93E-07	2.07E-09	-0.08066	0.574537	0.655197
ALAD	0.202778	4.45E-10	4.91E-10	-0.05803	0.466833	0.524863
ALDH1L1	0.07627	3.93E-10	7.58E-11	-0.09976	0.550803	0.65056
ALDH6A1	0.569099	1.61E-08	3.68E-08	-0.01385	0.520581	0.534433
ALDOA	6.84E-05	1.08E-08	1.06E-10	0.247262	-0.48971	-0.73697
ALOX5	5.98E-08	7.74E-05	3.59E-11	0.290754	-0.22902	-0.51977
ALOX5AP	1.68E-08	7.59E-08	3.36E-11	0.494243	-0.6595	-1.15375
ALPK2	0.392802	0.086372	0.001462	-0.27574	0.620526	0.896262
AMICA1	6.77E-09	1.98E-10	1.57E-11	0.521011	-0.65021	-1.17122
AMIGO2	5.53E-05	0.000223	1.29E-09	0.416289	-0.306	-0.72229
AMN	0.071056	9.89E-06	5.73E-09	-0.18591	0.494626	0.680532
AMT	0.004939	9.93E-09	1.41E-10	-0.12686	0.412992	0.539854
ANGPTL2	0.000117	3.39E-07	4.72E-11	0.19304	-0.42923	-0.62227
ANKRD22	3.93E-05	0.000109	1.34E-10	0.605565	-0.83434	-1.4399
ANKRD29	0.000838	1.18E-06	5.92E-11	0.407676	-0.82416	-1.23183
ANKRD35	0.066653	2.03E-05	1.17E-07	-0.13612	0.601444	0.737561
ANLN	0.000138	1.29E-05	2.31E-10	0.340749	-0.75703	-1.09778
ANXA1	1.06E-07	1.75E-09	1.57E-11	0.307302	-0.37121	-0.67851
ANXA10	0.003786	5.86E-08	2.43E-10	-0.25055	0.834478	1.085023
ANXA13	0.09815	5.73E-05	1.28E-07	0.328071	-0.84742	-1.17549
ANXA2	2.94E-06	3.03E-12	1.57E-11	0.368417	-0.73363	-1.10205
ANXA2P2	4.18E-05	3.03E-12	1.57E-11	0.426206	-1.00028	-1.42649
ANXA3	0.000965	1.29E-05	4.42E-10	0.513924	-0.90798	-1.4219
ANXA4	2.65E-05	7.59E-08	1.69E-11	0.30297	-0.54359	-0.84656
ANXA5	1.17E-05	9.55E-12	2.38E-11	0.364134	-0.72152	-1.08565
AOAH	5.66E-06	0.000144	2.19E-10	0.364667	-0.37017	-0.73484
AOX1	0.092936	1.26E-09	9.01E-10	-0.07345	0.588884	0.662336
AP000253.1	0.906416	4.16E-08	2.36E-07	0.039451	0.834421	0.79497
AP1S2	1.80E-06	1.18E-06	4.10E-11	0.269066	-0.35531	-0.62437
APCDD1	0.005249	0.002595	3.68E-08	0.255183	-0.42445	-0.67964
APH1B	3.71E-05	2.18E-09	2.49E-11	0.172226	-0.35991	-0.53214
APOA5	0.074119	0.000263	2.59E-07	-0.23375	0.66176	0.89551
APOBEC3A	0.024383	0.044945	6.33E-05	0.24175	-0.30531	-0.54706
APOBEC3A_B	0.024383	0.044945	6.33E-05	0.24175	-0.30531	-0.54706

APOBEC3B	4.06E-05	9.27E-06	1.06E-10	0.538528	-0.98604	-1.52457
APOBEC3G	4.18E-05	4.33E-11	1.69E-11	0.229582	-0.56801	-0.7976
APOF	0.044713	4.52E-09	8.59E-10	-0.11632	0.830736	0.947058
APOL3	2.55E-06	2.00E-06	5.62E-11	0.654135	-0.92034	-1.57447
AQP1	2.31E-06	0.000103	7.37E-10	0.889771	-0.93995	-1.82973
AR	0.054414	2.16E-11	3.40E-10	-0.06624	0.694839	0.761079
ARF4	0.177862	3.78E-08	3.03E-08	0.099564	-0.43127	-0.53084
ARHGAP10	0.175754	9.47E-07	5.44E-08	-0.12207	0.509201	0.631267
ARHGAP18	6.90E-07	1.61E-08	2.49E-11	0.360889	-0.46352	-0.82441
ARHGAP25	1.56E-07	6.20E-06	4.51E-11	0.253125	-0.29422	-0.54734
ARHGAP4	8.67E-06	0.000171	4.42E-10	0.235842	-0.26499	-0.50084
ARHGAP9	2.41E-08	9.89E-06	2.43E-11	0.52114	-0.5322	-1.05334
ARHGDIB	6.06E-05	1.48E-07	1.79E-10	0.201527	-0.38184	-0.58337
ARHGEF18	2.80E-05	7.48E-07	3.07E-10	0.237872	-0.28	-0.51787
ARHGEF26	0.811944	9.69E-05	0.000275	0.085473	0.56172	0.476247
ARHGEF3	3.65E-09	8.21E-11	1.57E-11	0.574333	-0.67842	-1.25275
ARHGEF6	1.55E-06	1.48E-06	1.01E-10	0.297831	-0.31639	-0.61422
ARL4C	3.44E-07	1.37E-07	1.57E-11	0.32718	-0.39011	-0.71729
ARL6IP5	0.008665	3.53E-06	3.28E-09	0.17407	-0.33509	-0.50916
ARMCX1	2.08E-06	6.45E-05	3.73E-11	0.467948	-0.39239	-0.86034
ARMCX2	2.51E-07	4.06E-06	2.49E-11	0.454566	-0.47303	-0.9276
ARMCX3	0.015866	9.69E-05	1.62E-07	0.241367	-0.36963	-0.611
ARNTL	0.666416	0.003236	0.010167	0.103147	0.592626	0.489478
ARNTL2	0.042642	6.10E-10	9.63E-11	0.113077	-0.49815	-0.61123
ARPC1B	1.11E-06	1.36E-11	1.57E-11	0.415549	-0.64886	-1.06441
ARPC2	1.13E-05	2.41E-12	1.57E-11	0.185861	-0.42044	-0.6063
ARPC3	0.000493	1.12E-09	4.10E-11	0.188038	-0.41117	-0.59921
ARRDC2	0.003636	1.38E-06	9.52E-10	0.165896	-0.4452	-0.6111
ARRDC3	0.477079	1.90E-05	3.02E-06	-0.11477	0.549583	0.66435
ARRDC4	0.224626	0.044945	0.000479	0.231654	-0.27725	-0.5089
ARSE	0.292008	2.93E-05	9.75E-07	-0.06767	0.485637	0.553309
ARSJ	0.012307	0.000223	6.02E-09	0.182247	-0.42175	-0.604
ASB9	0.144149	0.005168	6.99E-06	-0.27918	0.657342	0.936521
ASCC3	2.08E-06	4.77E-05	1.88E-10	0.347064	-0.32208	-0.66914
ASNS	9.49E-05	9.47E-07	2.87E-11	0.203107	-0.36177	-0.56488
ASPA	0.090373	1.29E-05	2.04E-08	-0.19579	0.54347	0.739261
ASPG	0.019115	4.06E-06	3.28E-09	-0.27374	0.479708	0.753447
ASPHD2	7.51E-05	3.79E-06	1.88E-10	0.254167	-0.43382	-0.68799
ASPM	0.000533	0.000211	5.43E-09	0.277015	-0.53772	-0.81473
ASPN	0.000151	0.00159	6.26E-08	0.383482	-0.35241	-0.7359
ASRGL1	0.153348	5.97E-07	1.87E-09	0.119156	-0.41777	-0.53692
ATAD2	0.006001	0.000223	3.03E-08	0.225834	-0.35054	-0.57637
ATF5	0.012087	7.48E-07	3.35E-08	-0.15948	0.362595	0.522071
ATF7IP2	0.091674	1.75E-09	9.12E-11	-0.11874	0.459709	0.578451
ATP10D	1.21E-05	0.055229	2.61E-08	0.50504	-0.23281	-0.73785
ATP6V0B	0.001882	1.78E-08	1.88E-10	0.161334	-0.34873	-0.51006
ATP6V1F	0.000383	4.31E-07	7.00E-10	0.149779	-0.3615	-0.51128
ATP8B2	0.007896	1.47E-05	1.51E-09	0.194801	-0.31918	-0.51398
ATP8B4	6.71E-07	0.001322	8.13E-10	0.464459	-0.295	-0.75946
AURKA	0.145789	1.66E-05	1.78E-07	0.103488	-0.4903	-0.59379
AVPR1A	0.04831	1.86E-06	8.13E-10	-0.26129	0.660542	0.921834

AX747132	0.053563	0.001737	1.50E-05	-0.21018	0.42839	0.638568
AX747135	0.000547	2.87E-06	2.08E-10	-0.18882	0.329118	0.517936
AZGP1	0.00101	1.75E-09	5.62E-11	-0.18537	0.467848	0.653214
B3GAT1	0.157227	2.75E-05	3.52E-08	-0.19203	0.748515	0.940542
BAAT	0.034291	9.89E-08	4.23E-09	-0.15501	0.510721	0.66573
BACE2	3.61E-09	3.93E-10	1.57E-11	0.483326	-0.6857	-1.16903
BAG3	0.006907	7.28E-05	7.70E-10	0.250181	-0.33673	-0.58691
BAMBI	0.022024	3.99E-07	1.76E-08	0.216391	-0.65791	-0.8743
BARD1	3.60E-05	2.26E-10	2.04E-11	0.253006	-0.55579	-0.8088
BATF	0.000559	6.09E-05	1.43E-09	0.241679	-0.34531	-0.58699
BATF2	2.65E-05	0.002175	1.29E-09	0.30161	-0.33089	-0.6325
BAX	6.64E-05	0.001264	1.58E-08	0.36497	-0.32994	-0.69491
BAZ1A	1.95E-05	0.004011	4.71E-08	0.355456	-0.24572	-0.60118
BBOX1	0.018194	4.31E-07	2.42E-09	-0.20609	1.054804	1.260898
BBS12	0.000493	0.000177	4.91E-09	0.304732	-0.38459	-0.68932
BBS7	3.07E-05	0.00052	1.29E-09	0.251651	-0.29661	-0.54826
BC034319	0.003419	7.59E-08	1.99E-10	0.285724	-0.82921	-1.11493
BC038731	0.985643	1.02E-06	4.75E-07	0.001177	0.546003	0.544826
BC040327	0.92733	1.37E-07	1.38E-07	0.015908	0.633685	0.617776
BCAT1	0.00032	9.93E-09	1.60E-11	0.313185	-0.63244	-0.94563
BCHE	0.441277	1.08E-08	3.11E-09	-0.07605	0.952276	1.028323
BCL11B	1.51E-05	1.86E-06	1.72E-11	0.361539	-0.44108	-0.80261
BCL2A1	7.73E-09	4.37E-06	1.85E-11	0.763323	-0.81749	-1.58082
BEX1	0.031573	0.001517	1.02E-06	0.235548	-0.47896	-0.7145
BEX2	0.001239	2.27E-07	2.56E-10	0.322954	-0.98776	-1.31071
BEX4	7.05E-05	2.00E-06	5.62E-11	0.467371	-0.51309	-0.98046
BEX5	0.184388	0.0007	4.44E-06	0.130975	-0.43661	-0.56758
BICC1	1.55E-06	1.18E-06	3.73E-11	0.614099	-0.94917	-1.56327
BIN2	5.57E-08	4.70E-06	7.58E-11	0.386356	-0.41161	-0.79796
BIRC3	4.33E-09	2.00E-06	1.57E-11	0.615302	-0.63005	-1.24536
BLM	0.292008	1.33E-08	1.36E-08	0.055505	-0.4718	-0.52731
BLOC1S2	0.000159	1.37E-07	7.95E-11	0.209644	-0.32994	-0.53958
BLVRA	9.91E-06	1.75E-09	2.78E-11	0.220619	-0.45677	-0.67739
BMS1P20	0.138825	5.47E-10	1.06E-09	0.057548	-0.4463	-0.50384
BORA	0.000193	6.97E-07	5.72E-10	0.218181	-0.40349	-0.62167
BTG2	5.06E-05	2.59E-08	1.91E-11	0.192916	-0.33326	-0.52617
BTK	2.82E-07	0.004364	7.28E-11	0.431134	-0.26585	-0.69699
BTLA	0.00032	1.10E-06	1.27E-10	0.210433	-0.49799	-0.70842
BTN3A2	6.10E-06	0.002175	6.34E-10	0.466736	-0.37806	-0.8448
BTN3A3	9.91E-06	8.67E-05	7.58E-11	0.47623	-0.48458	-0.96081
BUB1B	0.000173	2.68E-06	6.59E-11	0.555754	-1.03955	-1.5953
C10orf54	7.02E-06	1.01E-09	1.72E-11	0.276042	-0.47932	-0.75536
C11orf71	0.173653	2.15E-08	3.45E-09	-0.09209	0.450413	0.5425
C11orf80	9.77E-05	1.48E-06	1.41E-10	0.239226	-0.3865	-0.62572
C11orf96	0.000214	0.01525	1.27E-06	0.321924	-0.27475	-0.59667
C12orf4	9.47E-08	6.37E-08	2.31E-11	0.318773	-0.36667	-0.68545
C12orf5	4.11E-07	2.98E-12	1.57E-11	0.652547	-0.96885	-1.6214
C12orf75	2.16E-07	5.00E-09	2.38E-11	0.817039	-1.05633	-1.87337
C14orf13	0.243262	1.38E-06	1.28E-07	-0.08998	0.481075	0.571055
C15orf43	0.789185	0.000144	4.08E-06	0.017023	0.514939	0.497915
C15orf48	4.36E-08	1.62E-07	1.57E-11	0.962543	-1.36771	-2.33025
C15orf52	0.008205	0.005168	1.70E-07	0.251987	-0.32447	-0.57645
C16orf54	3.61E-09	4.67E-07	1.70E-11	0.858306	-0.81117	-1.66948
C17orf67	0.015305	1.13E-05	5.73E-09	-0.18163	0.338528	0.52016
C1orf106	0.023556	1.34E-05	8.13E-10	0.264149	-1.03084	-1.29499
C1orf162	1.73E-05	1.29E-05	3.58E-10	0.341705	-0.46506	-0.80676
C1orf198	0.00047	1.77E-07	4.72E-11	0.300412	-0.66498	-0.96539
C1orf216	4.90E-05	0.010949	1.23E-09	0.365339	-0.26672	-0.63206

C1orf54	8.05E-07	1.10E-06	1.79E-10	0.40577	-0.56916	-0.97493
C1QA	5.70E-05	3.74E-05	2.30E-09	0.382279	-0.47056	-0.85284
C1QB	5.66E-06	8.10E-06	4.72E-11	0.456586	-0.63986	-1.09645
C1QC	5.42E-07	0.000189	2.66E-10	0.441943	-0.40042	-0.84236
C1R	0.195725	7.59E-08	6.26E-08	-0.05406	0.471725	0.525786
C3AR1	4.87E-09	7.57E-06	1.70E-11	0.543291	-0.45659	-0.99988
C3P1	3.49E-05	3.53E-10	1.85E-11	-0.22786	0.651743	0.8796
C4orf48	0.002778	6.10E-10	2.96E-11	0.182137	-0.57143	-0.75356
C5AR1	0.000412	2.31E-06	4.97E-11	0.332175	-0.59495	-0.92713
C5orf27	0.339892	5.73E-05	1.79E-06	-0.33709	1.635289	1.97238
C6	0.036005	1.08E-08	1.00E-09	-0.08608	0.453343	0.539424
C7	1.20E-06	0.001322	4.67E-09	0.572392	-0.57869	-1.15108
C7orf31	8.99E-06	0.000211	1.48E-10	0.320166	-0.27375	-0.59391
C7orf55	0.002498	3.07E-06	7.00E-10	-0.18713	0.366005	0.553134
C8A	0.000183	5.86E-08	1.21E-10	-0.1969	0.472061	0.668965
C8B	0.020901	1.97E-08	1.01E-10	-0.16324	0.486112	0.649349
C8orf46	0.738611	7.59E-08	1.96E-07	-0.02309	0.499167	0.522253
C9orf152	0.041384	0.006112	2.89E-06	0.27219	-0.69523	-0.96742
CACNA1	0.000559	8.31E-10	2.68E-11	-0.19982	0.365581	0.565397
CALHM2	1.24E-07	1.58E-09	1.57E-11	0.282232	-0.40355	-0.68578
CAP1	2.23E-06	2.51E-11	1.57E-11	0.206467	-0.36441	-0.57087
CAP2	0.000357	2.29E-05	1.27E-10	0.379671	-0.54705	-0.92672
CAPG	0.000817	8.73E-07	4.97E-11	0.349186	-0.8114	-1.16058
CAPN2	0.000506	3.66E-07	2.19E-10	0.262515	-0.45158	-0.7141
CAPN3	0.099526	6.85E-05	2.13E-06	-0.16501	0.504254	0.669268
CAPN5	0.375158	2.09E-07	3.18E-08	-0.06556	0.458216	0.523779
CAPNS1	0.00022	1.56E-05	1.23E-09	0.233732	-0.31049	-0.54423
CARD16	2.16E-07	8.14E-12	1.57E-11	0.323814	-0.62334	-0.94715
CARD6	7.02E-06	9.93E-09	1.57E-11	0.287528	-0.42912	-0.71665
CASC10	0.237804	5.44E-09	4.67E-09	-0.07881	0.517406	0.596211
CASP1	2.91E-09	2.98E-12	1.57E-11	0.499988	-0.79719	-1.29717
CASP4	6.84E-05	1.37E-07	3.48E-11	0.276668	-0.41498	-0.69165
CAV2	0.313409	2.68E-06	4.03E-09	0.116503	-0.40741	-0.52391
CBFA2T3	0.329836	7.57E-06	8.90E-07	-0.11837	0.474057	0.592432
CBLN4	0.159189	6.97E-07	6.01E-10	-0.11332	0.400463	0.513781
CBS	6.84E-05	9.89E-08	7.95E-11	-0.21726	0.387696	0.604953
CCDC102B	0.078071	2.93E-05	4.71E-08	0.138203	-0.56433	-0.70254
CCDC109B	2.02E-08	9.89E-08	2.38E-11	0.461212	-0.65897	-1.12018
CCDC146	1.77E-05	2.91E-09	1.57E-11	0.458033	-1.06229	-1.52032
CCDC3	0.003419	0.000447	4.07E-08	0.235997	-0.37214	-0.60814
CCDC80	8.72E-05	0.000495	3.45E-09	0.229229	-0.32709	-0.55632
CCL14	0.003011	1.06E-05	1.29E-09	-0.234	0.456806	0.690803
CCL18	3.25E-06	0.001043	4.19E-10	0.759524	-0.78322	-1.54274
CCL19	1.87E-07	0.000293	5.62E-11	1.525011	-1.12727	-2.65228
CCL2	9.18E-09	1.01E-09	1.57E-11	0.789567	-1.11465	-1.90421
CCL20	5.53E-05	1.06E-10	1.60E-11	0.74879	-2.23191	-2.9807
CCL21	4.82E-07	1.13E-05	5.42E-11	1.047432	-1.16348	-2.21091
CCL3	8.66E-07	2.93E-05	1.00E-09	0.695809	-0.77796	-1.47377
CCL3L1	8.66E-07	2.93E-05	1.00E-09	0.695809	-0.77796	-1.47377
CCL3L3	8.66E-07	2.93E-05	1.00E-09	0.695809	-0.77796	-1.47377
CCL4	2.16E-08	2.49E-06	7.58E-11	0.720344	-0.84208	-1.56243
CCL5	2.82E-08	1.48E-07	1.82E-11	0.830259	-0.85257	-1.68283
CCL8	5.87E-05	3.12E-05	8.59E-10	0.736104	-1.02767	-1.76378
CCNA2	2.58E-05	5.97E-07	2.31E-11	0.422043	-0.88561	-1.30765
CCNB1	0.000506	7.36E-09	1.91E-11	0.341774	-1.06659	-1.40837
CCND1	0.00052	0.000423	6.01E-10	0.53938	-0.5666	-1.10598

CCNE2	0.000238	2.00E-06	4.45E-09	0.19523	-0.52819	-0.72342
CCR1	3.25E-06	0.000109	9.52E-10	0.37821	-0.43952	-0.81773
CCR2	1.10E-09	4.16E-08	1.64E-11	0.464722	-0.50833	-0.97306
CCR5	1.24E-07	3.66E-07	1.72E-11	0.611187	-0.67565	-1.28684
CCR7	4.13E-06	0.012567	1.41E-10	0.452874	-0.37545	-0.82832
CCRL2	0.00011	3.74E-05	1.59E-09	0.340271	-0.58379	-0.92406
CCS	0.002021	3.24E-09	1.64E-11	-0.18236	0.45245	0.634808
CCT6A	0.014018	1.06E-10	1.27E-10	0.176471	-0.40007	-0.57654
CD163	0.000281	0.008079	9.52E-09	0.359585	-0.29853	-0.65812
CD163L1	0.009859	4.49E-05	4.91E-09	0.233822	-0.48947	-0.72329
CD1E	2.02E-07	0.305266	2.98E-07	0.422162	-0.17331	-0.59547
CD2	4.42E-09	2.39E-09	1.57E-11	0.838478	-0.91418	-1.75265
CD200	0.000392	0.000115	1.11E-08	0.232164	-0.42577	-0.65793
CD226	0.006506	0.00199	6.50E-07	0.18155	-0.33993	-0.52148
CD24	7.18E-07	3.66E-07	2.68E-11	0.939398	-1.32566	-2.26506
CD247	2.55E-06	0.000103	1.43E-09	0.408987	-0.40353	-0.81252
CD27	3.97E-07	2.00E-06	2.96E-11	0.612245	-0.78168	-1.39392
CD300LF	1.77E-05	2.49E-06	2.68E-11	0.517055	-0.7854	-1.30245
CD38	1.38E-05	1.60E-06	1.15E-10	0.393429	-0.76838	-1.16181
CD3D	1.99E-08	1.78E-08	1.57E-11	0.655444	-0.89176	-1.5472
CD3G	0.000114	1.73E-06	5.42E-11	0.342469	-0.6318	-0.97427
CD44	8.15E-09	4.37E-06	1.69E-11	0.30323	-0.33491	-0.63814
CD48	6.58E-09	8.10E-06	1.72E-11	0.50738	-0.42961	-0.93699
CD52	7.73E-09	7.36E-09	1.64E-11	0.85242	-0.9544	-1.80682
CD53	3.36E-06	5.79E-06	7.58E-11	0.25126	-0.30383	-0.55509
CD58	1.73E-07	6.10E-10	1.57E-11	0.270466	-0.37725	-0.64772
CD5L	1.77E-05	0.289685	2.66E-06	0.445797	-0.20393	-0.64972
CD69	7.43E-09	6.37E-08	2.21E-11	0.776376	-0.8436	-1.61998
CD72	9.97E-07	0.000236	6.94E-11	0.863686	-0.82985	-1.69354
CD74	9.58E-07	1.73E-06	7.58E-11	0.292396	-0.4179	-0.71029
CD83	2.74E-05	8.17E-05	2.92E-10	0.354603	-0.49052	-0.84513
CD86	1.94E-07	0.00018	1.79E-10	0.426551	-0.44977	-0.87632
CD8A	3.70E-09	5.44E-09	1.57E-11	0.960547	-1.11049	-2.07104
CD96	1.77E-05	2.65E-09	1.60E-11	0.285528	-0.62092	-0.90645
CD97	6.22E-08	2.89E-07	2.21E-11	0.478461	-0.48849	-0.96695
CDA	0.012307	0.001095	7.59E-08	-0.25192	0.397409	0.649326
CDC20	0.018194	9.89E-08	1.41E-10	0.280623	-1.06538	-1.346
CDC25B	0.000183	6.09E-05	1.43E-09	0.28452	-0.39398	-0.6785
CDC42EP3	6.94E-08	5.97E-07	3.07E-11	0.361942	-0.41738	-0.77933
CDC42EP5	0.004376	0.05377	2.79E-05	0.224061	-0.33454	-0.5586
CDC42SE1	0.000348	5.39E-05	8.13E-10	0.223962	-0.28467	-0.50863
CDC42SE2	5.09E-06	9.89E-06	2.96E-11	0.222051	-0.31919	-0.54124
CDC45	0.017877	1.28E-06	6.68E-10	0.151578	-0.53727	-0.68885
CDC6	0.031573	2.18E-09	3.07E-10	0.202736	-1.23233	-1.43506
CDC7	2.25E-07	1.78E-11	1.57E-11	0.504037	-1.11007	-1.61411
CDCA3	0.029467	2.58E-05	3.87E-08	0.1477	-0.48228	-0.62998
CDCA5	0.011857	1.10E-06	1.36E-09	0.207187	-0.5652	-0.77239
CDCA7L	0.015866	0.000667	1.87E-07	0.200384	-0.31818	-0.51856
CDH11	0.002065	3.74E-05	1.87E-09	0.198908	-0.40691	-0.60582
CDHR2	0.123413	0.090918	0.000164	0.28828	-0.39173	-0.68001
CDHR5	0.989666	2.15E-06	1.44E-06	-0.00533	0.557153	0.562478
CDK1	0.000329	5.86E-08	2.87E-11	0.347643	-0.87295	-1.22059
CDK3	0.026568	4.31E-07	5.16E-10	-0.1525	0.363694	0.516196
CDKN1A	0.000226	9.69E-05	2.66E-10	0.52066	-0.53737	-1.05803

CDKN1C	0.000644	0.001517	2.15E-07	0.230811	-0.30428	-0.53509
CDKN2C	0.000259	8.10E-06	3.45E-09	0.29663	-0.38524	-0.68187
CDKN3	0.000412	1.26E-07	1.70E-11	0.53367	-1.15027	-1.68394
CDO1	0.180039	3.14E-07	7.01E-09	-0.08932	0.476803	0.566125
CECR1	6.54E-09	2.43E-05	2.58E-11	0.707656	-0.64328	-1.35093
CECR2	0.138825	2.35E-08	1.51E-08	-0.064	0.49728	0.561283
CELF2	5.80E-08	5.04E-06	2.13E-11	0.389818	-0.36969	-0.7595
CENPA	0.165459	3.98E-05	1.41E-07	0.088008	-0.46793	-0.55594
CENPE	0.043338	4.70E-06	8.19E-09	0.169362	-0.61457	-0.78393
CENPH	0.001294	9.47E-07	3.07E-11	0.297117	-0.52596	-0.82308
CENPK	8.17E-08	3.82E-06	1.72E-11	0.637558	-1.00418	-1.64174
CENPM	0.002107	3.66E-07	4.19E-10	0.28097	-0.6408	-0.92177
CENPU	2.08E-06	3.79E-06	1.95E-11	0.574181	-0.84726	-1.42144
CENPW	0.002107	1.16E-07	1.01E-10	0.304118	-0.75864	-1.06276
CEP55	0.050946	7.57E-06	2.89E-08	0.131632	-0.62845	-0.76009
CES3	0.006506	5.36E-08	1.96E-09	-0.14037	0.455295	0.595665
CES4A	0.126878	1.86E-06	1.36E-08	-0.13073	0.493607	0.624332
CES5A	0.803395	1.02E-06	5.70E-08	-0.0366	0.650155	0.686754
CFD	0.000266	0.00047	2.14E-08	0.403854	-0.40864	-0.8125
CFHR3	0.829996	7.28E-05	4.82E-06	-0.09256	0.641016	0.733574
CFHR4	0.621852	9.17E-05	3.01E-05	-0.03556	0.78869	0.824254
CFHR5	0.198047	8.20E-09	5.72E-10	-0.10619	0.820779	0.926964
CFTR	2.43E-05	3.99E-07	4.91E-10	0.178919	-0.3502	-0.52912
CGN	0.033731	2.48E-10	9.01E-10	-0.17299	0.631567	0.804553
CH17-132F21.1	6.03E-07	0.000575	5.45E-10	0.599582	-0.64625	-1.24583
CH25H	0.000101	2.46E-07	1.64E-10	0.466501	-1.09315	-1.55965
CHAD	0.031057	4.67E-07	2.81E-09	-0.16139	0.482806	0.644194
CHEK2	0.180039	0.000605	5.69E-06	0.145654	-0.47461	-0.62026
CHI3L1	0.259771	0.050673	0.000902	0.329198	-0.71074	-1.03994
CHMP3	4.48E-05	2.39E-09	2.78E-11	0.198818	-0.34844	-0.54726
CHRDL1	0.002605	0.884498	0.000527	0.552365	0.022068	-0.5303
CHST11	2.16E-08	1.61E-08	1.57E-11	0.308184	-0.42774	-0.73592
CHST4	4.29E-06	5.39E-06	1.88E-10	0.766843	-1.04197	-1.80881
CHST9	0.010626	2.46E-07	1.72E-10	0.478286	-1.15251	-1.63079
CHSY1	5.02E-07	3.99E-07	2.49E-11	0.419516	-0.40651	-0.82603
CIDEB	0.003492	2.81E-10	3.48E-11	-0.14596	0.451538	0.597494
CKAP2	3.61E-09	2.67E-07	1.01E-10	0.389639	-0.54645	-0.93609
CKLF	3.32E-08	2.18E-09	4.10E-11	0.302552	-0.49373	-0.79628
CKLF-CMTM1	1.17E-05	1.61E-08	5.92E-11	0.334424	-0.5197	-0.85413
CKMT2	0.046156	0.006897	3.60E-07	0.266854	-0.27742	-0.54427
CKS2	2.58E-05	3.39E-07	7.28E-11	0.526557	-0.78298	-1.30954
CLDN10	2.80E-05	0.000249	1.06E-08	0.468473	-0.57347	-1.04194
CLDN11	2.43E-05	1.58E-09	1.69E-11	0.275012	-0.54656	-0.82157
CLDN14	0.07627	5.00E-09	1.29E-09	-0.12412	0.759695	0.883815
CLDN7	0.014508	8.07E-07	1.99E-10	0.246508	-0.68749	-0.93399
CLEC11A	7.94E-05	0.0007	2.47E-07	0.219288	-0.31091	-0.5302
CLEC2B	0.000838	0.000293	6.68E-09	0.276529	-0.3711	-0.64763
CLEC2D	1.14E-07	8.20E-09	1.57E-11	0.23425	-0.41862	-0.65287
CLEC4A	6.03E-09	6.47E-07	1.58E-11	0.418479	-0.53094	-0.94942
CLEC7A	8.95E-08	5.79E-06	2.13E-11	0.319789	-0.37269	-0.69248
CLIC1	8.95E-07	1.98E-10	2.21E-11	0.281256	-0.4936	-0.77485
CLIC2	7.27E-06	0.000401	1.56E-10	0.43801	-0.3838	-0.82181
CLIC3	0.003354	0.035109	1.51E-06	0.317455	-0.27342	-0.59088
CLIC6	0.003865	7.48E-07	4.19E-10	0.272963	-0.75028	-1.02324
CLIP4	3.49E-05	0.000136	2.56E-10	0.324869	-0.3539	-0.67877
CMBL	0.003492	6.70E-10	1.01E-10	-0.1295	0.414511	0.544013

CMPK2	5.37E-05	0.006364	5.73E-09	0.565068	-0.54237	-1.10744
CMTM7	8.43E-08	7.36E-09	1.57E-11	0.283332	-0.33658	-0.61991
CMTM8	0.008345	6.28E-11	1.27E-10	-0.12553	0.482992	0.608523
CNDP1	0.295169	3.12E-08	8.59E-10	-0.18233	1.323628	1.505962
CNGA1	0.131933	1.02E-06	2.85E-07	-0.15517	0.583313	0.738485
CNOT6	1.61E-06	0.001816	6.01E-10	0.32789	-0.18923	-0.51712
CNRIP1	7.82E-06	8.17E-05	2.19E-10	0.346366	-0.32628	-0.67264
COBLL1	0.418678	3.45E-08	1.11E-06	0.064534	0.524695	0.460161
COL15A1	0.029978	0.181223	1.39E-05	0.456686	-0.22917	-0.68586
COL16A1	0.003147	0.000136	3.52E-08	0.281537	-0.4855	-0.76704
COL1A1	1.13E-05	7.37E-10	1.57E-11	0.410522	-0.82676	-1.23729
COL1A2	1.14E-07	4.45E-10	1.57E-11	0.7349	-1.2275	-1.9624
COL3A1	1.10E-08	5.00E-09	1.57E-11	0.705597	-0.88923	-1.59483
COL4A1	0.000124	9.09E-09	1.82E-11	0.430439	-0.98642	-1.41685
COL4A2	0.000612	1.75E-09	1.57E-11	0.370516	-1.03326	-1.40377
COL4A4	0.000173	0.00018	9.52E-09	0.259035	-0.35667	-0.61571
COL5A1	5.06E-05	2.65E-09	1.95E-11	0.358063	-0.87681	-1.23488
COL5A2	0.00047	2.46E-07	9.63E-11	0.330817	-0.65552	-0.98634
COL6A2	0.003956	0.003375	5.94E-07	0.220567	-0.32495	-0.54552
COL6A3	3.14E-06	0.000136	7.00E-10	0.450652	-0.51113	-0.96178
COMMD8	0.010441	6.20E-06	2.92E-10	0.191146	-0.35584	-0.54698
COQ10A	0.364455	3.12E-08	2.68E-09	-0.07593	0.565569	0.641498
CORO1A	2.50E-09	8.20E-09	1.57E-11	0.688417	-0.73065	-1.41907
CORO1C	0.00011	1.08E-08	7.95E-11	0.278688	-0.467	-0.74569
COTL1	5.87E-09	6.66E-06	2.38E-11	0.38442	-0.39009	-0.77451
COX7A1	0.151509	0.003526	9.69E-06	0.197814	-0.3126	-0.51041
CPA3	0.07963	0.218279	0.000846	0.330455	-0.36315	-0.69361
CPS1-IT1	0.870885	0.000947	0.000358	0.037056	0.638574	0.601518
CPVL	3.55E-07	2.29E-05	7.95E-11	0.504477	-0.49027	-0.99475
CRIM1	0.000376	0.000103	2.18E-09	0.36483	-0.40908	-0.77391
CRIP1	6.54E-09	0.000161	2.49E-11	0.806096	-0.54103	-1.34713
CRISPLD2	0.002547	8.67E-05	4.67E-09	0.200177	-0.33259	-0.53277
CRLF3	8.67E-08	1.61E-08	1.69E-11	0.33984	-0.39442	-0.73426
CRP	0.564675	0.378944	0.038931	0.213756	-0.38394	-0.5977
CRTAM	4.87E-09	8.10E-06	1.85E-11	0.765792	-0.68309	-1.44888
CRYAB	0.001494	0.001816	4.45E-09	0.49313	-0.4587	-0.95183
CRYBG3	2.58E-05	4.70E-06	1.27E-10	0.292405	-0.3149	-0.6073
CSF1R	7.51E-05	0.0002	1.06E-09	0.333485	-0.3871	-0.72059
CSF2RB	2.50E-09	9.89E-06	1.57E-11	0.793921	-0.67802	-1.47194
CST7	1.73E-07	1.66E-05	1.88E-10	0.541383	-0.59363	-1.13501
CTD-2284J15.1	0.985643	1.02E-06	4.75E-07	0.001177	0.546003	0.544826
CTD-3080P12.	0.005249	4.77E-05	4.80E-09	-0.27026	0.404381	0.674644
CTD-3193O13.1	0.086703	3.29E-06	1.29E-08	-0.14378	0.406159	0.549936
CTGF	0.019458	7.59E-08	8.59E-10	0.349155	-1.00116	-1.35031
CTHRC1	0.007314	7.02E-08	1.21E-10	0.272561	-1.12083	-1.39339
CTNNA3	0.053563	1.51E-10	3.48E-11	-0.1864	0.632621	0.819018
CTR9	0.004844	1.90E-05	3.52E-08	0.240449	-0.35326	-0.59371
CTSA	0.00012	1.86E-06	3.23E-10	0.215178	-0.34677	-0.56195
CTSC	2.50E-09	3.63E-09	1.57E-11	0.521155	-0.71698	-1.23814
CTSD	0.001722	0.000324	2.05E-07	0.233725	-0.28346	-0.51718
CTSK	7.27E-06	6.09E-05	3.48E-11	0.427958	-0.46497	-0.89293
CTSS	2.87E-08	2.15E-05	1.95E-11	0.568779	-0.49983	-1.06861
CTTNBP2NL	5.15E-08	5.36E-08	1.69E-11	0.310075	-0.3542	-0.66428

CUX2	0.433505	0.001816	9.31E-06	-0.24625	0.879356	1.125604
CXCL1	1.51E-05	8.26E-08	1.57E-11	0.419573	-0.80414	-1.22371
CXCL10	1.60E-07	4.31E-07	1.69E-11	1.496121	-1.65118	-3.1473
CXCL11	1.29E-05	1.21E-05	4.10E-11	1.162141	-1.69999	-2.86213
CXCL12	4.48E-05	6.20E-06	2.92E-10	0.295234	-0.3662	-0.66143
CXCL13	0.005771	0.000381	4.75E-07	0.51657	-1.25002	-1.76659
CXCL16	1.45E-06	0.000249	3.40E-10	0.341164	-0.30445	-0.64562
CXCL2	0.09815	3.98E-05	1.28E-07	-0.22933	0.549632	0.778964
CXCL6	1.94E-07	3.63E-09	1.60E-11	1.207842	-1.80193	-3.00977
CXCL8	0.000214	6.82E-12	1.57E-11	0.338272	-1.26267	-1.60095
CXCL9	9.58E-07	3.74E-05	2.58E-11	1.54093	-1.40556	-2.94649
CXCR4	8.05E-10	2.91E-09	1.57E-11	0.717908	-0.80562	-1.52353
CXCR6	0.003865	2.89E-07	3.23E-10	0.166936	-0.46817	-0.63511
CXorf23	1.61E-05	0.018189	5.17E-09	0.386994	-0.20268	-0.58968
CXorf38	2.14E-06	9.69E-05	1.79E-10	0.275097	-0.25544	-0.53053
CYAT1	4.64E-07	4.22E-05	5.45E-10	0.300084	-0.30563	-0.60571
CYBRD1	0.000367	0.000115	9.52E-09	0.353444	-0.42219	-0.77563
CYP11A1	0.061401	8.10E-06	7.79E-09	0.140666	-0.50479	-0.64545
CYP1A1	0.066051	2.43E-05	0.004246	0.458662	1.261184	0.802521
CYP1A2	0.630821	4.91E-08	2.05E-07	0.062668	0.901957	0.839289
CYP26A1	0.382123	0.002077	0.000129	-0.29809	0.963026	1.261119
CYP2A13	0.004376	0.001517	1.23E-08	-0.31558	0.391216	0.706795
CYP2A6	0.000117	9.69E-05	1.79E-10	-0.4521	0.544274	0.996376
CYP2A7	0.000226	0.000144	1.79E-10	-0.47015	0.535706	1.005851
CYP2C19	0.378707	1.02E-06	5.68E-07	-0.10783	1.641283	1.749117
CYP2D7P	0.09815	0.305266	0.002826	-0.31601	0.281515	0.597521
CYP39A1	0.044713	1.16E-07	6.34E-09	-0.11133	0.520598	0.63193
CYP3A4	0.202778	3.78E-08	1.29E-09	-0.0815	0.687459	0.768958
CYP3A43	0.396353	8.26E-08	6.68E-09	-0.08643	0.693303	0.779732
CYP3A7	0.144149	0.014191	4.53E-05	0.168648	-0.37254	-0.54118
CYP4A11	0.112549	1.37E-07	6.68E-10	-0.15894	0.65405	0.812989
CYP4A22	0.169737	2.15E-06	2.54E-09	-0.17165	0.647687	0.819336
CYP4F12	0.002723	1.56E-05	4.91E-09	-0.23452	0.424164	0.658684
CYP4F3	0.207421	2.15E-08	8.62E-09	-0.04923	0.454967	0.504199
CYP7A1	0.993714	2.15E-05	6.71E-06	0.000509	2.346723	2.346213
CYR61	0.003708	1.48E-06	7.28E-11	0.439079	-0.84033	-1.27941
CYS1	0.000986	0.056842	2.40E-05	0.325768	-0.31087	-0.63664
CYSLTR1	2.62E-07	2.43E-05	3.07E-11	0.288778	-0.29624	-0.58502
CYTIP	2.44E-10	9.93E-09	1.57E-11	0.791313	-0.77497	-1.56628
DACT1	3.93E-05	0.005863	1.11E-08	0.388687	-0.41804	-0.80673
DAK	0.071056	1.66E-05	1.28E-07	-0.14569	0.50573	0.651424
DBF4	3.55E-07	3.14E-07	4.29E-11	0.431878	-0.52007	-0.95194
DBH-AS1	0.031573	0.002077	4.08E-06	-0.21827	0.536467	0.754737
DBN1	0.004031	0.000153	2.36E-08	0.166793	-0.35447	-0.52126
DBNDD2	1.62E-08	4.06E-06	5.17E-11	0.386218	-0.38794	-0.77416
DCDC1	0.73427	0.003836	0.000125	-0.10961	0.563417	0.673025
DCDC2	1.77E-05	1.97E-08	2.38E-11	0.455946	-0.95325	-1.40919
DCDC5	0.73427	0.003836	0.000125	-0.10961	0.563417	0.673025
DCK	1.28E-07	1.08E-08	1.82E-11	0.543014	-0.64818	-1.19119
DCN	0.000198	9.89E-06	1.15E-10	0.21293	-0.34702	-0.55995
DDB2	1.45E-06	0.227278	1.01E-08	0.614081	-0.13404	-0.74812
DDIAS	0.019336	1.06E-05	2.36E-08	0.146619	-0.41305	-0.55967
DDX26B	1.79E-08	6.66E-06	5.62E-11	0.554931	-0.55606	-1.11099
DDX39A	8.05E-07	1.16E-10	1.57E-11	0.246994	-0.39229	-0.63928
DDX60	1.95E-05	0.001666	1.78E-09	0.479409	-0.45467	-0.93408
DEFB1	0.538916	0.000423	0.000511	-0.12396	-0.8839	-0.75994
DENND1	0.000778	9.69E-05	7.79E-09	0.201428	-0.33449	-0.53592

DENND2D	0.00245	0.000211	1.96E-09	0.302211	-0.31801	-0.62022
DENND6A	3.48E-06	0.0009	4.91E-10	0.341665	-0.26679	-0.60846
DEPDC1	0.414886	5.73E-05	3.29E-06	0.025538	-0.49306	-0.5186
DEPDC1B	0.000986	1.12E-09	1.57E-11	0.23844	-0.86045	-1.09889
DFNA5	0.005359	1.66E-05	3.45E-09	0.22644	-0.38138	-0.60782
DGAT2	0.038913	8.76E-12	1.91E-11	-0.17003	0.708549	0.878582
DHRS1	0.019458	7.74E-05	1.51E-08	-0.16821	0.405858	0.574073
DHRS2	0.433505	0.003375	0.000134	-0.2205	0.957068	1.177568
DHRS4-AS1	0.008995	9.09E-09	6.94E-11	-0.14147	0.485306	0.626779
DHRS9	1.25E-05	1.28E-06	3.21E-11	0.488552	-0.72201	-1.21056
DIRAS3	0.091674	0.001737	7.29E-06	-0.24274	0.632693	0.875437
DKFZp667J0810	5.51E-09	2.03E-05	1.21E-10	1.185568	-1.1474	-2.33297
DKK3	0.000208	2.68E-06	1.01E-10	0.308923	-0.61669	-0.92561
DLEU1	0.118714	7.57E-06	5.70E-08	-0.15297	0.466688	0.619655
DLGAP5	2.14E-05	6.60E-06	4.72E-11	0.441396	-1.05511	-1.49651
DMGDH	0.050444	1.26E-07	2.04E-08	-0.06835	0.49762	0.565972
DMXL2	0.000493	0.002378	2.36E-07	0.24361	-0.25783	-0.50144
DNAJC12	0.002547	2.31E-06	2.79E-10	-0.31687	0.578047	0.894913
DNAJC25	0.065076	3.42E-11	1.85E-11	-0.12126	0.627923	0.749184
DNAJC25-GNG10	0.065076	3.42E-11	1.85E-11	-0.12126	0.627923	0.749184
DNAJC5B	0.013013	4.67E-07	4.67E-09	0.154487	-0.5637	-0.71818
DNM1	0.008345	0.21184	0.000121	-0.42307	0.238342	0.661411
DNM3OS	4.92E-06	0.006112	2.68E-09	0.36616	-0.34304	-0.7092
DNMT1	7.49E-09	3.52E-07	2.87E-11	0.435626	-0.42625	-0.86187
DNMT3L	0.034876	5.51E-07	2.08E-10	-0.33002	0.887776	1.217795
DOCK10	1.38E-07	0.000992	4.29E-11	0.395929	-0.2851	-0.68103
DOCK11	6.52E-09	2.03E-05	1.69E-11	0.402957	-0.42029	-0.82324
DOCK2	1.45E-06	1.56E-05	3.73E-11	0.255316	-0.308	-0.56331
DOCK8	5.80E-08	1.48E-08	1.85E-11	0.37476	-0.44894	-0.8237
DOK2	3.69E-06	5.39E-05	1.06E-09	0.301493	-0.33212	-0.63361
DOK3	2.88E-05	6.09E-05	1.88E-10	0.231988	-0.30863	-0.54061
DPT	1.50E-07	0.002077	4.91E-10	0.794786	-0.71146	-1.50625
DPYSL2	1.60E-07	5.97E-07	1.77E-11	0.468996	-0.42278	-0.89177
DRAM1	3.70E-09	3.12E-08	1.57E-11	0.529345	-0.57292	-1.10227
DRAM2	0.000944	2.58E-05	4.67E-09	0.259411	-0.28532	-0.54473
DSG1	0.954983	1.48E-08	6.88E-08	0.047833	0.920055	0.872223
DSN1	0.003708	1.08E-08	5.17E-11	0.166469	-0.50205	-0.66851
DTL	0.000117	1.12E-09	1.82E-11	0.448471	-1.22269	-1.67116
DUSP2	0.006779	0.000121	8.19E-09	0.200105	-0.33264	-0.53275
DYNLT1	1.04E-06	3.39E-12	1.85E-11	0.254383	-0.49936	-0.75374
DZIP1	0.001758	1.21E-05	1.23E-09	0.237693	-0.43933	-0.67702
E2F3	0.006643	3.53E-10	5.17E-11	0.143759	-0.43901	-0.58277
E2F8	5.06E-05	4.67E-07	1.68E-11	0.458699	-1.1206	-1.57929
EBI3	0.008827	0.001666	1.48E-07	0.216544	-0.37469	-0.59123
EBPL	8.12E-06	2.87E-06	1.15E-10	-0.2308	0.375215	0.606015
ECHDC2	0.346657	7.48E-07	5.97E-08	-0.1056	0.627292	0.732888
ECT2	0.001326	1.16E-07	3.07E-11	0.154647	-0.35828	-0.51293
EDNRB	0.000402	0.002273	4.49E-08	0.306633	-0.23851	-0.54514
EFCAB7	0.00245	0.012232	4.12E-07	0.317214	-0.2824	-0.59962
EFEMP1	6.26E-07	5.86E-08	1.95E-11	0.609605	-1.03152	-1.64112
EFEMP2	0.000402	6.85E-05	8.62E-09	0.258947	-0.40239	-0.66134
EFHD1	0.087816	2.75E-05	7.59E-08	-0.3082	0.954922	1.263127
EFNA1	0.041384	0.009067	1.21E-06	-0.25618	0.403511	0.659687

EGR1	0.670883	0.00746	0.002011	0.135919	-0.79918	-0.9351
EGR2	5.36E-08	1.66E-05	3.48E-11	0.55883	-0.75063	-1.30946
EGR3	0.019115	2.00E-06	8.13E-10	0.200052	-0.48591	-0.68596
ELF3	0.00011	5.73E-05	1.17E-09	0.289028	-0.40512	-0.69415
ELF4	9.13E-08	7.57E-06	4.10E-11	0.348512	-0.37038	-0.71889
ELOVL1	0.000169	2.16E-11	2.43E-11	0.200547	-0.41529	-0.61583
ELOVL2	0.010243	1.66E-05	1.94E-08	0.273476	-0.49063	-0.7641
ELOVL7	3.97E-06	6.47E-07	4.10E-11	0.712269	-1.13806	-1.85032
EMILIN1	0.001192	0.000547	1.32E-06	0.24095	-0.35083	-0.59178
EMP3	1.21E-05	4.06E-06	1.68E-09	0.356012	-0.41215	-0.76816
EMR1	0.003572	0.09585	4.88E-05	0.417074	-0.40663	-0.82371
EMR2	1.44E-07	0.009432	2.79E-10	0.429646	-0.32796	-0.75761
ENDOD1	1.83E-05	2.67E-07	8.66E-11	0.293158	-0.51108	-0.80424
ENHO	0.052034	9.05E-08	7.60E-10	-0.24403	0.671875	0.915906
ENPP1	0.600397	2.48E-10	1.29E-09	-0.02253	0.546307	0.568837
ENPP2	0.000376	7.10E-06	9.01E-10	0.575363	-0.801	-1.37636
ENPP5	0.00465	1.47E-05	3.97E-10	0.229251	-0.70093	-0.93018
ENPP7	0.001722	0.001901	5.94E-07	0.307968	-0.34526	-0.65323
ENTPD1	1.38E-08	3.93E-10	1.57E-11	0.368809	-0.65299	-1.0218
EOGT	1.80E-06	1.77E-07	1.27E-10	0.41598	-0.47519	-0.89117
EOMES	1.02E-07	0.000992	1.27E-10	0.719179	-0.4908	-1.20998
EPB41L2	7.47E-07	1.29E-05	4.72E-11	0.268339	-0.25704	-0.52538
EPB41L3	5.60E-07	2.31E-06	1.70E-11	0.519936	-0.56399	-1.08393
EPCAM	2.14E-06	1.06E-05	1.99E-10	1.270117	-1.89577	-3.16589
EPDR1	3.36E-06	1.48E-08	2.04E-11	0.607969	-0.79346	-1.40143
EPHA3	0.000134	0.00018	1.36E-09	0.269722	-0.32004	-0.58976
EPHB1	0.012307	5.07E-05	3.28E-09	-0.28995	0.490014	0.779969
EPHX2	0.008613	2.34E-11	7.28E-11	-0.17171	0.582237	0.753942
EPSTI1	6.26E-07	0.000737	3.78E-10	0.673861	-0.58706	-1.26092
ERAP2	0.006506	0.316607	0.001462	0.397428	-0.11197	-0.50939
ERI1	5.46E-06	0.000211	5.45E-10	0.274509	-0.2452	-0.51971
ERICH5	0.040115	0.000103	2.15E-07	0.250651	-0.8013	-1.05195
ERMP1	0.000434	3.98E-05	1.87E-09	0.232041	-0.30376	-0.5358
ERP27	0.021653	8.07E-07	6.01E-10	0.167309	-0.51906	-0.68637
ERRFI1	0.036612	4.37E-06	1.11E-08	-0.17354	0.636877	0.810416
ESRP1	0.10091	0.001095	1.71E-06	0.198195	-0.6908	-0.88899
ETNK2	0.014761	1.10E-06	5.16E-10	-0.22499	0.681728	0.906719
ETV7	0.003419	3.39E-07	4.29E-11	0.274433	-0.76372	-1.03815
EVI2A	1.09E-09	4.91E-08	1.57E-11	0.821724	-0.80237	-1.62409
EVI2B	2.50E-09	1.60E-06	1.77E-11	0.670683	-0.55209	-1.22277
EXOC1	6.90E-07	1.47E-05	2.79E-10	0.295727	-0.21031	-0.50604
EXPH5	1	2.65E-09	3.52E-08	0.006607	0.515637	0.50903
EZH2	4.36E-08	1.10E-06	3.59E-11	0.65937	-0.76973	-1.4291
F11	0.212411	4.16E-08	4.07E-08	-0.13564	0.418724	0.554368
F13A1	0.042025	0.009432	1.14E-05	0.200584	-0.47059	-0.67117
F2RL1	0.006252	1.33E-08	6.68E-10	0.299516	-0.60586	-0.90538
F3	0.073191	5.73E-05	6.34E-09	0.147282	-0.44864	-0.59592
FABP3	0.003147	0.047733	1.18E-05	0.282113	-0.26943	-0.55154
FABP4	0.036005	0.088602	8.23E-06	0.288523	-0.22751	-0.51603
FABP5	5.42E-07	1.62E-07	1.85E-11	1.073957	-1.30963	-2.38358
FADS1	0.906416	0.029892	0.012631	-0.00847	-0.73118	-0.7227
FAIM	0.006506	6.10E-10	1.72E-11	0.292593	-0.83148	-1.12407
FAIM3	5.57E-08	2.15E-05	1.27E-10	0.338523	-0.37234	-0.71086
FAM102B	5.17E-09	4.91E-10	1.57E-11	0.525673	-0.56144	-1.08712
FAM111B	0.05777	0.000121	1.78E-07	0.122603	-0.52748	-0.65008
FAM122B	5.87E-06	0.023046	1.85E-08	0.37467	-0.14976	-0.52443
FAM124B	0.387824	0.002481	0.00017	-0.16297	0.453147	0.616113
FAM127A	0.285682	4.16E-08	2.25E-08	0.058268	-0.44183	-0.5001

FAM129A	1.90E-09	3.39E-07	1.57E-11	0.666797	-0.59384	-1.26064
FAM134B	0.002498	2.89E-07	4.97E-11	-0.22637	0.501228	0.727594
FAM150B	1.25E-05	0.003308	1.29E-08	0.742471	-0.69788	-1.44035
FAM151A	0.021277	0.003375	2.33E-06	-0.49205	0.828205	1.320252
FAM169A	0.001162	5.79E-06	3.23E-10	0.47977	-0.80509	-1.28486
FAM198A	0.017251	0.000249	2.75E-08	-0.42608	0.746521	1.172606
FAM213B	1.77E-07	1.33E-08	1.57E-11	0.375626	-0.47348	-0.84911
FAM26F	0.001359	0.00052	1.43E-09	0.323714	-0.53019	-0.85391
FAM3B	0.002723	2.15E-05	2.95E-09	0.574157	-1.68212	-2.25628
FAM46C	0.000726	0.003104	1.41E-07	0.280596	-0.28748	-0.56808
FAM49A	6.58E-09	2.31E-06	1.57E-11	0.421336	-0.34813	-0.76946
FAM57A	0.003085	6.97E-07	1.79E-10	0.260286	-0.54274	-0.80303
FAM60A	5.06E-05	3.03E-12	1.58E-11	0.217293	-0.50178	-0.71908
FAM65B	1.11E-06	0.000249	1.96E-09	0.399643	-0.3772	-0.77685
FAM72A	0.019115	1.06E-05	4.71E-08	0.247541	-0.71551	-0.96305
FAM72B	0.019115	1.06E-05	4.71E-08	0.247541	-0.71551	-0.96305
FAM72C	0.019115	1.06E-05	4.71E-08	0.247541	-0.71551	-0.96305
FAM72D	0.019115	1.06E-05	4.71E-08	0.247541	-0.71551	-0.96305
FAM78A	2.87E-08	1.66E-05	3.59E-11	0.397873	-0.34797	-0.74585
FAM9B	0.000412	0.00199	1.87E-09	-0.39678	0.302597	0.699377
FANCI	0.002021	3.99E-07	1.41E-10	0.192743	-0.46767	-0.66041
FAP	0.106522	6.97E-07	1.97E-05	-0.15747	-0.70627	-0.54881
FAR1	1.33E-08	9.89E-08	8.66E-11	0.337249	-0.30991	-0.64715
FAS	7.85E-08	0.004958	8.66E-11	0.582528	-0.26069	-0.84322
FAT1	3.58E-06	5.36E-08	3.73E-11	0.981608	-1.22853	-2.21014
FBLN5	2.58E-05	0.000171	1.17E-09	0.633513	-0.76635	-1.39987
FBN1	2.01E-05	9.93E-09	2.87E-11	0.341432	-0.66003	-1.00146
FBXO6	0.001239	0.010949	2.47E-07	0.300957	-0.2545	-0.55545
FCAMR	0.000159	0.010145	1.51E-08	0.950264	-0.6474	-1.59766
FCER1G	3.25E-06	6.09E-05	2.31E-10	0.486622	-0.53998	-1.02661
FCGR1A	1.56E-05	6.47E-07	2.49E-11	0.397077	-0.64778	-1.04485
FCGR1B	2.25E-07	2.15E-08	1.57E-11	0.892939	-1.35916	-2.2521
FCGR1C	5.42E-07	2.35E-08	1.60E-11	0.798109	-1.24844	-2.04655
FCGR3A	0.005249	0.000277	1.87E-07	0.393691	-0.69974	-1.09343
FCGR3B	0.003011	0.000115	8.62E-09	0.322945	-0.57236	-0.8953
FCN1	2.72E-07	2.49E-06	8.66E-11	0.595238	-0.86513	-1.46036
FCRL3	7.51E-05	0.000249	2.07E-09	0.344598	-0.42396	-0.76856
FDXR	6.25E-05	0.002273	3.23E-10	0.389212	-0.30735	-0.69656
FEN1	0.001758	8.31E-10	1.88E-10	0.223181	-0.65061	-0.87379
FERMT3	4.36E-08	4.31E-07	1.69E-11	0.396241	-0.50167	-0.89791
FGF13	0.011667	0.003104	2.25E-07	0.254454	-0.53349	-0.78795
FGF14-AS2	0.107949	3.98E-05	1.58E-08	-0.11609	0.45577	0.571863
FGL2	2.32E-08	1.26E-07	1.57E-11	0.522274	-0.56635	-1.08862
FGR	2.02E-08	7.57E-06	3.36E-11	0.518865	-0.43367	-0.95253
FHL2	0.001162	1.56E-05	6.01E-10	0.497559	-0.80619	-1.30375
FHOD1	3.97E-06	8.71E-06	2.66E-10	0.223516	-0.2894	-0.51292
FIG4	1.80E-06	5.04E-06	8.29E-11	0.254172	-0.27782	-0.532
FIGNL1	7.30E-05	0.000136	2.66E-10	0.249885	-0.269	-0.51889
FILIP1L	0.000232	2.34E-11	1.57E-11	0.234567	-0.56002	-0.79459
FITM1	0.147574	5.97E-07	1.43E-08	-0.18673	0.614001	0.80073
FJX1	0.006128	3.66E-07	1.21E-10	0.188222	-0.50861	-0.69683
FKBP11	0.008345	2.46E-07	7.70E-10	0.147769	-0.36977	-0.51754
FKBP1A-SDCBP2	0.522219	0.001205	2.79E-05	0.114173	-0.80724	-0.92141
FKBP1B	0.074119	0.00297	6.11E-05	0.173115	-0.36774	-0.54086
FLJ12120	0.013262	0.213997	0.000464	0.357477	-0.21099	-0.56847
FLJ22763	0.883163	0.000308	0.000421	0.047295	0.513045	0.46575

FLJ32255	0.000481	0.000342	2.42E-09	0.222292	-0.35912	-0.58141
FLNA	0.000709	2.29E-05	5.43E-09	0.250213	-0.38139	-0.6316
FLRT3	0.26255	3.79E-06	9.76E-05	-0.17341	-0.76787	-0.59446
FLVCR1	1.40E-06	3.98E-05	1.88E-10	0.455591	-0.48028	-0.93587
FMO1	0.042025	0.000815	1.48E-07	0.505288	-1.03158	-1.53687
FMO2	0.518097	2.15E-06	1.51E-08	0.11521	-0.73461	-0.84982
FMO5	0.92867	8.21E-11	2.07E-09	-0.0178	0.631521	0.649325
FMOD	0.069979	2.75E-05	5.97E-08	0.149065	-0.47082	-0.61988
FNDC1	0.003786	3.53E-06	1.88E-10	0.394949	-1.16278	-1.55773
FNIP2	0.003865	8.10E-06	1.43E-09	-0.30212	0.510502	0.812618
FOLH1	0.013262	6.09E-05	8.36E-08	-0.1496	0.45147	0.601071
FOLH1B	0.036005	7.28E-05	1.35E-07	-0.14159	0.573225	0.714816
FOS	0.163463	0.009432	0.000394	0.188178	-0.42774	-0.61591
FOXM1	0.011462	4.06E-06	6.01E-10	0.199827	-0.65526	-0.85509
FOXQ1	0.468785	3.53E-05	8.14E-07	0.143303	-0.9963	-1.1396
FPR3	9.47E-08	1.38E-05	1.82E-11	0.549698	-0.55531	-1.10501
FRMD3	0.009684	3.12E-05	3.23E-10	0.17555	-0.36834	-0.54389
FRMD6	9.27E-07	0.000109	1.41E-10	0.583334	-0.45333	-1.03666
FSTL3	1.03E-05	1.26E-07	1.15E-10	0.263837	-0.47963	-0.74347
FTCD	0.004206	1.01E-09	5.17E-11	-0.18762	0.527173	0.714793
FUNDC1	0.000922	0.000161	7.01E-09	0.246529	-0.27299	-0.51952
FUT4	8.05E-10	3.17E-10	1.57E-11	0.422482	-0.61347	-1.03595
FUT8	8.67E-08	1.30E-10	1.57E-11	0.271515	-0.5024	-0.77391
FXYD1	0.001643	5.44E-09	2.13E-11	-0.26779	0.631513	0.8993
FXYD2	0.000311	0.000575	3.52E-08	0.436876	-0.6634	-1.10028
FXYD5	2.43E-07	7.10E-06	3.91E-11	0.39639	-0.37776	-0.77415
FXYD6-FXYD2	4.75E-05	0.00018	1.85E-08	0.655559	-0.91809	-1.57365
FYB	0.000107	0.004364	2.42E-09	0.350377	-0.36856	-0.71893
FZD6	0.003354	0.000635	4.49E-08	0.402222	-0.58554	-0.98776
FZD7	9.58E-07	0.000858	4.97E-11	0.542185	-0.52534	-1.06753
G6PC	0.123413	0.000947	1.82E-05	-0.28286	0.756655	1.039518
GABBR1	2.41E-08	4.53E-08	1.57E-11	0.617381	-0.53988	-1.15726
GABRE	0.004031	0.00297	2.98E-07	0.462644	-0.56832	-1.03096
GABRP	0.265482	0.027147	0.000248	0.194299	-0.63381	-0.82811
GALK1	0.007896	0.001095	1.36E-08	-0.23287	0.318233	0.551104
GALNT7	1.93E-08	4.91E-08	2.68E-11	0.401609	-0.48382	-0.88543
GAPT	5.23E-05	0.001737	3.45E-09	0.233066	-0.37302	-0.60609
GAS2	0.538916	7.36E-09	1.44E-06	0.107029	0.505431	0.398401
GAS2L3	3.97E-07	1.48E-08	1.57E-11	0.430331	-0.64515	-1.07549
GBA3	0.025667	3.07E-06	2.81E-09	-0.16339	0.546549	0.709943
GBAS	9.91E-06	3.98E-05	2.79E-10	0.345818	-0.27328	-0.61909
GBP1	0.00012	0.000171	3.23E-10	0.467609	-0.68484	-1.15245
GBP2	7.94E-05	0.001517	7.70E-10	0.458092	-0.4223	-0.8804
GBP3	0.000383	1.13E-05	3.40E-10	0.699574	-0.73165	-1.43122
GBP4	0.000434	0.010538	1.58E-08	0.299664	-0.33958	-0.63925
GBP5	2.23E-06	1.29E-05	3.59E-11	0.653411	-0.85942	-1.51283
GCA	3.69E-06	1.97E-08	1.70E-11	0.412645	-0.52425	-0.9369
GCAT	0.005041	1.08E-07	1.64E-10	-0.2037	0.411057	0.614759
GCDH	0.019458	6.09E-05	7.70E-10	-0.22271	0.43908	0.661788
GCHFR	0.008665	4.49E-05	3.03E-08	-0.17667	0.354198	0.530873
GCK	0.76493	0.000121	0.000599	-0.11519	1.029606	1.144793
GCKR	0.001925	2.41E-12	2.68E-11	-0.20354	0.5933	0.796837
GDF15	0.006506	4.06E-06	2.79E-10	0.211339	-0.42688	-0.63822
GEM	1.94E-06	1.60E-06	2.68E-11	0.813498	-1.00371	-1.81721
GFRA1	0.924381	1.97E-08	2.89E-08	0.001315	0.510703	0.509389
GGACT	0.003147	3.53E-05	4.91E-09	-0.20633	0.306324	0.512657
GGTA1P	4.29E-07	0.008079	7.01E-09	0.42422	-0.3224	-0.74662

GIMAP2	6.50E-07	0.003681	9.01E-10	0.49443	-0.2725	-0.76693
GIMAP4	1.17E-05	4.70E-06	2.49E-11	0.359137	-0.43998	-0.79912
GIMAP6	2.74E-06	0.000447	3.40E-10	0.362638	-0.29547	-0.65811
GIMAP7	0.000762	0.000115	4.91E-09	0.365511	-0.42603	-0.79154
GINS1	2.14E-05	1.40E-09	1.57E-11	0.585458	-1.21422	-1.79968
GINS3	0.017581	3.66E-07	4.91E-10	0.157252	-0.48654	-0.64379
GJA1	1.56E-05	0.0009	9.01E-10	0.705782	-0.46716	-1.17294
GK	0.121853	0.012232	0.000293	0.195747	-0.31061	-0.50636
GK3P	0.271157	0.000667	1.39E-05	0.139128	-0.4643	-0.60343
GLA	1.38E-07	5.00E-09	1.85E-11	0.326052	-0.46552	-0.79157
GLIPR1	2.50E-09	5.47E-10	1.57E-11	0.434653	-0.49652	-0.93118
GLIPR2	6.77E-09	5.36E-08	1.72E-11	0.388434	-0.52873	-0.91716
GLIS2	0.005041	0.034048	6.99E-06	0.378099	-0.36311	-0.74121
GLIS3	0.000797	0.001666	6.68E-10	0.324568	-0.46331	-0.78788
GLS2	0.000838	0.000211	1.02E-07	-0.24289	0.28681	0.529698
GLT8D2	0.004939	0.014722	1.06E-06	0.218281	-0.29542	-0.5137
GLYAT	0.000797	9.20E-10	3.21E-11	-0.17718	0.604503	0.781681
GLYCTK	0.002392	2.89E-07	7.28E-11	-0.20558	0.40184	0.607416
GMFB	0.000376	1.48E-06	4.42E-10	0.269935	-0.32931	-0.59925
GMFG	8.67E-06	6.47E-07	7.28E-11	0.283663	-0.47648	-0.76014
GMNN	0.005564	2.34E-11	1.70E-11	0.153846	-0.47659	-0.63043
GMPR	0.003636	0.000947	6.56E-08	0.237602	-0.28717	-0.52477
GNE	0.007464	4.91E-10	4.19E-10	-0.12124	0.716694	0.83793
GNMT	6.84E-05	2.39E-09	1.60E-11	-0.56591	1.348963	1.914878
GNPDA1	0.000295	6.09E-05	3.97E-10	0.241341	-0.2813	-0.52264
GNPDA2	0.000208	9.17E-05	8.13E-10	0.286666	-0.29049	-0.57715
GNS	7.47E-07	7.57E-06	4.72E-11	0.287291	-0.29941	-0.5867
GOLM1	3.82E-05	3.99E-07	8.29E-11	0.38681	-0.51333	-0.90014
GOLT1B	0.002107	4.53E-08	1.64E-10	0.202802	-0.38584	-0.58864
GORAB	4.58E-06	0.002595	2.18E-09	0.481791	-0.27759	-0.75938
GPAM	0.353827	0.00159	3.37E-05	-0.08569	0.432597	0.518284
GPC3	0.024383	1.38E-06	1.96E-09	0.332435	-1.16776	-1.50019
GPC4	0.069979	0.000171	9.30E-07	0.116028	-0.52362	-0.63965
GPCPD1	1.46E-05	7.57E-06	2.43E-10	0.280669	-0.33216	-0.61283
GPER1	0.304323	4.91E-08	5.72E-10	-0.08796	0.592699	0.680655
GPNMB	1.05E-08	3.99E-07	2.43E-11	0.884681	-1.07178	-1.95646
GPR124	6.90E-07	0.001517	4.23E-09	0.369331	-0.3838	-0.75313
GPR125	0.001758	6.70E-10	8.66E-11	-0.2562	0.585778	0.841983
GPR137B	0.000159	9.17E-05	6.02E-09	0.386349	-0.4097	-0.79605
GPR137C	0.029978	9.89E-06	2.36E-08	0.14263	-0.41461	-0.55724
GPR160	1.11E-06	2.39E-09	1.82E-11	0.460972	-0.71973	-1.1807
GPR171	9.27E-07	7.85E-05	1.06E-10	0.588407	-0.66645	-1.25486
GPR18	2.56E-09	2.15E-06	2.58E-11	0.796061	-0.75298	-1.54904
GPR34	9.58E-07	1.73E-06	4.51E-11	0.57575	-0.58183	-1.15758
GPR56	0.014508	6.97E-07	1.29E-09	0.139713	-0.41304	-0.55275
GPR65	2.02E-08	1.48E-06	2.49E-11	0.639771	-0.60481	-1.24458
GPR82	0.000547	0.332437	6.11E-05	0.357236	-0.16982	-0.52706
GPR88	0.092936	0.09585	0.000176	-0.53652	0.705296	1.241814
GPRC5B	7.51E-05	1.30E-10	1.69E-11	0.150762	-0.3804	-0.53116
GPRIN3	4.11E-07	3.07E-06	2.38E-11	0.283248	-0.33536	-0.61861
GPX2	0.001643	0.000223	1.11E-09	0.590699	-0.66169	-1.25238
GPX3	0.05777	5.79E-06	3.18E-08	0.161934	-0.47093	-0.63286
GPX7	0.202778	7.48E-07	3.95E-07	0.123627	-0.78508	-0.9087
GPX8	0.003492	4.91E-08	3.48E-11	0.217668	-0.76426	-0.98193
GRAMD1B	0.003011	2.87E-06	6.34E-10	0.201682	-0.48787	-0.68955
GRAMD1C	0.273879	3.39E-07	1.94E-08	-0.09126	0.576929	0.668192

GRAMD3	0.582881	1.16E-07	5.68E-07	0.047768	0.692532	0.644763
GRB14	0.052793	2.91E-09	5.72E-10	-0.11067	0.459983	0.570653
GREM1	0.875177	2.29E-05	8.46E-05	0.036576	1.099356	1.062779
GREM2	0.600397	0.001517	0.000159	-0.07713	0.509462	0.586595
GRHL2	0.025223	0.001453	3.60E-07	0.210491	-0.42801	-0.6385
GRN	5.09E-06	3.98E-05	7.70E-10	0.313059	-0.39421	-0.70726
GRTP1	0.018194	3.53E-06	3.11E-09	-0.17093	0.404766	0.5757
GSDMB	0.184388	1.78E-08	1.06E-08	-0.09389	0.446719	0.540608
GSKIP	0.001267	4.22E-05	1.96E-09	0.256609	-0.33799	-0.5946
GSTP1	9.61E-06	4.53E-08	3.07E-11	0.319013	-0.59273	-0.91174
GUCY1A3	3.14E-06	0.013186	1.43E-08	0.338164	-0.19611	-0.53427
GYG1	3.04E-08	1.40E-09	2.78E-11	0.378322	-0.50738	-0.8857
GYPC	3.17E-05	7.10E-06	4.19E-10	0.211919	-0.31359	-0.52551
GYS2	0.080737	9.09E-09	9.05E-09	-0.06561	0.466473	0.53208
GZMA	8.43E-08	1.33E-08	1.70E-11	0.775603	-0.94287	-1.71848
GZMB	0.000232	0.000947	1.17E-07	0.409183	-0.54328	-0.95246
GZMH	0.004844	0.00052	1.41E-07	0.376226	-0.51044	-0.88666
GZMK	8.05E-09	1.02E-06	1.82E-11	0.897091	-0.79302	-1.69011
H19	0.006643	0.065616	2.58E-05	0.698016	-0.46567	-1.16368
H2AFY2	0.029467	3.99E-07	1.68E-09	0.188045	-0.56221	-0.75026
H2AFZ	1.07E-06	6.37E-08	2.58E-11	0.276872	-0.4529	-0.72977
HAAO	0.008205	2.48E-10	2.13E-11	-0.24271	0.659336	0.902049
HAGH	3.17E-05	4.52E-09	2.38E-11	-0.21366	0.402407	0.616063
HAO2	0.006779	2.89E-07	1.17E-09	-0.18315	0.784249	0.967397
HAPLN4	0.033731	7.36E-09	2.08E-10	-0.26401	0.887803	1.151809
HAUS3	0.000281	0.037344	1.17E-07	0.324808	-0.20326	-0.52807
HBA1	0.212411	0.119684	0.003152	-0.32592	0.429645	0.75556
HBA2	0.212411	0.119684	0.003152	-0.32592	0.429645	0.75556
HBB	0.135251	0.151445	0.002386	-0.27932	0.300807	0.580126
HCAR3	0.138825	0.046278	0.000231	0.221532	-0.38287	-0.6044
HCK	7.49E-09	0.001151	1.34E-10	0.563887	-0.41779	-0.98168
HCLS1	1.50E-07	6.37E-08	1.68E-11	0.450724	-0.52208	-0.9728
HCP5	1.25E-05	9.47E-07	1.82E-11	0.588293	-0.79358	-1.38188
HCST	2.85E-06	4.67E-07	7.58E-11	0.382413	-0.56912	-0.95153
HDHD3	0.257061	3.12E-08	1.17E-09	-0.07227	0.449949	0.522217
HEG1	7.27E-06	4.31E-07	1.06E-10	0.292755	-0.34407	-0.63682
HELLS	1.04E-06	1.10E-06	2.38E-11	0.311577	-0.44314	-0.75472
HENMT1	2.35E-05	8.76E-12	1.68E-11	0.240562	-0.55379	-0.79436
HEPACAM	0.307401	9.17E-05	0.003715	0.262703	0.716363	0.45366
HEPH	5.87E-05	5.73E-05	1.41E-10	0.262725	-0.36027	-0.62299
HEPN1	0.307401	9.17E-05	0.003715	0.262703	0.716363	0.45366
HES4	0.087816	2.00E-06	5.43E-09	0.13449	-0.49261	-0.6271
HGF	5.60E-07	2.31E-06	2.68E-11	0.311185	-0.39787	-0.70905
HGFAC	0.702732	4.02E-09	9.05E-09	-0.02204	0.598196	0.62024
HIF1A	2.66E-06	5.51E-07	1.68E-11	0.336177	-0.37199	-0.70816
HJURP	0.97743	9.89E-06	1.05E-05	-0.02108	-0.51042	-0.48934
HK1	1.10E-09	1.95E-09	1.57E-11	0.410057	-0.47146	-0.88152
HK3	0.000612	0.008079	6.56E-08	0.237756	-0.2873	-0.52506
HKDC1	0.006001	2.46E-07	9.63E-11	0.287952	-0.7151	-1.00305
HLA-A	1.14E-07	1.29E-05	7.95E-11	0.413285	-0.32595	-0.73924
HLA-B	8.40E-06	1.47E-05	9.12E-11	0.421983	-0.49484	-0.91682
HLA-C	4.44E-06	3.53E-05	2.79E-10	0.294871	-0.31903	-0.6139
HLA-DMA	1.22E-09	1.98E-10	1.57E-11	0.745796	-0.93316	-1.67896
HLA-	2.24E-08	6.11E-07	1.57E-11	0.560593	-0.71938	-1.27997
HLA-DPA1	6.94E-08	0.003104	6.94E-11	0.792344	-0.45786	-1.2502

HLA-DPB1	4.03E-09	2.46E-07	1.57E-11	0.470853	-0.47717	-0.94803
HLA-DPB2	0.11564	0.000495	1.11E-06	0.192137	-0.61111	-0.80325
HLA-DQA1	0.017581	0.067549	0.000112	0.56419	-0.66826	-1.23245
HLA-DQA2	1.42E-05	9.27E-06	1.56E-10	0.827782	-0.89169	-1.71947
HLA-DQB1	2.80E-05	0.00018	2.66E-10	0.556063	-0.65103	-1.20709
HLA-DRA	3.32E-08	1.08E-08	1.57E-11	0.625934	-0.76401	-1.38995
HLA-DRB1	1.10E-07	3.53E-05	1.69E-11	0.534346	-0.53703	-1.07137
HLA-DRB3	0.001137	0.165727	8.57E-06	0.498182	-0.39312	-0.89131
HLA-DRB4	8.95E-07	0.001901	2.92E-10	0.450656	-0.36672	-0.81738
HLA-DRB5	0.001603	0.184945	2.05E-05	0.46492	-0.38304	-0.84796
HLA-DRB6	0.033731	0.209631	0.000164	0.267753	-0.24199	-0.50974
HLA-E	0.000151	0.000308	4.91E-10	0.278646	-0.33603	-0.61468
HLA-F	1.28E-07	4.70E-06	1.58E-11	0.537986	-0.54873	-1.08672
HLA-G	1.67E-05	1.90E-05	3.97E-10	0.308915	-0.38429	-0.69321
HLA-J	0.001267	0.000103	7.01E-09	0.252608	-0.42085	-0.67345
HLX	0.941979	2.39E-09	3.58E-10	-0.04337	0.49018	0.533554
HMCN1	0.000778	0.0022	3.56E-09	0.42625	-0.51092	-0.93717
HMGB2	0.072174	4.16E-08	3.78E-10	0.113303	-0.39685	-0.51015
HMGCS2	0.005464	6.70E-10	3.91E-11	-0.18065	0.605783	0.786432
HMGN1	3.71E-05	1.05E-12	1.64E-11	0.160978	-0.47425	-0.63522
HMGN4	9.96E-10	4.98E-11	1.57E-11	0.509884	-0.47059	-0.98048
HMMR	0.000226	3.98E-05	1.06E-10	0.485445	-0.87195	-1.3574
HMOX1	0.118714	3.12E-05	1.21E-06	0.154601	-0.41277	-0.56737
HN1	0.000412	2.16E-11	1.57E-11	0.222733	-0.54408	-0.76682
HNF4A-AS1	0.947053	1.20E-08	4.97E-07	0.044605	0.707531	0.662926
HOGA1	0.095582	4.67E-07	4.91E-09	-0.1178	0.445114	0.562913
HOMER2	0.081872	5.44E-09	2.92E-10	-0.13639	0.605504	0.741891
HOTS	0.004466	0.088602	9.43E-05	0.377389	-0.22626	-0.60365
HPN	0.003011	1.01E-09	3.21E-11	-0.14955	0.35162	0.501169
HPR	0.002153	3.17E-10	2.38E-11	-0.16035	0.603542	0.76389
HPS5	0.10091	0.000547	4.44E-06	0.156205	-0.38037	-0.53658
HPSE	8.61E-09	1.26E-07	1.57E-11	0.541269	-0.58705	-1.12832
HRCT1	0.031057	0.003836	6.79E-07	0.200279	-0.31402	-0.5143
HS3ST2	0.149521	0.023849	3.63E-05	0.199956	-0.38405	-0.584
HSBP1L1	0.000658	9.09E-09	2.58E-11	-0.21562	0.429684	0.6453
HSD11B1	0.004844	1.62E-07	3.48E-11	-0.14599	0.419983	0.565972
HSD17B14	0.000214	8.17E-05	5.17E-09	-0.51374	0.634368	1.148106
HSPA2	2.65E-05	1.93E-07	3.59E-11	0.539348	-1.01077	-1.55012
HSPA4L	0.005464	0.00018	2.05E-07	0.431188	-0.49189	-0.92308
HSPA6	0.000838	0.000447	3.03E-08	0.231405	-0.30606	-0.53746
HSPB9	0.040115	2.03E-05	1.85E-08	-0.17582	0.523915	0.699737
HSPBAP1	2.50E-05	0.000495	1.96E-09	0.332076	-0.28188	-0.61395
HTR2B	0.003865	0.000308	1.76E-08	0.417606	-0.50045	-0.91805
IAPP	0.448609	0.000161	6.44E-06	-0.09478	0.425896	0.520674
IARS	0.053563	1.37E-07	3.28E-09	0.111241	-0.39465	-0.50589
ICAM1	1.73E-05	0.000815	1.78E-09	0.347713	-0.38058	-0.7283

ICAM2	7.94E-05	6.97E-07	1.88E-10	0.217115	-0.30216	-0.51927
ICOS	8.72E-05	3.98E-05	1.11E-09	0.260625	-0.3499	-0.61053
ID1	0.560489	0.002481	5.89E-05	0.15515	-0.57479	-0.72994
ID3	0.000838	5.44E-09	9.63E-11	0.220823	-0.6344	-0.85523
ID4	0.016925	7.10E-06	3.45E-09	0.243793	-0.66332	-0.90711
IDO1	0.001162	0.000189	3.84E-09	0.366758	-0.51092	-0.87768
IER3	0.037193	4.91E-10	7.95E-11	0.167153	-0.96309	-1.13025
IER5	3.61E-09	5.79E-06	1.64E-11	0.661535	-0.36378	-1.02531
IER5L	0.000226	0.000121	4.91E-10	0.256738	-0.2948	-0.55154
IFI16	8.17E-08	3.78E-08	2.87E-11	0.590505	-0.67494	-1.26544
IFI27	0.001359	0.026336	4.97E-07	0.551089	-0.59032	-1.14141
IFI30	1.87E-08	5.44E-09	1.57E-11	0.559829	-0.81134	-1.37117
IFI35	0.014018	2.58E-05	3.68E-08	0.174555	-0.45001	-0.62457
IFI44	7.02E-06	0.409436	2.36E-07	0.649814	-0.25064	-0.90045
IFI44L	0.000124	0.583565	1.62E-05	0.680225	-0.27161	-0.95183
IFI6	0.011462	0.055229	8.90E-07	0.60668	-0.63853	-1.24521
IFIT3	4.06E-05	0.001453	1.29E-09	0.380768	-0.4994	-0.88017
IFIT5	7.54E-06	0.004751	2.30E-09	0.32845	-0.27023	-0.59868
IFITM1	0.002153	0.056842	9.30E-07	0.319966	-0.24819	-0.56815
IFNG	0.00465	8.67E-05	5.43E-09	0.284575	-0.68415	-0.96873
IFNGR1	3.07E-05	2.43E-05	6.59E-11	0.256756	-0.3041	-0.56085
IFNGR2	1.42E-05	5.47E-10	1.57E-11	0.208841	-0.36575	-0.57459
IGFALS	0.24863	9.89E-06	1.28E-07	-0.15751	0.619659	0.777168
IGFBP6	0.000857	0.006623	3.03E-08	0.357294	-0.31416	-0.67146
IGFBP7	9.22E-05	1.40E-09	1.70E-11	0.290122	-0.65053	-0.94065
IGH	2.65E-05	0.004183	4.07E-08	0.242501	-0.31791	-0.56041
IGHA1	0.000101	0.006897	6.26E-08	0.216569	-0.28607	-0.50264
IGHA2	5.46E-06	0.002273	1.11E-08	0.324017	-0.39763	-0.72165
IGHD	0.00069	0.0009	3.03E-08	0.189311	-0.31768	-0.50699
IGHG2	5.15E-08	0.000447	1.15E-10	0.569645	-0.41537	-0.98502
IGHG3	7.05E-05	0.008079	1.23E-07	0.283238	-0.32869	-0.61193
IGHG4	9.22E-05	0.000605	1.36E-08	0.247711	-0.39442	-0.64213
IGHM	4.58E-06	0.002077	2.68E-09	0.26672	-0.3178	-0.58452
IGHV3-23	2.35E-05	0.012232	1.23E-07	0.407528	-0.43407	-0.84159
IGHV4-31	8.67E-06	0.003236	4.45E-09	0.291857	-0.32986	-0.62171
IGJ	1.55E-06	0.001151	2.79E-10	0.931708	-0.7639	-1.6956
IGK	4.18E-09	6.85E-05	2.68E-11	0.834031	-0.67498	-1.50901
IGKC	3.65E-09	6.09E-05	3.07E-11	0.764039	-0.63548	-1.39952
IGKV1-17	5.37E-09	0.004364	1.27E-10	1.368606	-0.84679	-2.21539
IGKV1-37	1.03E-08	0.000381	1.06E-10	0.945364	-0.87719	-1.82256
IGKV1D-37	1.03E-08	0.000381	1.06E-10	0.945364	-0.87719	-1.82256
IGKV1OR-1	6.03E-07	0.000575	5.45E-10	0.599582	-0.64625	-1.24583
IGKV1OR10-1	6.03E-07	0.000575	5.45E-10	0.599582	-0.64625	-1.24583
IGKV1OR2-108	4.03E-09	0.00159	4.97E-11	1.092432	-0.80425	-1.89668
IGKV1OR2-2	6.03E-07	0.000575	5.45E-10	0.599582	-0.64625	-1.24583
IGKV2-28	9.18E-09	0.000171	1.10E-10	1.107039	-0.92634	-2.03337
IGKV2D-28	9.18E-09	0.000171	1.10E-10	1.107039	-0.92634	-2.03337
IGKV4-1	1.79E-08	9.69E-05	5.17E-11	0.929474	-0.96489	-1.89437
IGLC1	1.93E-08	2.58E-05	1.10E-10	0.469505	-0.5428	-1.0123
IGLJ2	8.41E-09	3.53E-05	2.66E-10	0.659502	-0.78265	-1.44215
IGLJ3	5.72E-09	4.77E-05	1.72E-10	0.611154	-0.74689	-1.35805
IGLL3P	8.73E-09	3.29E-06	2.87E-11	0.965612	-0.87468	-1.8403

IGLL5	2.48E-06	6.20E-06	1.34E-10	0.613293	-0.95223	-1.56553
IGLV@	1.25E-08	5.73E-05	2.43E-10	0.611611	-0.74217	-1.35378
IGLV1-36	5.51E-09	2.03E-05	1.21E-10	1.185568	-1.1474	-2.33297
IGLV1-40	0.001925	0.000144	3.03E-08	0.226307	-0.61138	-0.83769
IGLV1-44	8.73E-09	3.53E-05	1.06E-10	0.567725	-0.62124	-1.18896
IGLV1-50	1.25E-08	6.09E-05	1.72E-10	0.705937	-0.87939	-1.58533
IGLV2-14	3.19E-07	0.000324	6.68E-10	0.670867	-0.87395	-1.54481
IGLV3-1	0.000159	0.001151	1.29E-08	0.265376	-0.40778	-0.67316
IGLV3-10	4.61E-05	0.002378	1.94E-08	0.50789	-0.60432	-1.11221
IGLV3-19	9.13E-08	0.000815	6.01E-10	0.659203	-0.76936	-1.42856
IGLV3-25	0.000274	0.002715	1.35E-07	0.318126	-0.41563	-0.73376
IGLV9-49	0.940703	0.018797	0.002985	0.021518	-0.48594	-0.50745
IGSF6	2.48E-06	3.07E-06	7.95E-11	0.546197	-0.75768	-1.30387
IGSF9	0.037193	0.01754	5.94E-06	-0.24838	0.345242	0.593621
IKBIP	0.000208	5.00E-09	2.43E-11	0.257372	-0.44251	-0.69988
IKZF3	0.000762	2.75E-05	2.81E-09	0.181767	-0.33539	-0.51716
IL10RA	2.51E-07	8.67E-05	5.42E-11	0.45077	-0.4212	-0.87197
IL15	1.68E-06	1.62E-07	4.51E-11	0.333574	-0.52294	-0.85652
IL15RA	0.000944	1.26E-07	1.72E-10	0.171514	-0.37339	-0.5449
IL18	9.96E-10	9.20E-10	1.57E-11	0.739487	-0.89435	-1.63384
IL1B	0.000481	4.49E-05	1.36E-08	0.17059	-0.39226	-0.56285
IL1RAP	0.188866	3.66E-07	6.26E-08	-0.15784	0.911718	1.069559
IL20RB	0.834423	0.090918	0.010929	-0.10531	0.645535	0.750841
IL21R	1.89E-05	2.15E-05	3.58E-10	0.207031	-0.42618	-0.63321
IL2RB	0.00022	0.000324	2.36E-08	0.375369	-0.41979	-0.79516
IL2RG	5.09E-06	8.17E-05	5.16E-10	0.315036	-0.38715	-0.70219
IL32	1.77E-05	1.29E-05	3.07E-11	0.861911	-0.83471	-1.69662
IL6R	0.083071	1.26E-09	1.06E-09	-0.15388	0.603148	0.757031
IL7	0.000533	2.00E-06	9.12E-11	0.329228	-0.47951	-0.80874
IL7R	3.25E-06	0.00018	1.10E-10	0.536929	-0.54271	-1.07964
INMT	0.072174	2.15E-08	1.17E-09	0.097982	-0.46608	-0.56406
INPP5F	2.43E-05	1.73E-06	6.59E-11	0.223948	-0.30312	-0.52707
IPCEF1	2.01E-05	0.000121	1.36E-09	0.325954	-0.33385	-0.6598
IQGAP1	0.00011	0.000153	1.06E-09	0.371772	-0.43333	-0.8051
IRF1	1.89E-05	2.15E-05	2.43E-11	0.467197	-0.59298	-1.06018
IRF8	5.60E-07	2.87E-06	8.29E-11	0.41711	-0.45483	-0.87194
IRS1	0.560489	4.53E-08	4.95E-08	0.038828	0.631888	0.59306
IRS2	0.399809	0.025499	0.002675	-0.15135	0.382371	0.533726
ISG15	0.000238	0.000263	4.03E-09	0.470453	-0.72413	-1.19458
ISG20	4.11E-08	8.21E-11	1.57E-11	0.592897	-1.18013	-1.77303
ISLR	0.002668	0.004364	5.20E-08	0.246465	-0.34393	-0.59039
ITGA2	0.044064	5.39E-05	7.79E-09	0.123793	-0.38681	-0.5106
ITGA4	9.13E-08	6.45E-05	1.06E-10	0.487472	-0.45745	-0.94492
ITGA6	0.012087	0.000109	1.11E-08	0.202832	-0.32476	-0.52759
ITGAL	4.11E-07	0.000858	1.79E-10	0.461832	-0.38026	-0.84209
ITGAM	5.02E-07	0.000737	3.07E-10	0.580326	-0.39565	-0.97598
ITGAV	6.41E-08	7.59E-08	1.77E-11	0.524946	-0.61524	-1.14019
ITGB2	3.83E-07	0.0002	1.15E-10	0.392624	-0.37356	-0.76618
ITGB2-AS1	0.001215	0.005625	9.52E-10	0.274451	-0.27494	-0.54939
ITGB3BP	0.007896	5.39E-06	7.22E-08	0.294194	-0.56017	-0.85436
ITGBL1	8.72E-05	5.07E-05	7.58E-11	0.380713	-0.59731	-0.97802
ITIH1	0.006001	2.51E-11	5.17E-11	-0.11964	0.509522	0.629163
ITIH5	0.000173	0.025499	2.25E-07	0.320784	-0.22008	-0.54087
ITK	3.58E-09	7.59E-08	1.68E-11	0.830897	-0.88102	-1.71192
ITLN1	0.000311	0.935938	0.00045	0.675283	0.054694	-0.62059
ITM2A	5.87E-09	6.74E-09	1.57E-11	0.905734	-0.86545	-1.77118
JAG1	3.39E-05	1.38E-06	2.43E-10	0.263735	-0.37647	-0.64021

JAK2	2.23E-06	0.012702	5.43E-09	0.308451	-0.1992	-0.50765
JAZF1	2.28E-05	0.866388	1.05E-05	0.557044	-0.0688	-0.62584
JUN	0.009684	9.89E-06	1.23E-09	0.26387	-0.47012	-0.73399
JUNB	0.005886	4.91E-08	3.78E-10	0.220092	-0.53658	-0.75667
KAL1	0.024383	1.02E-06	7.28E-11	0.152965	-0.37232	-0.52528
KANK4	0.202778	0.000121	8.52E-07	-0.20182	0.718344	0.920168
KBTBD11	0.012526	2.39E-09	2.96E-11	-0.30954	1.141827	1.451369
KBTBD8	0.000114	0.000992	7.00E-10	0.345171	-0.35283	-0.698
KCNJ10	0.000295	0.0715	1.64E-06	0.320026	-0.31441	-0.63443
KCNJ16	8.72E-05	0.003526	5.20E-08	0.415714	-0.44187	-0.85759
KCNJ2	0.000329	0.034048	5.44E-08	0.447887	-0.32173	-0.76962
KCNK5	0.254338	5.39E-05	1.58E-06	-0.18645	0.727199	0.913647
KCNN2	0.000944	2.16E-11	2.31E-11	-0.41867	2.332371	2.75104
KCNS3	0.360816	0.05377	0.002826	0.151387	-0.36284	-0.51423
KCTD12	4.11E-08	9.89E-06	1.57E-11	0.769985	-0.54835	-1.31833
KCTD9	2.28E-05	0.0007	5.45E-10	0.298603	-0.22494	-0.52355
KDELR3	0.010441	1.16E-07	1.79E-10	0.249089	-0.75504	-1.00413
KHK	0.005041	1.75E-09	3.21E-11	-0.16567	0.523729	0.689397
KIAA0101	6.10E-06	1.06E-05	8.66E-11	0.383951	-0.56701	-0.95096
KIAA0226L	4.87E-09	7.28E-05	1.85E-11	0.491706	-0.39654	-0.88825
KIAA1211	0.000742	0.000211	7.40E-09	0.196917	-0.44056	-0.63748
KIAA1598	0.000367	4.22E-05	1.29E-08	0.250207	-0.26939	-0.5196
KIAA1804	0.240437	0.002077	1.69E-05	-0.11202	0.391386	0.503408
KIF11	8.94E-05	4.70E-06	4.51E-11	0.370015	-0.79586	-1.16588
KIF14	0.023988	3.53E-05	8.59E-10	0.191846	-0.5316	-0.72344
KIF15	0.282675	2.43E-05	3.11E-07	0.083337	-0.51887	-0.60221
KIF16B	0.008995	0.000815	7.22E-08	0.236388	-0.30258	-0.53897
KIF18A	0.046617	1.47E-05	5.20E-08	0.103458	-0.42342	-0.52688
KIF18B	0.015053	0.000109	4.45E-09	0.175694	-0.3352	-0.51089
KIF20A	9.49E-05	8.71E-06	1.48E-10	0.576653	-1.03078	-1.60744
KIF21B	1.56E-05	9.89E-08	3.59E-11	0.285531	-0.38376	-0.66929
KIF2A	2.14E-06	3.53E-05	3.07E-11	0.309394	-0.2911	-0.60049
KIF4A	3.71E-05	8.07E-07	1.06E-10	0.400469	-0.71972	-1.12019
KIF5B	0.000208	1.47E-05	1.48E-10	0.225324	-0.28613	-0.51146
KLB	0.951045	5.04E-06	8.57E-06	0.073214	0.590658	0.517444
KLF6	0.000311	2.93E-05	4.19E-10	0.226139	-0.30936	-0.5355
KLHL29	0.084247	3.78E-08	1.10E-10	0.105229	-0.46751	-0.57274
KLHL6	2.50E-05	2.49E-06	3.91E-11	0.229621	-0.38001	-0.60963
KLKB1	0.001925	5.72E-11	4.51E-11	-0.16333	0.633664	0.796994
KLRB1	0.000103	0.00746	2.36E-07	0.451544	-0.36379	-0.81534
KLRC1	0.024383	0.402369	0.001462	0.301844	-0.24568	-0.54752
KLRC2	0.024383	0.402369	0.001462	0.301844	-0.24568	-0.54752
KLRC4-KLRK1	1.51E-05	2.87E-06	3.36E-11	0.472482	-0.63224	-1.10472
KLRK1	1.51E-05	2.87E-06	3.36E-11	0.472482	-0.63224	-1.10472
KNTC1	0.00012	6.66E-06	8.66E-11	0.276464	-0.50257	-0.77903
KPNA2	5.46E-06	2.26E-10	1.57E-11	0.500269	-0.8033	-1.30357
KRT18	0.001974	8.21E-11	1.70E-11	0.257593	-0.70832	-0.96592
KRT19	0.016136	4.49E-05	1.06E-08	0.244152	-0.54714	-0.79129
KRT222	0.022764	3.53E-05	4.03E-09	0.293147	-0.61409	-0.90723
KRT23	0.005249	1.08E-08	3.21E-11	0.526989	-2.92888	-3.45587
KRT8	0.000245	9.93E-09	1.10E-10	0.339665	-0.6538	-0.99346
LAIR1	0.000142	1.02E-06	1.15E-10	0.212782	-0.42546	-0.63825
LAIR2	0.114086	0.013678	2.49E-05	0.17788	-0.47206	-0.64994
LAMA2	7.54E-06	1.29E-05	1.48E-10	0.23427	-0.51234	-0.74661
LAMB1	0.003419	6.20E-06	3.63E-09	0.373887	-0.64398	-1.01787
LAMP3	9.79E-08	4.22E-05	2.68E-11	0.921365	-0.75535	-1.67672

LAPTM5	2.24E-08	5.44E-09	1.69E-11	0.527099	-0.6685	-1.1956
LAX1	0.000281	0.000136	1.00E-09	0.274599	-0.3713	-0.6459
LBH	4.03E-09	2.84E-08	1.57E-11	0.604243	-0.64914	-1.25338
LCAT	0.006252	8.31E-10	3.59E-11	-0.14581	0.433732	0.579547
LCK	2.40E-09	3.39E-07	1.58E-11	0.701199	-0.67611	-1.37731
LCN2	0.004844	3.74E-05	1.23E-09	0.26183	-0.49335	-0.75518
LCP1	1.29E-05	3.98E-05	3.59E-11	0.340703	-0.4893	-0.83001
LCP2	4.03E-09	2.15E-05	1.72E-11	0.351935	-0.33727	-0.68921
LDHB	1.10E-07	5.47E-10	1.95E-11	0.32278	-0.5551	-0.87788
LDHD	0.034876	1.61E-08	7.70E-10	-0.10893	0.41595	0.524877
LDLRAD3	0.031057	6.45E-05	3.68E-08	0.142118	-0.36821	-0.51033
LDOC1	0.004844	0.0009	4.28E-08	0.280391	-0.45347	-0.73386
LEPROTL1	2.31E-06	4.02E-09	1.95E-11	0.278893	-0.37669	-0.65558
LGALS2	7.30E-05	0.007177	6.56E-08	0.452317	-0.42513	-0.87745
LGALS3	2.94E-08	8.21E-11	1.57E-11	0.467847	-0.67359	-1.14144
LGALS3B	0.003865	2.09E-07	1.43E-09	0.345164	-0.62332	-0.96848
LGALS4	0.067013	0.000109	2.25E-07	0.458143	-1.23892	-1.69706
LGALS9	0.000571	1.29E-05	1.96E-09	0.2875	-0.42336	-0.71086
LGMN	0.000101	0.023849	2.05E-07	0.312686	-0.25102	-0.56371
LGSN	0.604702	1.12E-09	8.62E-09	-0.06031	1.254737	1.31505
LHFP	0.000658	0.029892	3.02E-06	0.321342	-0.27622	-0.59756
LHFPL2	1.21E-05	2.65E-09	2.21E-11	0.263803	-0.51191	-0.77572
LIF	0.543405	2.89E-07	5.17E-09	0.057895	-0.56156	-0.61946
LILRB1	0.000159	0.000103	1.36E-09	0.25958	-0.44238	-0.70196
LILRB2	2.43E-07	9.69E-05	4.72E-11	0.447641	-0.46353	-0.91118
LIMA1	0.000138	3.45E-08	4.72E-11	0.209698	-0.37204	-0.58174
LIME1	0.020174	2.09E-07	1.72E-10	-0.20279	0.584932	0.787725
LINC00152	0.000585	2.51E-11	2.96E-11	0.171061	-0.65668	-0.82774
LINC00844	0.029978	5.36E-08	6.34E-10	-0.16671	0.807021	0.973735
LINC00924	0.039514	0.193143	0.00392	0.242075	-0.30762	-0.5497
LINC00939	0.044713	0.001151	3.68E-08	-0.28073	0.436334	0.717061
LINC01018	0.001391	1.78E-11	2.96E-11	-0.32592	1.330447	1.656368
LINC01093	0.01561	1.26E-09	5.42E-11	-0.36875	1.451913	1.820665
LINC01094	0.07627	5.07E-05	2.36E-08	0.139126	-0.47721	-0.61633
LINC01127	0.780458	6.06E-09	2.59E-07	-0.01011	0.849129	0.859243
LINC01279	0.034291	0.011907	2.44E-06	0.323809	-0.50642	-0.83023
LINC01420	4.58E-06	1.93E-07	4.29E-11	0.275503	-0.34937	-0.62487
LIPC	0.001215	6.37E-08	1.01E-10	-0.38772	0.742835	1.130557
LIPG	0.382123	0.000293	1.39E-05	-0.07743	0.567581	0.645009
LMNB1	0.652575	0.00018	1.01E-05	0.082744	-0.46074	-0.54348
LNP1	0.011857	5.39E-06	8.13E-10	-0.19898	0.421145	0.620121
LOC100130232	0.157227	9.47E-07	4.75E-07	-0.07754	0.652845	0.730384
LOC100131541	8.40E-06	0.00297	3.63E-09	0.430455	-0.42195	-0.8524
LOC100132891	0.008827	5.39E-05	9.05E-09	0.205334	-0.41024	-0.61558

LOC100288911	0.019802	3.79E-06	1.36E-09	0.21644	-0.68115	-0.89759
LOC100288974	0.042642	1.26E-07	5.72E-10	-0.1178	0.440486	0.558291
LOC100289098	0.009684	7.28E-05	2.25E-08	-0.19642	0.342302	0.538717
LOC100505501	0.000203	7.57E-06	3.40E-10	0.304673	-0.51019	-0.81487
LOC100505812	1.46E-05	8.73E-07	4.51E-11	0.255659	-0.32418	-0.57984
LOC100505985	0.010243	1.48E-08	2.92E-10	-0.3346	1.101334	1.435931
LOC100506076	0.000412	0.004958	4.75E-07	0.421491	-0.40994	-0.83143
LOC100506098	2.35E-07	6.28E-11	1.57E-11	0.434446	-0.69947	-1.13392
LOC100506123	0.000412	0.004958	4.75E-07	0.421491	-0.40994	-0.83143
LOC100507389	0.080737	2.00E-06	3.87E-08	-0.20233	0.899585	1.101912
LOC100509445	0.063194	0.000109	2.36E-08	0.192198	-0.45944	-0.65164
LOC100509457	0.038913	0.060196	0.000335	0.590563	-0.71781	-1.30837
LOC100996756	0.307401	0.037344	0.000527	-0.16173	0.425704	0.58743
LOC100996809	9.25E-06	0.001205	1.10E-10	0.52861	-0.52596	-1.05457
LOC101060609	0.656867	1.60E-06	2.22E-05	-0.03064	0.480126	0.51077
LOC101060835	1.56E-07	1.73E-06	1.70E-11	0.645461	-0.70187	-1.34733
LOC101926960	0.004206	0.103214	1.09E-05	-0.57805	0.150144	0.728199
LOC101927180	0.000383	4.31E-07	7.00E-10	0.149779	-0.3615	-0.51128
LOC101927263	0.022024	0.004011	7.98E-07	0.243085	-0.35526	-0.59835
LOC101927287	0.056868	0.000667	6.50E-07	-0.24228	0.51368	0.755964
LOC101927331	0.011667	9.69E-05	2.25E-07	-0.32511	0.640376	0.965486
LOC101927733	2.80E-05	3.39E-12	1.57E-11	0.168293	-0.47172	-0.64001
LOC101927809	0.003492	0.00284	1.06E-08	-0.26375	0.321999	0.585754
LOC101928102	0.003956	0.020147	1.27E-06	0.279699	-0.25196	-0.53166
LOC101928104	0.001137	4.22E-05	5.17E-09	-0.32332	0.302964	0.626286
LOC101928230	0.000124	2.35E-08	1.95E-11	-0.24744	0.460692	0.708129
LOC101928274	0.003147	1.40E-09	2.31E-11	0.147606	-0.41482	-0.56243
LOC101928429	0.000778	5.39E-05	8.19E-09	0.259751	-0.58024	-0.83999
LOC101928505	0.105085	7.10E-06	0.000159	0.150074	0.525148	0.375074

LOC101928589	2.08E-06	4.52E-09	1.69E-11	0.246024	-0.39898	-0.645
LOC101928615	0.000412	0.0007	4.49E-08	0.234019	-0.26945	-0.50347
LOC101928620	3.06E-07	7.10E-06	7.28E-11	0.495367	-0.54497	-1.04034
LOC101928789	3.67E-07	5.73E-05	1.29E-09	0.369761	-0.45881	-0.82857
LOC101929143	0.001239	5.79E-06	9.52E-10	-0.2092	0.297604	0.506801
LOC101929475	0.448609	1.48E-07	2.66E-06	-0.05023	-0.51693	-0.4667
LOC101929500	0.001643	8.67E-05	2.30E-09	0.326576	-0.42974	-0.75632
LOC101929829	0.09815	0.305266	0.002826	-0.31601	0.281515	0.597521
LOC101930489	0.000585	2.51E-11	2.96E-11	0.171061	-0.65668	-0.82774
LOC102723845	0.047564	2.84E-08	4.91E-10	-0.25775	0.944068	1.201821
LOC102724156	0.008613	6.09E-05	1.02E-07	0.195703	-0.49586	-0.69157
LOC102725343	0.140554	3.39E-07	3.63E-09	-0.08946	0.42506	0.514518
LOC102725526	0.000944	0.018797	2.33E-06	0.236482	-0.26782	-0.5043
LOC145837	0.530366	0.018797	0.000618	-0.13095	0.457631	0.588583
LOC149703	0.131933	1.86E-06	2.15E-07	-0.06997	0.485354	0.555321
LOC157273	0.702732	0.000495	0.002132	0.111336	0.910568	0.799232
LOC158402	9.77E-05	1.56E-05	1.79E-10	0.24577	-0.3129	-0.55867
LOC202025	0.000422	0.114004	4.53E-07	0.346878	-0.1691	-0.51598
LOC284669	0.012307	7.02E-08	1.79E-10	-0.15987	0.358004	0.517877
LOC285181	0.752556	3.33E-05	6.19E-06	-0.02522	0.566723	0.591939
LOC285628	0.000259	0.000189	7.37E-10	0.338195	-0.50306	-0.84125
LOC374443	5.70E-05	9.05E-08	3.07E-11	0.196974	-0.32702	-0.52399
LOC389831	0.761927	0.01525	0.003519	-0.02201	0.48686	0.508873
LOC728392	9.27E-07	0.000236	8.66E-11	0.440001	-0.3502	-0.7902
LOC728715	0.063194	0.000109	2.36E-08	0.192198	-0.45944	-0.65164
LOC729680	0.125205	0.0009	4.33E-07	0.149871	-0.3769	-0.52677
LOC730101	0.137071	2.29E-05	6.26E-08	-0.17581	0.636456	0.812266
LOXL1	3.19E-07	1.38E-06	1.48E-10	0.605675	-1.26413	-1.8698
LOXL4	0.99799	6.37E-08	1.23E-09	0.022438	-0.57757	-0.60001
LPA	0.149521	1.01E-09	3.63E-09	-0.0684	0.876058	0.944462
LPAL2	0.037193	9.93E-09	1.36E-09	-0.09668	0.466841	0.563521

LPAR1	8.95E-07	2.15E-05	5.62E-11	0.233342	-0.30597	-0.53931
LPAR5	1.80E-06	3.98E-05	1.10E-10	0.266489	-0.28136	-0.54785
LPCAT1	2.94E-08	4.91E-10	1.57E-11	0.353156	-0.5207	-0.87385
LRMP	0.000252	5.39E-06	5.62E-11	0.252523	-0.36022	-0.61274
LRRC1	0.169737	8.07E-07	7.79E-09	0.105068	-0.6767	-0.78176
LRRC19	0.761927	0.028038	0.053846	0.071531	0.690561	0.619031
LRRC25	0.00101	0.00284	5.20E-08	0.232037	-0.29745	-0.52949
LRRC32	0.014508	1.02E-06	4.03E-09	0.157646	-0.44971	-0.60735
LSP1	4.75E-06	0.000211	3.11E-09	0.355268	-0.31179	-0.66706
LST1	1.89E-05	0.000342	2.95E-09	0.352364	-0.44285	-0.79521
LTBP2	0.000625	0.00199	1.85E-08	0.342265	-0.46937	-0.81164
LUM	8.95E-07	1.48E-07	1.69E-11	0.68882	-0.89454	-1.58336
LXN	8.41E-09	9.55E-12	1.57E-11	0.717318	-1.36826	-2.08558
LY75	3.97E-07	0.029892	6.01E-10	0.457446	-0.20155	-0.659
LY86	5.34E-07	1.29E-05	1.10E-10	0.426754	-0.59156	-1.01831
LY9	0.004376	1.97E-08	1.48E-10	0.162022	-0.42045	-0.58248
LY96	2.87E-08	3.53E-06	3.73E-11	0.535709	-0.60511	-1.14082
LYN	1.55E-06	3.98E-05	9.12E-11	0.361743	-0.38986	-0.75161
LYSMD2	4.90E-05	1.02E-06	4.19E-10	0.237494	-0.29752	-0.53501
LYVE1	0.002065	0.000308	4.67E-09	-0.42083	0.599232	1.02006
LYZ	5.28E-06	6.97E-07	1.15E-10	0.891342	-1.0981	-1.98945
MAB21L2	0.002778	4.53E-08	4.29E-11	0.209733	-0.52359	-0.73332
MAD2L1	6.64E-05	7.59E-08	3.48E-11	0.315932	-0.69277	-1.0087
MAFF	2.88E-05	7.59E-08	5.62E-11	0.364305	-0.73649	-1.10079
MAGI2-AS3	0.316789	1.47E-05	9.30E-07	-0.06749	0.471551	0.539042
MAMDC4	0.265482	0.000342	1.44E-06	-0.17048	0.62973	0.800209
MAML2	2.32E-08	5.07E-05	3.91E-11	0.314369	-0.26768	-0.58205
MAN2B1	1.73E-07	0.000171	7.95E-11	0.323817	-0.28314	-0.60696
MANF	0.666416	6.09E-05	0.000134	0.072218	-0.54516	-0.61738
MAOA	0.04831	7.37E-10	1.48E-10	-0.07906	0.51205	0.591105
MAP1LC3B	5.87E-05	1.26E-07	8.29E-11	0.203631	-0.31805	-0.52168
MAP2	0.003085	3.12E-05	2.04E-08	0.195327	-0.3566	-0.55193
MAP2K1	0.254338	7.36E-09	2.92E-10	-0.11145	0.656721	0.768174
MAP3K1	6.71E-07	8.67E-05	5.42E-11	0.285812	-0.25211	-0.53793
MAP3K8	1.89E-05	0.046278	1.70E-07	0.31421	-0.26618	-0.58039
MAP4K1	4.82E-07	2.58E-05	1.15E-10	0.289131	-0.37997	-0.6691
MAP7D1	9.61E-06	2.29E-05	2.54E-09	0.262535	-0.23777	-0.50031
MAPK13	0.000599	1.37E-07	5.62E-11	0.204777	-0.58782	-0.7926
MAPRE1	2.02E-08	3.17E-10	1.57E-11	0.331006	-0.46987	-0.80087
1-Mar	0.011667	1.10E-06	7.70E-10	-0.21702	0.485259	0.702279
2-Mar	9.77E-05	4.91E-10	1.60E-11	-0.25792	0.599666	0.857582
1-Mar	4.98E-09	6.20E-06	1.57E-11	0.489515	-0.48048	-0.96999
MARCKS	1.38E-08	1.12E-09	1.57E-11	0.45287	-0.53528	-0.98815
MAT1A	0.004559	5.95E-12	1.82E-11	-0.11961	0.617814	0.737422
MBL1P	0.042642	1.26E-07	5.72E-10	-0.1178	0.440486	0.558291
MBOAT1	0.000232	0.002595	3.11E-09	0.408419	-0.3169	-0.72532
MCAM	3.67E-07	0.000103	4.51E-11	0.275546	-0.30961	-0.58515
MCEE	0.003492	1.37E-07	5.72E-10	-0.1628	0.384262	0.547065
MCM10	0.024383	2.27E-07	1.17E-09	0.117985	-0.58957	-0.70755
MCM2	4.27E-08	7.37E-10	1.57E-11	0.494112	-1.00829	-1.5024
MCM3	3.14E-06	3.77E-11	1.57E-11	0.342109	-0.6931	-1.03521
MCM4	0.00567	2.84E-08	2.19E-10	0.116027	-0.39607	-0.5121
MCM5	8.15E-09	8.26E-08	1.69E-11	0.490106	-0.65928	-1.14938
MCM6	6.58E-09	9.20E-10	1.57E-11	0.429298	-0.65257	-1.08187
MCM7	0.000226	2.89E-07	5.92E-11	0.189514	-0.38968	-0.57919
MCOLN2	3.07E-05	2.75E-05	7.00E-10	0.281682	-0.35524	-0.63693

MCTP1	1.77E-05	6.85E-05	7.70E-10	0.288828	-0.37553	-0.66436
MDFIC	0.001294	4.06E-06	2.79E-10	0.194615	-0.30797	-0.50258
MDN1	0.224626	3.07E-06	1.78E-07	-0.12124	0.428722	0.549957
ME1	0.003636	1.10E-06	3.63E-09	0.244364	-0.4273	-0.67166
ME2	2.94E-06	1.93E-07	6.26E-11	0.290964	-0.35853	-0.64949
MELK	1.42E-05	1.77E-07	3.21E-11	0.508662	-1.08286	-1.59152
MEOX1	0.021277	0.000223	5.20E-08	0.178087	-0.38582	-0.5639
METRNL	6.41E-08	2.03E-05	9.12E-11	0.458918	-0.4097	-0.86862
MFAP4	3.14E-06	0.000115	6.94E-11	0.578371	-0.66812	-1.2465
MFSD1	1.19E-07	1.90E-05	5.92E-11	0.243004	-0.27164	-0.51465
MFSD6	1.35E-06	6.06E-09	1.69E-11	0.254559	-0.43613	-0.69069
MGMT	0.00101	3.53E-06	1.43E-09	-0.18784	0.320519	0.508362
MGP	3.04E-08	0.001666	9.12E-11	0.649056	-0.37998	-1.02903
MICAL1	1.85E-09	2.65E-09	1.57E-11	0.545969	-0.57875	-1.12472
MICALL1	0.000922	1.86E-06	7.00E-10	0.203791	-0.3135	-0.5173
MICB	3.36E-06	4.77E-05	3.73E-11	0.356883	-0.30938	-0.66626
MID1IP1	0.11723	0.001517	3.29E-06	0.211361	-0.4202	-0.63156
MIR155	2.44E-10	5.36E-08	1.57E-11	0.972394	-1.16422	-2.13662
MIR155HG	2.44E-10	5.36E-08	1.57E-11	0.972394	-1.16422	-2.13662
MIR1908	0.906416	0.029892	0.012631	-0.00847	-0.73118	-0.7227
MIR21	0.000164	2.16E-11	3.73E-11	0.219497	-0.58653	-0.80603
MIR224	0.004031	0.00297	2.98E-07	0.462644	-0.56832	-1.03096
MIR34A	3.44E-07	0.231418	3.63E-09	0.674417	-0.17393	-0.84835
MIR4435-1HG	0.000188	3.83E-12	1.57E-11	0.142947	-0.49093	-0.63388
MIR452	0.004031	0.00297	2.98E-07	0.462644	-0.56832	-1.03096
MIR6734	0.000169	2.16E-11	2.43E-11	0.200547	-0.41529	-0.61583
MIR675	0.006643	0.065616	2.58E-05	0.698016	-0.46567	-1.16368
MIR6756	4.46E-07	0.000495	8.29E-11	0.260159	-0.25512	-0.51528
MIR6778	0.006001	1.38E-05	4.23E-09	-0.19499	0.372973	0.567963
MIR7703	0.003419	9.27E-06	1.72E-10	0.188252	-0.3696	-0.55785
MIR8071-1	5.98E-08	0.00018	7.58E-11	0.875599	-0.44868	-1.32428
MIR8071-2	5.98E-08	0.00018	7.58E-11	0.875599	-0.44868	-1.32428
MKI67	0.067013	9.05E-08	1.96E-09	0.076928	-0.49492	-0.57185
MLKL	2.55E-06	7.38E-11	1.57E-11	0.316597	-0.47458	-0.79118
MLLT11	0.030526	2.43E-05	3.52E-08	0.262255	-0.79351	-1.05576
MLPH	0.217268	1.56E-05	5.94E-07	-0.09439	0.410824	0.505217
MLXIPL	0.06045	6.74E-09	1.23E-08	-0.1065	0.576067	0.682571
MMAB	0.017877	0.000211	1.06E-06	-0.17119	0.334507	0.505694
MMD	0.016372	5.51E-07	7.70E-10	0.108844	-0.39994	-0.50878
MME	0.144149	1.77E-07	1.72E-10	-0.24007	0.84518	1.085247
MMGT1	8.20E-05	2.46E-07	8.66E-11	0.283949	-0.27108	-0.55503
MMP7	2.97E-05	7.36E-09	1.91E-11	0.523928	-1.19038	-1.7143
MMP9	0.006907	0.036235	7.90E-06	0.324517	-0.40158	-0.7261
MND1	0.003492	1.08E-08	2.87E-11	0.291072	-1.13023	-1.4213
MNDA	2.51E-07	1.06E-05	1.70E-11	0.598407	-0.58349	-1.1819
MNS1	0.003419	3.53E-10	3.21E-11	0.26404	-0.91293	-1.17697
MOB1A	6.78E-06	0.000775	4.91E-10	0.319215	-0.29333	-0.61254
MOGAT1	0.011462	0.000189	4.12E-07	-0.28068	0.541939	0.82262
MOGAT2	0.047564	6.70E-10	8.66E-11	-0.10747	0.473725	0.581196
MORC4	0.002153	4.97E-12	1.57E-11	0.265799	-0.99835	-1.26415
MOXD1	0.008506	2.87E-06	5.73E-09	0.12719	-0.59736	-0.72455
MPDZ	0.008205	4.97E-12	1.56E-10	-0.14479	0.542291	0.687077
MPEG1	4.56E-08	1.47E-05	2.58E-11	0.501038	-0.43718	-0.93822
MPND	0.009337	1.60E-06	1.59E-09	-0.1756	0.380131	0.555735

MPPED1	0.000383	0.001517	2.25E-08	-0.31479	0.281898	0.59669
MPV17	2.31E-06	2.18E-09	2.38E-11	0.245521	-0.45162	-0.69714
MR1	5.15E-08	9.89E-06	1.64E-11	0.2965	-0.23857	-0.53507
MROH2A	0.004122	0.000121	2.07E-09	-0.8723	1.150884	2.023183
MS4A1	4.58E-06	5.39E-06	2.31E-10	0.41478	-0.59034	-1.00512
MS4A4A	2.74E-05	3.07E-06	5.92E-11	0.305032	-0.42277	-0.7278
MS4A7	6.06E-05	3.12E-05	1.48E-10	0.422796	-0.54525	-0.96805
MSH2	4.58E-06	5.10E-07	5.62E-11	0.455024	-0.47555	-0.93058
MSR1	2.74E-06	0.000381	1.99E-10	0.308918	-0.28226	-0.59118
MT1F	0.033144	0.00284	6.50E-07	-0.23393	0.459886	0.693819
MT1G	0.041384	0.00018	1.11E-08	-0.24946	0.498938	0.748397
MT1H	0.037783	0.002077	1.71E-06	-0.1735	0.334328	0.507827
MT1M	0.019802	0.028038	6.44E-06	-0.51351	0.684419	1.197928
MT1X	0.017877	6.85E-05	3.18E-08	-0.16696	0.412651	0.579616
MTCL1	0.000124	1.38E-05	2.92E-10	0.435311	-0.6419	-1.07721
MTHFD2	8.36E-07	2.39E-09	2.78E-11	0.374754	-0.57041	-0.94516
MTMR11	0.004122	0.004364	5.43E-09	0.237663	-0.2877	-0.52536
MTMR2	2.55E-06	3.66E-07	2.19E-10	0.225172	-0.33304	-0.55822
MUM1L1	0.040115	0.001095	1.32E-06	0.296482	-0.62323	-0.91971
MVP	1.46E-05	1.20E-08	4.51E-11	0.371304	-0.60786	-0.97916
MX1	0.000156	0.047733	3.11E-07	0.58376	-0.56232	-1.14608
MX2	2.80E-05	0.105713	3.18E-08	0.516508	-0.37054	-0.88705
MXI1	0.000778	9.20E-10	5.62E-11	-0.23421	0.495388	0.729601
MXRA5	0.000193	0.019452	7.40E-09	0.387312	-0.26608	-0.65339
MYC	1.68E-07	2.16E-11	1.57E-11	0.528914	-0.99828	-1.5272
MYL12B	2.58E-05	2.41E-12	2.43E-11	0.137327	-0.37162	-0.50894
MYO16	0.301136	1.77E-07	3.63E-09	-0.11284	0.563928	0.676766
MYO1F	1.23E-09	0.000189	2.21E-11	0.445082	-0.29981	-0.74489
MYO1G	1.67E-05	3.07E-06	2.96E-11	0.293614	-0.39978	-0.6934
MYO5A	4.42E-09	5.51E-07	1.58E-11	0.293145	-0.31417	-0.60732
MYOF	9.13E-08	1.29E-05	4.29E-11	0.381691	-0.34815	-0.72984
MYOM1	0.052793	0.446815	0.005378	0.411554	-0.18184	-0.5934
MZB1	1.94E-06	7.48E-07	1.27E-10	0.489975	-0.87871	-1.36868
NAA40	2.02E-07	4.70E-06	7.58E-11	0.286492	-0.26794	-0.55443
NABP1	2.55E-06	1.86E-06	1.57E-11	0.368451	-0.43563	-0.80408
NAGK	1.17E-05	1.06E-05	2.95E-09	0.253309	-0.37453	-0.62784
NAGS	0.336738	1.26E-07	5.44E-08	-0.08785	0.594401	0.682248
NALCN	0.000329	4.77E-05	3.58E-10	0.219847	-0.42653	-0.64637
NAP1L1	1.44E-08	1.08E-08	1.58E-11	0.346014	-0.36304	-0.70905
NAP1L3	0.006779	0.055393	3.01E-05	0.367798	-0.27213	-0.63993
NAPSB	0.000138	0.162	2.05E-05	0.448344	-0.19114	-0.63948
NARR	7.54E-08	3.77E-11	1.57E-11	0.434585	-0.69464	-1.12923
NBEA	0.012087	0.009067	2.85E-07	0.272249	-0.35255	-0.6248
NCAPD2	0.040743	0.001095	4.12E-07	0.203934	-0.31071	-0.51464
NCAPG	0.000571	1.93E-07	4.10E-11	0.346259	-1.02029	-1.36655
NCEH1	1.93E-08	1.90E-05	2.13E-11	0.837006	-0.66772	-1.50472
NCF1	4.46E-07	8.71E-06	5.42E-11	0.475259	-0.50324	-0.9785
NCF1B	6.90E-07	3.12E-05	7.95E-11	0.470598	-0.47887	-0.94947
NCF1C	6.90E-07	3.12E-05	7.95E-11	0.470598	-0.47887	-0.94947
NCF2	3.55E-07	1.18E-06	7.58E-11	0.488201	-0.71039	-1.19859
NCF4	3.69E-06	5.73E-05	2.08E-10	0.366157	-0.43258	-0.79874
NCK2	4.29E-06	0.000775	4.03E-09	0.3752	-0.40342	-0.77862
NCKAP1L	4.98E-09	3.98E-05	2.87E-11	0.425062	-0.38801	-0.81307
NDC80	1.60E-07	2.09E-07	1.77E-11	0.767343	-1.12022	-1.88756
NDN	0.000311	0.000277	1.58E-08	0.288329	-0.39302	-0.68135
NEB	0.016372	0.000263	2.42E-09	0.379322	-0.54113	-0.92045
NEDD9	6.44E-05	4.33E-11	1.57E-11	0.162132	-0.34694	-0.50907
NELL2	0.003289	0.000401	1.59E-09	0.305869	-0.48249	-0.78836

NETO2	0.033144	4.91E-08	9.01E-10	0.119625	-0.43052	-0.55014
NEU4	0.014761	1.78E-05	4.71E-08	-0.16551	0.413188	0.578696
NEURL1B	8.15E-09	5.73E-05	5.62E-11	0.52306	-0.34777	-0.87083
NEURL3	0.009798	7.57E-06	1.59E-09	0.192595	-0.38445	-0.57704
NEXN	0.00225	7.74E-05	2.92E-10	0.411634	-0.58463	-0.99627
NFE2L3	2.50E-05	4.02E-09	1.57E-11	0.212379	-0.52745	-0.73983
NFKBIE	1.94E-07	2.67E-07	1.91E-11	0.376814	-0.49419	-0.87101
NFKBIZ	0.437549	0.000171	2.68E-05	-0.09451	0.467732	0.562243
NKG7	6.78E-06	0.000263	1.96E-09	0.507165	-0.55957	-1.06674
NLRC3	3.55E-07	4.55E-06	6.94E-11	0.485902	-0.5003	-0.9862
NLRC5	1.25E-06	7.57E-06	3.48E-11	0.650231	-0.6287	-1.27894
NME5	0.133573	0.000153	5.03E-06	0.142758	-0.57178	-0.71454
NMI	0.015305	0.000115	4.45E-09	0.170021	-0.34897	-0.51899
NMRK1	0.329836	6.28E-11	7.28E-11	-0.0586	0.465637	0.524237
NOD2	9.58E-07	2.29E-05	1.56E-10	0.445088	-0.43172	-0.87681
NOL4	0.326583	0.007177	0.00017	-0.12533	0.377099	0.502431
NPC2	2.48E-06	3.14E-07	8.66E-11	0.27937	-0.43745	-0.71682
NPNT	0.011462	0.000858	6.50E-07	0.173757	-0.46374	-0.6375
NPTX2	0.091674	0.002175	6.79E-07	0.178336	-0.45103	-0.62936
NPW	0.003572	0.006364	5.20E-07	0.715169	-0.6716	-1.38677
NPY6R	0.191062	0.006112	0.030142	0.175793	0.507702	0.331909
NQO1	0.50179	5.07E-05	1.11E-06	0.05418	-0.8897	-0.94388
NR1I2	0.003956	3.12E-08	2.66E-10	-0.17751	0.518621	0.696133
NR1I3	0.023556	1.73E-10	1.01E-10	-0.14465	0.631048	0.775703
NR3C2	0.177862	1.16E-10	1.06E-09	-0.04702	0.539387	0.586409
NRROS	3.84E-06	0.014722	9.05E-09	0.288598	-0.21838	-0.50698
NSUN6	0.522219	1.28E-06	7.90E-06	-0.01846	0.533721	0.552184
NT5C3A	0.00112	0.000114	1.11E-09	0.212277	-0.34652	-0.55879
NTHL1	0.003419	6.66E-06	6.02E-09	-0.20397	0.340416	0.544387
NTS	0.003085	1.29E-05	2.19E-10	0.624708	-1.31453	-1.93923
NUAK2	9.25E-06	0.000362	2.08E-10	0.42888	-0.35847	-0.78735
NUF2	0.005564	2.49E-06	5.17E-11	0.264887	-0.66878	-0.93367
NUPR1	0.056868	7.36E-09	3.58E-10	-0.16016	0.432371	0.592528
NUSAP1	9.25E-06	0.000189	3.21E-11	0.559315	-0.70847	-1.26778
OAS1	0.028997	0.177315	0.000284	0.267705	-0.29622	-0.56393
OAS2	0.000383	0.000635	1.11E-09	0.358974	-0.54219	-0.90116
OAT	0.191062	0.006623	4.05E-05	-0.16634	0.544368	0.710704
ODC1	0.292008	3.77E-11	1.01E-10	0.092806	-0.77209	-0.8649
OGFRL1	4.03E-09	1.18E-06	1.91E-11	0.424279	-0.31947	-0.74375
OIP5	0.001722	1.48E-06	1.64E-10	0.330441	-0.82879	-1.15923
OLFML1	0.000117	0.001517	5.73E-09	0.392006	-0.32693	-0.71894
OLFML2B	0.007314	0.000109	2.75E-08	0.184881	-0.38656	-0.57144
OLFML3	3.49E-05	0.015804	2.75E-08	0.392041	-0.21109	-0.60313
ORC6	0.005041	1.16E-07	3.07E-10	0.152972	-0.59652	-0.7495
OSBPL3	2.44E-10	1.12E-09	2.04E-11	0.615279	-0.69474	-1.31002
OSBPL6	0.639418	1.13E-05	5.44E-07	-0.04303	0.483208	0.526239
OSCAR	0.009684	4.06E-06	3.11E-09	0.175712	-0.41219	-0.5879
OSMR	7.51E-05	3.12E-05	2.56E-10	0.308745	-0.4064	-0.71515
OTUD6B	0.003085	0.001453	5.44E-08	0.298814	-0.32876	-0.62757
OVGP1	0.029978	7.73E-07	1.27E-10	-0.1434	0.551641	0.695043
OVOS	0.063194	0.000109	2.36E-08	0.192198	-0.45944	-0.65164
OVOS2	0.063194	0.000109	2.36E-08	0.192198	-0.45944	-0.65164
OXCT1	8.95E-08	6.47E-07	2.38E-11	0.496076	-0.60537	-1.10145
P2RX5	1.13E-05	4.33E-11	1.57E-11	0.28777	-0.54378	-0.83155
P2RX5-TAX1BP3	2.21E-05	6.28E-11	1.69E-11	0.384727	-0.73882	-1.12355
P2RY12	0.005359	0.061959	1.23E-05	0.313061	-0.30005	-0.61312
P2RY13	2.94E-06	0.00297	1.11E-09	0.546128	-0.39679	-0.94292

P2RY14	1.77E-05	0.000635	1.36E-09	0.413975	-0.44557	-0.85955
P2RY8	2.74E-06	2.84E-08	1.60E-11	0.40971	-0.65692	-1.06663
PACSIN3	0.001326	6.97E-07	4.67E-10	-0.29557	0.58704	0.882615
PAG1	1.22E-09	4.91E-08	1.57E-11	0.58472	-0.47216	-1.05688
PAIP2B	0.010046	1.26E-09	4.29E-11	-0.18457	0.617322	0.801887
PALLD	0.001603	2.39E-09	8.29E-11	0.229743	-0.5557	-0.78545
PALM2	0.227098	2.31E-06	1.62E-07	-0.07025	0.481262	0.551517
PALM3	0.099526	8.17E-05	1.21E-06	-0.10013	0.42798	0.528105
PAM	0.000142	1.13E-05	3.40E-10	0.248151	-0.30613	-0.55429
PAN2	0.042025	2.89E-07	2.14E-08	-0.10674	0.418178	0.524919
PANK1	0.289029	3.66E-07	3.43E-07	-0.0757	0.471527	0.547224
PAPLN	2.09E-07	4.49E-05	5.17E-11	0.370002	-0.39952	-0.76952
PAPSS1	0.019115	5.39E-06	8.19E-09	0.174306	-0.33899	-0.51329
PAQR5	0.072174	0.000605	1.28E-07	0.147446	-0.39287	-0.54031
PAQR8	1.23E-09	9.89E-08	1.57E-11	0.473823	-0.55364	-1.02746
PARP12	6.71E-07	3.98E-05	5.72E-10	0.347654	-0.40072	-0.74837
PARP8	4.03E-09	2.35E-08	1.57E-11	0.459384	-0.44209	-0.90147
PARVG	2.74E-05	5.10E-07	7.95E-11	0.197252	-0.33552	-0.53278
PBK	4.18E-05	3.29E-06	3.48E-11	0.854471	-1.3762	-2.23067
PBLD	0.002021	1.10E-06	7.00E-10	-0.19828	0.427507	0.625787
PCBD1	0.000965	1.48E-07	8.29E-11	-0.27457	0.467228	0.741797
PCDH17	0.002344	0.000858	2.89E-08	0.250575	-0.41257	-0.66315
PCED1B	3.55E-07	2.89E-07	2.96E-11	0.416473	-0.46546	-0.88193
PCK1	0.157227	0.000362	2.79E-05	-0.30039	0.950871	1.25126
PCNA	3.04E-06	2.26E-10	1.57E-11	0.346196	-0.66459	-1.01078
PCOLCE2	0.000238	9.89E-08	2.19E-10	-0.59365	1.574618	2.168272
PCYOX1L	0.000214	5.97E-07	4.29E-11	0.283077	-0.45507	-0.73815
PDGFA	0.000585	1.37E-07	2.04E-11	0.289954	-0.59517	-0.88513
PDGFD	6.26E-07	0.001737	2.81E-09	0.476145	-0.42603	-0.90218
PDK4	0.19349	8.71E-06	4.28E-08	-0.20387	0.945017	1.148885
PDP1	8.95E-07	4.37E-06	5.92E-11	0.315668	-0.38709	-0.70276
PDZK1IP1	0.008827	1.66E-05	3.97E-10	0.33436	-1.08367	-1.41803
PDZRN3	1.15E-06	0.008401	4.23E-09	0.278888	-0.24108	-0.51997
PEA15	2.88E-05	1.33E-08	3.48E-11	0.279622	-0.40891	-0.68853
PECAM1	7.49E-09	1.40E-09	1.57E-11	0.271087	-0.33986	-0.61094
PEG10	0.086703	0.090918	0.000657	0.269685	-0.59962	-0.86931
PFKFB3	0.000159	0.010145	2.54E-09	0.586749	-0.3958	-0.98255
PFKP	1.77E-05	5.36E-08	3.91E-11	0.19669	-0.40373	-0.60042
PFN2	0.042642	0.311163	0.000313	-0.33069	0.211388	0.542082
PHF19	2.72E-07	1.01E-09	1.95E-11	0.231936	-0.4637	-0.69563
PHLDA1	2.74E-06	5.97E-07	2.79E-10	0.405819	-0.59513	-1.00095
PHLDA2	0.007751	8.21E-11	5.62E-11	0.101005	-0.48733	-0.58833
PHYHD1	0.023164	8.07E-07	5.62E-11	-0.17329	0.427	0.600289
PIGR	0.000338	3.33E-05	2.66E-10	0.257145	-0.34684	-0.60399
PIK3C2G	0.032081	1.48E-06	1.17E-07	-0.13306	0.450937	0.583999
PIK3CG	3.61E-09	5.97E-07	2.04E-11	0.273031	-0.28012	-0.55315
PIK3R3	1.95E-05	1.38E-06	1.41E-10	0.233788	-0.27069	-0.50448
PILRA	6.03E-09	8.17E-05	3.73E-11	0.40209	-0.4144	-0.81649
PIM2	2.65E-05	2.93E-05	1.48E-10	0.238115	-0.3037	-0.54182
PIR	0.064163	0.000171	2.47E-07	0.185449	-0.3765	-0.56195
PIWIL4	2.82E-07	0.000277	6.34E-10	0.355111	-0.48151	-0.83662
PJA1	0.003786	3.14E-07	4.19E-10	0.188061	-0.35007	-0.53813
PKDCC	0.002837	9.17E-05	5.73E-09	0.209416	-0.39207	-0.60148
PKIB	0.131933	0.000189	9.21E-08	0.22876	-0.69052	-0.91928
PKLR	0.001531	2.27E-07	3.58E-10	-0.26474	0.51567	0.780406
PKM	0.000238	6.37E-08	1.21E-10	0.199961	-0.39003	-0.58999
PLA2G2A	0.240437	0.003104	0.000121	0.347031	-1.02841	-1.37544
PLA2G4A	1.87E-07	5.86E-08	1.95E-11	0.474133	-0.57456	-1.0487

PLA2G7	0.00011	1.56E-05	2.19E-10	0.584558	-0.88917	-1.47373
PLAC8	0.000259	0.173471	7.90E-06	0.318147	-0.21243	-0.53058
PLAGL1	6.58E-09	2.00E-06	3.91E-11	0.544735	-0.45939	-1.00412
PLAT	0.025223	0.332437	0.000846	0.318318	-0.18555	-0.50387
PLAUR	0.000612	5.47E-10	3.91E-11	0.128622	-0.39723	-0.52585
PLBD1	2.87E-08	2.67E-07	3.21E-11	0.514621	-0.63543	-1.15005
PLCXD3	1.25E-06	0.000136	5.72E-10	0.503677	-0.72799	-1.23166
PLEK	1.33E-07	0.001264	3.97E-10	0.466634	-0.47588	-0.94252
PLEKHO1	8.36E-07	6.37E-08	1.57E-11	0.396393	-0.5382	-0.93459
PLEKHO2	0.000142	6.47E-07	5.16E-10	0.214892	-0.30948	-0.52437
PLG	0.008345	3.93E-10	6.01E-10	-0.06604	0.485207	0.551244
PLIN1	0.99799	0.000103	7.32E-05	-0.00637	0.699295	0.705666
PLIN3	0.000142	1.78E-08	6.59E-11	0.223648	-0.37198	-0.59562
PLK2	8.36E-07	2.58E-05	6.94E-11	0.469026	-0.31844	-0.78747
PLP2	1.03E-08	5.44E-09	1.57E-11	0.501337	-0.6612	-1.16253
PLSCR1	0.000198	1.28E-06	1.27E-10	0.18327	-0.32096	-0.50424
PLTP	1.51E-05	3.74E-05	3.97E-10	0.417787	-0.4722	-0.88999
PLXDC2	6.41E-08	1.37E-07	1.72E-11	0.31682	-0.39716	-0.71398
PLXNC1	1.38E-07	7.59E-08	1.85E-11	0.259396	-0.25967	-0.51907
PMEPA1	3.82E-05	2.93E-05	1.56E-10	0.421179	-0.61277	-1.03395
PNMA1	8.73E-09	1.30E-10	1.57E-11	0.461187	-0.59305	-1.05423
PNMA2	0.000103	2.31E-06	2.78E-11	0.238068	-0.34385	-0.58191
PNMAL1	0.001137	0.001322	1.96E-09	0.325631	-0.35779	-0.68342
PODXL	1.38E-05	0.012232	4.95E-08	0.448653	-0.33445	-0.7831
POLA1	0.000658	0.00159	7.22E-08	0.245006	-0.26038	-0.50538
POLE2	0.039514	0.009067	4.88E-05	0.235917	-0.33904	-0.57496
PON1	0.123413	2.27E-07	1.00E-09	-0.10225	0.458018	0.560272
POU2AF1	3.06E-07	7.10E-06	7.28E-11	0.495367	-0.54497	-1.04034
PPA1	0.000259	3.78E-08	1.57E-11	0.194643	-0.48219	-0.67683
PPAP2C	8.99E-06	0.000381	4.19E-10	0.545004	-0.70422	-1.24922
PPAPDC1A	0.001215	7.28E-05	6.53E-09	0.378845	-0.71357	-1.09241
PPBP	0.227098	0.011798	2.90E-05	-0.16307	0.392941	0.556008
PPDPF	0.600397	1.06E-05	2.14E-05	0.047584	-0.47264	-0.52023
PPIH	0.003492	3.45E-08	9.01E-10	0.153879	-0.3497	-0.50358
PPM1E	0.153348	1.47E-05	2.04E-08	-0.16711	0.448564	0.615674
PPM1M	1.33E-08	5.07E-05	9.12E-11	0.301317	-0.2816	-0.58292
PPP1R12	1.13E-05	5.04E-06	6.68E-10	0.25327	-0.25475	-0.50802
PPP1R14	0.105085	0.001043	2.77E-06	0.17273	-0.41288	-0.58561
PPP1R16B	1.94E-06	4.06E-06	3.48E-11	0.329955	-0.40995	-0.7399
PPP1R18	1.60E-07	2.49E-06	2.21E-11	0.374151	-0.42219	-0.79634
PPP1R1A	0.000817	2.18E-09	2.78E-11	-0.42719	1.485073	1.912259
PPP1R3C	0.556426	8.73E-07	1.38E-06	-0.05161	0.879218	0.930828
PPP4R1	6.03E-07	3.78E-08	1.72E-11	0.285938	-0.33285	-0.61879
PPT1	6.54E-09	8.26E-08	1.57E-11	0.46482	-0.50843	-0.97325
PRC1	9.22E-05	4.31E-07	1.95E-11	0.664039	-1.12114	-1.78517
PREX1	2.08E-08	2.31E-06	6.26E-11	0.37455	-0.3647	-0.73925
PRF1	8.67E-06	0.000249	5.43E-09	0.301626	-0.3626	-0.66422
PRG4	0.775528	3.79E-06	5.46E-05	0.078241	0.59602	0.517779
PRKAR2B	6.55E-06	0.029892	5.97E-08	0.414654	-0.16207	-0.57672
PRKCD	2.01E-05	0.000263	6.02E-09	0.300781	-0.33559	-0.63637
PRKX	1.74E-09	5.39E-06	2.78E-11	0.461148	-0.40463	-0.86578
PRKY	0.002837	0.011356	4.33E-07	0.249929	-0.25743	-0.50736
PROCR	4.75E-06	0.001264	1.10E-10	0.468201	-0.45192	-0.92012
PROM1	5.70E-05	0.00199	5.44E-08	0.665986	-0.68923	-1.35521
PROX1	0.587238	6.85E-05	8.23E-06	-0.03077	0.601243	0.632014
PROZ	0.807852	0.018797	0.001644	-0.05946	0.44624	0.505702
PRR15L	0.003865	0.050673	7.60E-05	0.261335	-0.33461	-0.59595

PRSS23	0.047564	2.46E-07	1.17E-09	0.119137	-0.38908	-0.50822
PRTFDC1	0.001215	2.68E-06	1.21E-10	0.382068	-0.62344	-1.00551
PSMB10	0.002498	1.21E-05	3.07E-10	0.264651	-0.45599	-0.72064
PSMB8	0.004376	2.93E-05	4.67E-10	0.299353	-0.528	-0.82735
PSMB9	3.39E-05	5.79E-06	5.17E-11	0.531068	-0.79981	-1.33087
PSMD14	0.030526	4.02E-09	4.97E-11	0.128404	-0.402	-0.53041
PSME2	0.003419	9.27E-06	1.72E-10	0.188252	-0.3696	-0.55785
PSTPIP1	6.25E-05	1.21E-05	4.97E-11	0.315709	-0.39763	-0.71334
PSTPIP2	0.000625	0.019452	9.67E-08	0.332918	-0.24783	-0.58075
PTGDS	3.36E-06	0.000575	2.19E-10	0.786044	-0.78315	-1.56919
PTGER2	0.000422	0.021535	3.03E-08	0.312639	-0.30822	-0.62086
PTGER4	5.60E-07	1.38E-05	4.97E-11	0.404619	-0.38134	-0.78595
PTGFRN	5.23E-05	0.000136	1.96E-09	0.309883	-0.37105	-0.68093
PTPN22	1.94E-07	3.29E-06	2.49E-11	0.24911	-0.33896	-0.58807
PTPRC	5.36E-08	5.07E-05	3.07E-11	0.522913	-0.4388	-0.96172
PTPRCAP	4.33E-05	2.89E-07	3.59E-11	0.303821	-0.50337	-0.80719
PTTG1	0.000311	4.53E-08	8.29E-11	0.413488	-0.87558	-1.28906
PVRIG	4.82E-07	2.15E-08	1.77E-11	0.464671	-0.68573	-1.1504
PXDN	0.000208	3.79E-06	2.79E-10	0.303446	-0.37066	-0.67411
PXMP2	5.70E-05	1.08E-08	5.42E-11	-0.20773	0.423153	0.630882
PYCARD	9.13E-08	2.16E-11	1.57E-11	0.419916	-0.65777	-1.07769
PYHIN1	2.09E-07	0.001205	3.07E-10	0.477812	-0.38755	-0.86536
PZP	0.411112	0.0009	0.002255	0.283964	1.406878	1.122913
QPCT	0.000159	7.59E-08	2.31E-11	0.339086	-0.75057	-1.08965
RAB11B-AS1	0.111029	0.000136	4.53E-07	-0.16643	0.385801	0.55223
RAB25	0.050444	1.56E-05	7.70E-10	0.461479	-1.49453	-1.95601
RAB31	3.61E-09	7.37E-10	1.57E-11	0.467593	-0.51428	-0.98187
RAB32	0.053563	4.06E-06	4.45E-09	0.107308	-0.44325	-0.55056
RAB34	7.54E-08	3.77E-11	1.57E-11	0.434585	-0.69464	-1.12923
RAB38	0.011667	1.47E-05	5.16E-10	0.2201	-0.53999	-0.76009
RAB8B	1.33E-08	3.07E-06	1.91E-11	0.387493	-0.38861	-0.7761
RAC2	1.45E-06	1.02E-06	3.59E-11	0.369094	-0.55699	-0.92608
RACGAP1	2.43E-07	2.31E-06	3.91E-11	0.59393	-0.75896	-1.35289
RAD51AP1	3.44E-07	1.10E-06	1.77E-11	0.514997	-0.83172	-1.34672
RAI2	0.042642	0.006897	7.29E-06	0.197679	-0.30423	-0.50191
RALGDS	5.37E-05	1.62E-07	4.19E-10	0.22433	-0.32271	-0.54704
RAMP1	0.209969	0.001322	3.76E-05	-0.10818	0.454598	0.56278
RANBP3L	0.477079	1.01E-09	5.43E-09	-0.06827	0.47519	0.543458
RAPH1	0.793757	9.69E-05	0.000149	0.052679	0.523126	0.470447
RARRES1	6.70E-08	4.02E-09	1.57E-11	0.649265	-1.01172	-1.66098
RARRES3	0.068961	0.001816	3.77E-07	0.202885	-0.4722	-0.67509
RASGEF1A	1.38E-07	0.000189	3.73E-11	0.293209	-0.2243	-0.51751
RASGRP1	3.61E-09	1.77E-07	1.57E-11	0.9981	-0.86037	-1.85847
RASSF2	6.52E-09	2.68E-06	2.58E-11	0.59764	-0.4457	-1.04334
RASSF3	3.58E-06	7.37E-10	1.72E-11	0.452238	-0.6046	-1.05684
RBBP8	0.001531	8.76E-12	1.58E-11	0.221503	-0.56739	-0.78889
RBM3	0.000164	9.89E-06	2.66E-10	0.308782	-0.34562	-0.6544
RBP5	0.001758	2.39E-09	5.17E-11	-0.23315	0.580955	0.814104
RCAN2	1.74E-06	0.001901	4.03E-09	0.859242	-0.60673	-1.46598
RCAN3	2.01E-05	3.66E-07	2.43E-11	0.234601	-0.3822	-0.6168
RCC2	6.71E-07	4.98E-11	2.31E-11	0.282899	-0.46796	-0.75086
RCL1	0.002896	2.68E-06	1.23E-09	-0.25838	0.352518	0.610901
RCN2	0.002778	3.93E-10	2.31E-11	0.1691	-0.41403	-0.58313
RCSD1	2.08E-08	7.10E-06	4.10E-11	0.526237	-0.51026	-1.0365
RDH12	0.175754	0.014722	3.37E-05	0.230198	-0.49648	-0.72668

RDH16	0.131933	3.39E-07	3.68E-08	-0.10728	0.521173	0.628449
RECQL	2.28E-05	7.59E-08	2.21E-11	0.322529	-0.39131	-0.71384
RELB	0.003289	0.000575	9.21E-08	0.235466	-0.38555	-0.62101
RERG	9.91E-06	0.037344	5.20E-08	0.343371	-0.28431	-0.62768
RFC4	8.94E-05	1.33E-08	2.68E-11	0.308113	-0.59737	-0.90548
RFX5	9.13E-08	2.34E-11	1.57E-11	0.338029	-0.56291	-0.90094
RGCC	1.99E-08	3.53E-06	5.42E-11	0.431283	-0.43314	-0.86442
RGL1	2.74E-06	0.000495	6.01E-10	0.314734	-0.19524	-0.50998
RGS1	9.79E-08	1.93E-07	1.34E-10	0.376661	-0.49665	-0.87331
RGS10	3.61E-09	3.24E-09	1.57E-11	0.336961	-0.44973	-0.78669
RGS18	6.64E-05	0.00199	1.17E-08	0.478207	-0.46706	-0.94527
RGS19	8.94E-05	1.37E-07	1.34E-10	0.27138	-0.42358	-0.69496
RGS2	0.003289	0.014722	6.22E-07	0.282257	-0.26442	-0.54667
RGS4	0.004466	1.56E-05	9.01E-10	0.179976	-0.54812	-0.7281
RHBDF2	2.31E-06	8.10E-06	2.78E-11	0.331581	-0.35717	-0.68875
RHNO1	2.87E-08	4.45E-10	1.57E-11	0.386078	-0.61173	-0.99781
RHOBTB1	8.40E-06	9.69E-05	2.43E-10	0.519388	-0.54692	-1.06631
RHOG	2.65E-05	4.06E-06	4.19E-10	0.258134	-0.31236	-0.5705
RHOH	0.00047	1.60E-06	2.31E-10	0.219681	-0.44414	-0.66382
RHOQ	4.36E-08	6.70E-10	1.57E-11	0.264955	-0.38497	-0.64992
RIPK2	5.28E-06	2.89E-07	3.59E-11	0.199023	-0.30675	-0.50577
RMI2	0.000674	3.39E-07	3.36E-11	0.300402	-0.66471	-0.96511
RNASE2	0.126878	7.48E-07	9.01E-10	0.109996	-0.5005	-0.6105
RNASE6	2.49E-08	5.36E-08	1.57E-11	0.697291	-0.69466	-1.39195
RNASEH2A	1.21E-05	1.69E-05	3.97E-10	0.344616	-0.46682	-0.81143
RND2	0.001682	0.00199	1.23E-07	-0.27414	0.291099	0.565234
RND3	0.144149	9.89E-06	7.94E-08	0.153551	-0.46237	-0.61592
RNF103-CHMP3	4.48E-05	2.39E-09	2.78E-11	0.198818	-0.34844	-0.54726
RNF135	1.46E-05	5.39E-06	8.66E-11	0.314035	-0.33759	-0.65163
RNF166	6.90E-07	0.022279	2.54E-09	0.429244	-0.20654	-0.63578
ROBO1	2.16E-07	7.10E-06	1.77E-11	0.503542	-0.55211	-1.05565
RORC	0.126878	5.72E-11	5.92E-11	-0.08406	0.566128	0.650191
RP1-151F17.2	0.016372	7.59E-08	1.29E-08	-0.12634	0.475941	0.602281
RP1-193H18.2	0.232476	0.012702	0.000657	-0.14408	0.398756	0.542834
RP1-93H18.6	0.001925	0.003104	8.62E-09	0.475371	-0.61736	-1.09273
RP11-11N9.4	0.003419	7.59E-08	1.99E-10	0.285724	-0.82921	-1.11493
RP11-250B2.6	0.509837	2.00E-06	1.11E-06	-0.03528	0.561829	0.597113
RP11-327J17.2	0.073191	2.15E-05	1.48E-07	-0.15256	0.614998	0.767558
RP11-355B11.2	0.307401	2.15E-08	1.27E-08	-0.08418	0.617357	0.701541
RP11-384L8.1	0.159189	9.20E-10	5.62E-11	-0.12856	0.670508	0.799065
RP11-389C8.2	8.72E-05	0.025499	7.59E-08	0.335928	-0.18562	-0.52155
RP11-38P22.2	1.95E-05	1.38E-05	8.66E-11	0.316267	-0.45707	-0.77333
RP11-401P9.4	0.001603	0.001733	8.19E-09	0.277551	-0.33256	-0.61011
RP11-456H18.2	9.49E-05	0.000324	4.45E-09	-0.26046	0.242545	0.503

RP11-458D21.1	0.053563	0.001737	1.50E-05	-0.21018	0.42839	0.638568
RP11-486G15.2	0.224626	4.06E-06	2.05E-07	-0.09961	0.47912	0.578729
RP11-488L18.10	0.000481	0.081861	8.90E-07	0.429125	-0.21596	-0.64509
RP11-635N19.1	0.006643	1.10E-06	1.06E-10	-0.28459	0.538404	0.822997
RP11-642D21.1	0.001391	0.43409	0.000101	0.344541	-0.19225	-0.5368
RP11-686D22.8	9.49E-05	1.61E-08	5.92E-11	0.292382	-0.49398	-0.78636
RP11-740C1.2	0.26255	1.21E-05	1.06E-08	-0.18102	0.675831	0.856849
RP11-747H7.3	8.67E-06	7.74E-05	3.07E-10	0.348108	-0.37047	-0.71858
RP11-863K10.7	0.000547	2.87E-06	2.08E-10	-0.18882	0.329118	0.517936
RP11-96D1.11	0.067962	8.26E-08	2.95E-09	-0.08471	0.447144	0.531857
RP3-406A7.7	0.031057	7.10E-06	1.78E-07	-0.12138	0.384456	0.505836
RP4-680D5.8	0.173653	1.12E-09	1.41E-07	-0.03895	0.65983	0.698779
RP4-791M13.3	0.053563	0.001737	1.50E-05	-0.21018	0.42839	0.638568
RP5-1154L15.2	0.811944	0.001205	0.000436	0.033695	-0.50505	-0.53874
RPL22L1	0.028997	1.26E-09	8.29E-11	0.174344	-0.81843	-0.99278
RPL39L	0.029978	2.84E-08	1.48E-10	0.174135	-0.5226	-0.69674
RPS6KA1	0.000281	0.000109	2.18E-09	0.318568	-0.34301	-0.66158
RRAS	0.016925	0.000211	4.67E-09	0.193652	-0.33298	-0.52663
RRM2	3.04E-06	4.67E-07	2.04E-11	1.088212	-1.49086	-2.57907
RRM2B	1.40E-06	0.05377	6.68E-09	0.395079	-0.17824	-0.57332
RSAD2	0.000188	0.496563	2.49E-05	0.537375	-0.28332	-0.8207
RTN1	8.61E-09	9.17E-05	2.49E-11	0.616365	-0.54225	-1.15861
RTP4	9.97E-07	5.39E-06	1.79E-10	0.454333	-0.54875	-1.00309
RUNX1-IT1	0.000274	0.012232	2.47E-07	0.289814	-0.23459	-0.52441
RUNX3	1.73E-07	1.56E-05	4.29E-11	0.326596	-0.31621	-0.64281
RXRA	0.021277	1.61E-08	3.97E-10	-0.11833	0.394266	0.512593
S100A11	1.63E-09	2.98E-12	1.57E-11	0.622241	-0.79618	-1.41842
S100A11P1	1.30E-06	8.31E-10	2.21E-11	0.346747	-0.59373	-0.94048
S100A14	0.10091	0.173471	0.001462	0.191666	-0.34394	-0.5356
S100A4	6.54E-09	5.36E-08	2.38E-11	0.709702	-0.64107	-1.35078
S100A6	6.71E-07	7.48E-07	2.87E-11	0.342556	-0.44381	-0.78637
S100A8	0.50179	6.09E-05	2.68E-05	0.10774	-0.42559	-0.53333
S100A9	0.169737	4.49E-05	3.15E-06	0.109087	-0.5481	-0.65719
SACS	4.44E-06	2.89E-07	2.43E-11	0.393643	-0.57127	-0.96491
SAMD11	0.010441	2.03E-05	3.48E-11	0.284482	-0.56809	-0.85257
SAMD9	2.62E-07	7.57E-06	9.63E-11	0.486686	-0.52339	-1.01008
SAMD9L	1.34E-05	2.93E-05	2.43E-10	0.411702	-0.56333	-0.97503
SAMHD1	4.27E-08	0.000171	2.43E-11	0.440054	-0.37729	-0.81734
SAMSN1	3.45E-08	1.38E-06	2.58E-11	0.40567	-0.48265	-0.88832
SASH3	2.02E-08	4.16E-08	1.77E-11	0.541373	-0.65765	-1.19903
SAT2	0.018194	1.95E-09	1.95E-11	-0.14043	0.405376	0.545803
SC5D	0.175754	1.60E-06	9.21E-08	-0.09253	0.459854	0.552388

SCARA5	0.065076	7.10E-06	0.000436	0.16153	0.510619	0.349089
SCD	0.698268	0.000115	3.63E-05	0.05624	-0.56184	-0.61808
SCG5	0.04899	5.39E-05	2.61E-08	-0.26115	0.68451	0.945656
SCGN	0.371309	0.006623	0.000202	0.117151	-0.55471	-0.67186
SCRN1	2.43E-07	6.97E-07	2.13E-11	0.417517	-0.61437	-1.03188
SDC2	0.024383	1.73E-10	1.01E-10	-0.11386	0.439372	0.553235
SDCBP2	0.522219	0.001205	2.79E-05	0.114173	-0.80724	-0.92141
SECTM1	2.48E-06	0.000667	1.27E-10	0.355577	-0.31282	-0.6684
SEL1L3	5.12E-10	8.76E-12	1.57E-11	0.37233	-0.51687	-0.8892
SELENBP1	0.022024	2.03E-05	4.23E-09	-0.14554	0.366071	0.511614
SELL	1.90E-09	0.000171	5.42E-11	0.707811	-0.61	-1.31781
SELM	7.18E-07	9.20E-10	1.57E-11	0.561655	-0.8761	-1.43775
SEMA3C	0.001758	1.78E-05	1.15E-10	0.249697	-0.45465	-0.70434
SEMA4D	8.61E-09	4.67E-07	1.64E-11	0.479707	-0.39946	-0.87917
6-Sep	3.69E-06	1.90E-05	7.70E-10	0.345566	-0.25045	-0.59601
SERPINA5	0.002107	3.93E-10	7.95E-11	-0.11908	0.457033	0.57611
SERPINB1	1.19E-07	1.08E-08	2.87E-11	0.300519	-0.37095	-0.67147
SERPINB8	0.019115	3.83E-12	1.70E-11	0.140102	-0.55092	-0.69102
SERPINB9	1.80E-06	6.37E-08	1.15E-10	0.313065	-0.3645	-0.67757
SERPINE2	1.07E-06	5.04E-06	1.06E-10	0.273499	-0.43541	-0.70891
SERPINF2	0.010626	1.16E-10	2.87E-11	-0.12395	0.465154	0.589107
SERPINH1	0.023164	4.37E-06	1.58E-08	0.17593	-0.43265	-0.60858
SFRP5	0.049705	0.007761	4.53E-05	0.195177	-0.38248	-0.57766
SFXN3	3.45E-08	4.06E-06	1.85E-11	0.285179	-0.24748	-0.53265
SGCB	0.013515	0.000324	8.79E-08	0.186583	-0.35296	-0.53954
SGK1	0.001603	0.000667	7.00E-10	0.335934	-0.38222	-0.71815
SGK223	0.000208	5.86E-08	3.73E-11	0.291491	-0.58515	-0.87664
SGOL2	0.000193	1.86E-06	3.40E-10	0.25491	-0.53919	-0.7941
SH3BGRL	8.95E-09	4.35E-12	1.57E-11	0.355378	-0.52768	-0.88306
SH3BGRL3	0.000198	2.09E-07	7.00E-10	0.283053	-0.48164	-0.76469
SHCBP1	0.063194	6.74E-09	3.40E-10	0.093593	-0.62978	-0.72337
SHD	0.006779	4.22E-05	3.11E-09	-0.19809	0.355022	0.553109
SHF	0.460806	5.51E-07	1.55E-08	-0.08854	0.567896	0.65644
SIDT1	9.91E-06	9.27E-06	3.58E-10	0.345051	-0.46117	-0.80622
SIGIRR	0.012768	8.67E-05	1.58E-08	-0.243	0.472771	0.715774
SIGLEC1	3.48E-06	0.006897	1.58E-08	0.388654	-0.31273	-0.70138
SIGLEC10	0.011462	3.07E-06	2.66E-10	0.177682	-0.51031	-0.68799
SIRPG	0.000674	5.79E-06	2.92E-10	0.197188	-0.37621	-0.5734
SKA2	4.92E-06	4.22E-05	7.37E-10	0.330985	-0.34219	-0.67317
SKAP1	0.007606	0.001205	1.41E-07	-0.23999	0.359921	0.599907
SKIDA1	0.464651	5.44E-09	4.67E-09	-0.04325	0.518806	0.562054
SLA	1.15E-08	6.37E-08	1.57E-11	0.481307	-0.56931	-1.05062
SLAMF6	0.118714	7.10E-06	2.61E-08	0.106346	-0.41963	-0.52598
SLAMF7	5.98E-08	5.04E-06	2.58E-11	0.511934	-0.72864	-1.24057
SLAMF8	3.61E-09	1.95E-09	1.57E-11	0.623837	-1.00506	-1.6289
SLC12A2	8.43E-08	4.91E-08	1.69E-11	0.641089	-0.98586	-1.62695
SLC13A5	0.055177	7.59E-08	3.58E-10	-0.1678	0.715872	0.883675
SLC15A3	1.83E-05	3.74E-05	4.67E-10	0.365687	-0.43961	-0.80529
SLC16A10	0.003492	9.93E-09	1.10E-10	-0.28158	1.061708	1.343289
SLC16A7	7.73E-05	0.378944	2.66E-06	0.448211	-0.12021	-0.56843
SLC17A2	0.001682	2.48E-10	1.95E-11	-0.18627	0.628094	0.814367
SLC18B1	7.54E-06	4.70E-06	3.36E-11	0.388033	-0.41022	-0.79825
SLC19A2	0.051263	1.16E-07	9.52E-09	-0.14634	0.637858	0.784194
SLC22A1	0.005041	7.37E-10	5.62E-11	-0.15214	0.598859	0.750996
SLC22A15	0.051263	4.22E-05	2.98E-07	0.154187	-0.85364	-1.00782
SLC22A25	0.095582	0.000211	5.94E-07	-0.1187	0.383917	0.50262
SLC25A33	0.131933	0.000211	3.15E-06	0.152916	-0.49597	-0.64889

SLC26A2	3.39E-05	1.01E-09	1.72E-11	0.240949	-0.43783	-0.67878
SLC27A5	0.000367	3.24E-09	4.72E-11	-0.17287	0.401865	0.574737
SLC28A3	0.021653	0.000947	3.95E-07	0.150414	-0.38154	-0.53195
SLC29A3	0.00069	3.29E-06	1.36E-09	0.234098	-0.35522	-0.58932
SLC2A3	6.44E-05	3.14E-07	3.91E-11	0.201217	-0.30446	-0.50568
SLC2A6	0.000367	1.13E-05	1.15E-10	0.242682	-0.37003	-0.61271
SLC35G1	0.001326	2.43E-05	4.23E-09	0.217732	-0.42412	-0.64185
SLC37A4	0.002778	1.95E-09	4.67E-10	-0.1761	0.371951	0.548051
SLC38A1	5.37E-05	2.81E-10	3.59E-11	0.262961	-0.56264	-0.8256
SLC38A4	0.062274	4.70E-06	1.94E-08	-0.14523	0.51371	0.658944
SLC38A6	9.25E-06	6.99E-06	5.17E-11	0.41832	-0.55296	-0.97128
SLC39A10	1.60E-07	1.28E-06	1.82E-11	0.514737	-0.43957	-0.95431
SLC39A5	0.007606	4.91E-08	3.23E-10	-0.16701	0.391458	0.558469
SLC44A2	9.49E-05	0.000121	6.68E-10	0.265951	-0.25077	-0.51672
SLC44A3	0.001494	2.58E-05	6.01E-10	0.593262	-0.86008	-1.45335
SLC45A3	0.392802	2.91E-09	6.34E-10	-0.06357	0.487977	0.551547
SLC45A4	1.38E-07	3.24E-09	1.60E-11	0.253219	-0.39345	-0.64667
SLC47A1	0.000392	1.01E-09	2.66E-10	-0.20889	0.596584	0.805476
SLC51A	0.009337	2.15E-05	2.42E-09	0.347838	-0.50225	-0.85009
SLC51B	0.064163	1.06E-10	3.59E-11	0.24771	-1.73836	-1.98607
SLC6A1	0.126878	6.10E-10	1.78E-09	-0.04995	0.671058	0.721011
SLC6A13	0.016136	3.24E-09	1.06E-10	-0.11029	0.451382	0.561669
SLC6A16	0.350312	1.97E-08	5.44E-08	-0.06625	0.688769	0.755018
SLC7A6	1.38E-05	7.36E-09	1.69E-11	0.183063	-0.39183	-0.57489
SLC7A7	1.03E-08	1.38E-05	2.49E-11	0.518882	-0.50126	-1.02014
SLC9B2	0.000338	8.07E-07	2.92E-10	-0.20662	0.395476	0.602099
SLCO1B1	0.133573	1.51E-10	6.34E-09	-0.05687	0.472866	0.529737
SLFN11	2.87E-08	2.84E-08	1.77E-11	0.506517	-0.54545	-1.05197
SLFN12	1.05E-08	6.82E-12	1.57E-11	0.42673	-1.00625	-1.43298
SLFN13	0.000117	2.31E-06	6.94E-11	0.239761	-0.49308	-0.73284
SLFN5	4.75E-05	0.000858	1.41E-10	0.264459	-0.31261	-0.57707
SLIT2	0.000203	9.17E-05	1.00E-09	0.243353	-0.40962	-0.65298
SLITRK3	0.014018	0.008722	4.33E-07	-0.29469	0.350886	0.645575
SMAD9	0.038373	0.000144	4.67E-09	0.134291	-0.38478	-0.51908
SMARCA	1.40E-06	6.37E-08	2.78E-11	0.303709	-0.39005	-0.69376
SMC2	1.87E-06	4.49E-05	1.06E-10	0.359565	-0.33777	-0.69733
SMC4	0.001215	3.53E-05	7.01E-09	0.195771	-0.32373	-0.5195
SMIM1	0.050444	1.90E-05	1.85E-08	-0.17321	0.357904	0.531118
SMIM14	0.403595	3.66E-07	1.23E-07	-0.03883	0.540748	0.579583
SMIM2-AS1	0.065076	0.012232	1.71E-06	-0.22195	0.338475	0.560426
SMIM3	1.42E-05	0.000249	2.08E-10	0.423016	-0.37664	-0.79966
SMOC1	0.157227	5.44E-09	1.68E-09	-0.07415	0.523436	0.597582
SMOC2	0.014018	0.0002	2.95E-09	0.276807	-0.5837	-0.86051
SMPX	0.333286	0.050673	0.000188	0.257028	-0.39382	-0.65085
SMS	0.005147	5.36E-08	3.58E-10	0.176474	-0.33481	-0.51128
SNAP25	0.040115	0.000115	1.02E-07	0.148053	-0.42273	-0.57078
SNHG12	0.000198	2.15E-08	2.96E-11	0.220354	-0.4261	-0.64645
SNN	1.10E-05	3.53E-06	9.01E-10	0.23362	-0.3207	-0.55432
SNORA16A	0.000198	2.15E-08	2.96E-11	0.220354	-0.4261	-0.64645
SNORA44	0.000198	2.15E-08	2.96E-11	0.220354	-0.4261	-0.64645
SNORA61	0.000198	2.15E-08	2.96E-11	0.220354	-0.4261	-0.64645
SNX7	0.022764	3.79E-06	1.29E-09	0.179372	-0.35734	-0.53672
SORD	0.059617	1.95E-09	2.92E-10	-0.0926	0.612	0.704597
SORL1	0.034291	2.48E-10	9.01E-10	-0.17285	0.586246	0.759095
SORT1	0.112549	2.58E-05	1.21E-06	0.079282	-0.47365	-0.55293
SOWAHC	0.073191	5.04E-06	2.95E-09	-0.12144	0.403395	0.52484

SOX4	6.78E-06	5.86E-08	1.91E-11	0.29363	-0.49335	-0.78698
SOX9	3.58E-06	4.16E-08	2.58E-11	0.901472	-1.38103	-2.2825
SP110	1.74E-06	0.000635	5.16E-10	0.306962	-0.28204	-0.589
SP140	3.67E-07	5.73E-05	1.29E-09	0.369761	-0.45881	-0.82857
SPA17	0.002605	9.09E-09	7.95E-11	0.244011	-0.69145	-0.93546
SPAG1	0.03264	0.000103	2.14E-08	0.308446	-0.43225	-0.7407
SPARC	9.49E-05	1.47E-05	1.51E-09	0.306185	-0.44138	-0.74757
SPATS2	3.97E-07	6.97E-07	2.78E-11	0.244634	-0.36796	-0.61259
SPC25	0.002723	5.39E-05	9.01E-10	0.260295	-0.62433	-0.88462
SPDL1	6.06E-05	0.000211	5.72E-10	0.269491	-0.34378	-0.61327
SPIN4	1.03E-05	0.000263	9.01E-10	0.444548	-0.37226	-0.81681
SPINK1	0.042642	0.125799	0.000164	0.292363	-0.49823	-0.7906
SPINT2	5.28E-06	5.97E-07	4.29E-11	0.482614	-0.89069	-1.37331
SPON1	0.006384	8.17E-05	1.43E-09	0.187465	-0.41247	-0.59993
SPON2	1.73E-05	7.59E-08	3.48E-11	0.409567	-0.64897	-1.05854
SPP1	8.94E-05	2.68E-06	7.28E-11	0.637113	-0.87755	-1.51466
SPSB1	0.020512	3.33E-05	3.52E-08	0.212385	-0.41025	-0.62263
SPTSSA	0.028512	0.055229	5.94E-06	0.274138	-0.24376	-0.51789
SRD5A1	0.145789	5.39E-06	7.11E-07	-0.08793	0.489247	0.577181
SRD5A2	0.017877	2.91E-09	1.06E-10	-0.36686	1.424293	1.791158
SRGN	1.55E-06	3.66E-07	2.13E-11	0.352218	-0.47152	-0.82373
SRPX	0.000726	0.000293	3.45E-09	0.435911	-0.58391	-1.01982
SRXN1	0.004939	3.66E-07	1.06E-10	0.173805	-0.42991	-0.60371
SS18L1	0.046156	6.37E-08	4.23E-09	-0.10851	0.465356	0.573866
SSPN	0.000726	0.0009	5.16E-10	0.276395	-0.32061	-0.59701
ST3GAL1	0.03264	4.06E-06	5.97E-08	-0.15893	0.389215	0.548141
ST3GAL6	0.019802	2.09E-07	1.59E-09	-0.13561	0.413333	0.548941
ST8SIA4	3.60E-08	8.71E-06	2.31E-11	0.399775	-0.43561	-0.83538
STAMBPL1	5.46E-06	6.28E-11	1.69E-11	0.202104	-0.38933	-0.59144
STARD3NL	2.94E-07	2.59E-08	2.58E-11	0.305446	-0.40566	-0.71111
STAT1	8.95E-08	2.46E-07	1.57E-11	0.653964	-0.89358	-1.54754
STEAP4	0.001925	0.001453	3.35E-08	0.220539	-0.28588	-0.50642
STIL	0.00225	5.79E-06	3.58E-10	0.205553	-0.56013	-0.76568
STK10	1.10E-09	3.39E-07	1.57E-11	0.379056	-0.38079	-0.75985
STK17A	1.51E-08	6.70E-10	1.57E-11	0.347932	-0.45343	-0.80136
STK17B	1.01E-08	2.59E-08	1.57E-11	0.349249	-0.48229	-0.83153
STK39	7.27E-06	1.40E-09	1.70E-11	0.392289	-0.76146	-1.15375
STMN2	0.055177	2.91E-09	8.66E-11	0.193239	-1.17744	-1.37068
STX11	1.13E-05	0.001151	2.07E-09	0.290478	-0.3219	-0.61238
SUCO	0.001391	1.06E-05	4.67E-09	0.30127	-0.35547	-0.65674
SULF2	0.017251	0.09585	4.53E-05	0.290149	-0.2236	-0.51375
SULT1E1	0.489198	5.39E-05	1.32E-06	-0.09682	0.707383	0.804206
SUSD1	1.42E-05	9.27E-06	9.12E-11	0.386206	-0.48867	-0.87488
SWAP70	2.49E-08	5.44E-09	1.57E-11	0.306269	-0.30968	-0.61595
SYK	2.82E-08	3.07E-06	1.95E-11	0.294781	-0.31456	-0.60934
SYPL2	0.866472	3.07E-06	1.62E-07	-0.03141	0.623094	0.654503
SYS1-DBNDD2	1.62E-08	4.06E-06	5.17E-11	0.386218	-0.38794	-0.77416
SYT13	0.149521	6.66E-06	9.21E-08	0.094469	-0.44333	-0.5378
SYT17	0.049705	0.013186	5.06E-05	-0.22358	0.396023	0.619599
SYTL1	1.28E-07	0.000401	3.59E-11	0.413351	-0.2901	-0.70346
TACC3	0.000169	1.10E-06	2.19E-10	0.308666	-0.53892	-0.84759
TAGAP	2.82E-08	3.29E-06	2.08E-10	0.236682	-0.28252	-0.51921
TAGLN	0.167606	1.38E-05	4.91E-09	0.16864	-0.64371	-0.81235
TAGLN2	0.000547	4.91E-10	1.34E-10	0.225309	-0.53314	-0.75845
TAP1	8.67E-08	2.46E-07	1.58E-11	0.585045	-0.76062	-1.34566

TAP2	2.65E-05	0.000362	2.43E-10	0.294109	-0.30663	-0.60074
TARP	5.15E-08	1.66E-05	4.51E-11	0.489113	-0.40545	-0.89456
TAT	0.784859	0.001205	7.06E-05	-0.04439	0.463518	0.507912
TAX1BP3	2.21E-05	6.28E-11	1.69E-11	0.384727	-0.73882	-1.12355
TBC1D10C	1.07E-06	1.37E-07	5.42E-11	0.398698	-0.4995	-0.8982
TBC1D2	0.121197	6.20E-06	7.94E-08	-0.12054	0.414921	0.53546
TC2N	0.000612	3.63E-09	3.91E-11	0.136289	-0.36428	-0.50056
TCEA3	0.09688	1.58E-09	2.31E-10	-0.08871	0.439777	0.528491
TCEAL3	3.69E-06	5.00E-09	1.85E-11	0.335253	-0.46097	-0.79622
TCEAL7	0.063786	0.014191	5.46E-05	0.190844	-0.33139	-0.52223
TCEAL8	0.002199	5.36E-08	3.40E-10	0.19171	-0.42499	-0.6167
TCEB3-AS1	0.014761	2.89E-07	3.07E-10	-0.18604	0.458027	0.644068
TCF19	7.74E-07	9.09E-09	1.69E-11	0.392021	-0.76459	-1.15661
TCF4	2.94E-08	3.29E-06	2.04E-11	0.392803	-0.35524	-0.74804
TCHH	0.16675	0.016948	1.02E-06	0.184871	-0.35711	-0.54198
TCIRG1	2.09E-07	2.15E-05	7.37E-10	0.309509	-0.31401	-0.62352
TCL1A	0.000742	0.005625	4.12E-07	0.245439	-0.40582	-0.65126
TCTEX1D2	0.008665	0.002595	1.28E-07	0.197786	-0.32615	-0.52393
TDGF1	0.464651	0.069473	0.000511	0.135209	-0.36667	-0.50188
TDGF1P3	0.464651	0.069473	0.000511	0.135209	-0.36667	-0.50188
TDRD6	0.240437	0.001517	6.33E-05	-0.12974	0.404906	0.534646
TDRD7	8.94E-05	0.002175	6.68E-09	0.274862	-0.28034	-0.5552
TENM1	0.036005	2.29E-05	6.68E-09	-0.14547	0.365202	0.510673
TENM2	0.707231	0.029892	0.005111	-0.06198	0.615908	0.677892
TES	0.01561	2.81E-10	1.21E-10	0.133163	-0.41837	-0.55153
TESC	0.021653	1.38E-06	5.43E-09	0.144424	-0.42177	-0.5662
TFEC	8.95E-09	2.75E-05	1.72E-11	0.523319	-0.53762	-1.06094
TFPI2	0.01428	0.264635	7.60E-05	0.354174	-0.26772	-0.62189
TFR2	0.004292	1.97E-08	3.23E-10	-0.1429	0.402766	0.545663
TGFB1I1	0.013515	0.005389	1.38E-06	0.317212	-0.4044	-0.72161
TGFBI	0.007129	3.45E-08	8.66E-11	0.200183	-0.50915	-0.70934
THBD	0.004376	0.011798	1.11E-06	0.284052	-0.24617	-0.53022
THBS1	0.000138	2.59E-08	5.72E-10	0.225865	-0.51513	-0.741
THBS2	2.08E-08	1.73E-10	1.57E-11	0.826364	-1.32263	-2.149
THEMIS	3.55E-07	7.48E-07	1.95E-11	0.483682	-0.6747	-1.15838
THEMIS2	3.36E-06	0.000605	5.45E-10	0.448753	-0.42466	-0.87342
THOP1	0.068961	2.29E-05	2.25E-07	-0.1544	0.658092	0.812492
THRB-IT1	0.285682	8.26E-08	2.14E-08	-0.06726	0.639187	0.706449
THRSP	0.020174	0.148123	0.00024	-0.33841	0.545893	0.884308
THSD7A	0.020174	0.018189	3.24E-05	0.155853	-0.41612	-0.57197
THY1	0.000585	6.37E-08	1.01E-10	0.212819	-0.74017	-0.95299
TIGIT	4.06E-05	8.71E-06	5.16E-10	0.295272	-0.50852	-0.80379
TIMD4	0.142343	0.075369	0.002255	0.172397	-0.36264	-0.53504
TIMP1	5.37E-05	1.78E-11	1.77E-11	0.281215	-0.90299	-1.18421
TIMP2	9.79E-08	4.67E-07	3.48E-11	0.418803	-0.44859	-0.86739
TK1	4.61E-05	0.001517	1.67E-08	0.293909	-0.38999	-0.6839
TLCD1	0.177862	1.06E-05	2.25E-07	0.12522	-0.45184	-0.57706
TLR1	0.000288	0.00052	2.54E-09	0.527291	-0.48782	-1.01511
TLR2	1.24E-07	0.000109	3.07E-11	0.502832	-0.47397	-0.9768
TLR4	4.29E-06	0.011356	1.11E-08	0.334659	-0.2193	-0.55396
TLR5	0.01879	1.61E-08	4.91E-10	0.178917	-0.44375	-0.62267
TLR7	3.04E-06	0.000211	4.29E-11	0.239496	-0.27267	-0.51216
TLR8	8.97E-10	9.17E-05	1.57E-11	0.653493	-0.53805	-1.19154
TM6SF1	9.79E-08	6.97E-07	1.58E-11	0.620852	-0.66786	-1.28872
TM6SF2	0.001239	0.000308	5.43E-09	-0.21259	0.298647	0.511239

TMC4	0.016372	0.00159	1.64E-06	0.275502	-0.74023	-1.01573
TMC6	1.07E-06	1.47E-05	1.15E-10	0.268373	-0.28091	-0.54929
TMEM100	0.011247	0.075369	9.69E-06	0.443063	-0.38727	-0.83033
TMEM120A	0.006506	5.73E-05	2.54E-09	-0.19614	0.326311	0.522453
TMEM125	0.24863	0.000947	8.93E-06	0.151428	-0.5655	-0.71693
TMEM154	0.001882	0.021535	1.02E-07	0.82711	-0.55812	-1.38523
TMEM164	2.08E-06	4.52E-09	1.69E-11	0.246024	-0.39898	-0.645
TMEM173	9.91E-06	4.31E-07	4.51E-11	0.24887	-0.35567	-0.60454
TMEM200A	7.94E-05	9.47E-07	2.08E-10	0.333895	-0.86179	-1.19568
TMEM220	0.020174	0.00052	5.20E-08	-0.20998	0.358806	0.568788
TMEM243	1.62E-08	7.37E-10	1.60E-11	0.562129	-0.58587	-1.14799
TMEM255A	0.017877	0.001205	1.76E-08	0.404544	-0.78171	-1.18625
TMEM256	0.000383	1.86E-06	2.79E-10	-0.24377	0.363332	0.607106
TMEM44-AS1	0.043338	4.49E-05	1.44E-06	-0.16253	0.436069	0.598598
TMEM45B	0.273879	0.023046	0.00102	0.188922	-0.78644	-0.97536
TMEM50A	9.47E-08	3.53E-10	1.57E-11	0.265955	-0.35759	-0.62354
TMEM51	2.07E-05	4.91E-10	2.49E-11	0.266145	-0.53955	-0.80569
TMEM52	0.460806	1.21E-05	1.27E-06	-0.08051	0.549528	0.630036
TMEM55A	0.000208	2.15E-05	3.97E-10	0.303551	-0.49995	-0.8035
TMEM56	0.175754	2.11E-11	1.02E-07	-0.04557	0.4947	0.540267
TMEM87B	7.85E-08	0.000547	2.31E-10	0.34486	-0.2706	-0.61546
TMPRSS3	0.015053	1.21E-05	2.42E-09	0.155177	-0.60481	-0.75999
TMSB10	3.61E-09	1.06E-12	1.57E-11	0.463564	-0.73015	-1.19371
TMSB4X	6.78E-06	2.34E-11	1.57E-11	0.263705	-0.44223	-0.70594
TNF	0.031573	2.75E-05	3.11E-07	0.12816	-0.42051	-0.54867
TNFAIP3	2.74E-06	1.02E-06	2.43E-11	0.404329	-0.49054	-0.89487
TNFAIP8	2.44E-10	8.26E-08	1.57E-11	0.566574	-0.57454	-1.14112
TNFAIP8L2	2.80E-05	3.12E-05	2.81E-09	0.281931	-0.36473	-0.64666
TNFRSF11A	0.000412	0.001043	8.19E-09	0.29186	-0.32507	-0.61693
TNFRSF11B	0.000762	0.000447	7.40E-09	0.304943	-0.33481	-0.63975
TNFRSF12A	0.044064	2.09E-07	8.19E-09	0.182359	-0.62197	-0.80433
TNFRSF17	3.95E-08	2.64E-05	1.85E-11	0.756359	-1.0001	-1.75646
TNFRSF1B	1.10E-05	1.56E-05	1.88E-10	0.337431	-0.42766	-0.76509
TNFRSF21	8.15E-09	3.45E-08	1.57E-11	0.504902	-0.7281	-1.233
TNFSF13B	4.03E-09	4.67E-07	1.57E-11	0.661137	-0.74238	-1.40352
TOMM40L	0.011025	1.97E-08	3.23E-10	0.20365	-0.59196	-0.79561
TOP2A	6.44E-05	8.07E-07	2.49E-11	0.410905	-0.87369	-1.2846
TOX	1.13E-05	3.07E-06	6.26E-11	0.361478	-0.47673	-0.83821
TOX2	0.019802	0.023046	2.22E-05	0.275785	-0.2522	-0.52799
TOX3	3.39E-05	0.046278	1.17E-08	0.423182	-0.18612	-0.6093
TP53BP2	0.003492	6.20E-06	2.56E-10	0.180632	-0.35212	-0.53275
TP53I3	0.007751	8.26E-08	1.48E-10	0.398565	-0.81293	-1.21149
TPM1	0.001294	7.48E-07	4.42E-10	0.233269	-0.58276	-0.81602
TPPP2	0.000944	6.47E-07	7.28E-11	-0.27749	0.550334	0.827827
TPX2	5.46E-06	0.000115	1.11E-09	0.482373	-0.63221	-1.11458
TRAC	8.95E-09	5.86E-08	1.57E-11	0.359155	-0.4253	-0.78446
TRAF3IP3	7.49E-09	0.000115	3.73E-11	0.410479	-0.33983	-0.75031

TRAF5	3.40E-09	5.47E-10	1.57E-11	0.37507	-0.36208	-0.73715
TRAJ17	1.79E-08	1.38E-06	1.68E-11	0.442227	-0.47013	-0.91236
TRAPPC2 P1	0.000392	2.93E-05	4.45E-09	0.213946	-0.32407	-0.53801
TRAT1	8.94E-05	8.10E-06	1.79E-10	0.370883	-0.57664	-0.94752
TRBC1	3.58E-09	6.06E-09	1.57E-11	0.913184	-0.98191	-1.89509
TRDV2	1.79E-08	1.38E-06	1.68E-11	0.442227	-0.47013	-0.91236
TREM2	0.10091	3.66E-07	1.06E-10	0.175334	-0.66355	-0.83889
TRG-AS1	0.00567	0.000308	4.71E-08	0.307736	-0.45301	-0.76075
TRGC2	2.94E-08	1.56E-05	2.87E-11	0.762829	-0.59312	-1.35595
TRGV9	2.94E-08	1.56E-05	2.87E-11	0.762829	-0.59312	-1.35595
TRIM21	3.49E-05	2.15E-08	2.38E-11	0.254479	-0.47202	-0.7265
TRIM22	1.99E-08	2.87E-06	2.21E-11	0.850256	-0.58536	-1.43561
TRIM31	0.056022	6.97E-07	1.06E-09	0.104679	-0.50467	-0.60935
TRIM55	0.425639	0.073376	0.134724	0.109251	0.536326	0.427075
TRIM6	0.009859	0.000992	1.67E-08	0.315091	-0.44162	-0.75671
TRIM69	0.000156	6.09E-05	2.92E-10	0.237347	-0.29877	-0.53612
TRIP13	0.036005	7.48E-07	3.97E-10	0.1593	-0.58661	-0.74591
TRNP1	0.229723	9.33E-11	3.36E-11	0.064956	-0.52645	-0.59141
TSLP	0.04899	1.48E-08	1.14E-05	0.208206	0.686311	0.478105
TSPAN8	0.780458	0.026336	0.000989	0.165179	-0.73713	-0.90231
TSPO	0.114086	0.000144	3.68E-08	0.148278	-0.39031	-0.53859
TSPYL5	0.004466	0.000121	1.11E-07	0.374297	-0.55823	-0.93253
TTC36	7.05E-05	4.91E-10	1.57E-11	-0.35683	0.886764	1.243595
TTK	0.000151	9.89E-06	6.01E-10	0.423981	-0.9435	-1.36748
TTPA	0.357219	9.17E-05	0.0007	0.112393	0.544705	0.432312
TUBA1A	1.31E-08	5.72E-11	1.57E-11	0.648424	-0.77991	-1.42834
TUBA1B	2.80E-05	4.35E-12	1.64E-11	0.214709	-0.43311	-0.64782
TUBA1C	0.000193	1.05E-12	1.57E-11	0.228245	-0.51813	-0.74638
TUBB6	1.29E-05	2.59E-08	1.57E-11	0.398119	-0.59943	-0.99755
TUBG1	0.000245	5.79E-06	1.17E-09	0.253445	-0.35267	-0.60612
TUSC3	2.55E-06	0.001737	1.29E-08	0.271378	-0.2755	-0.54688
TWSG1	0.000203	0.001517	7.01E-09	0.246582	-0.27254	-0.51912
TYMP	0.000329	0.000103	2.08E-10	0.357509	-0.51441	-0.87192
TYMS	5.15E-08	3.78E-08	1.57E-11	0.649445	-0.65507	-1.30451
TYMSOS	3.29E-05	0.004364	2.66E-10	0.397275	-0.33654	-0.73382
TYROBP	1.34E-05	1.93E-07	8.29E-11	0.430452	-0.64558	-1.07604
UAP1L1	5.60E-07	7.74E-05	1.51E-09	0.293866	-0.32922	-0.62309
UBASH3A	0.000281	1.47E-05	9.63E-11	0.446978	-0.57127	-1.01825
UBD	4.87E-09	8.26E-08	1.57E-11	1.817402	-1.58444	-3.40184
UBE2A	2.39E-06	1.62E-11	1.57E-11	0.194673	-0.31985	-0.51452
UBE2C	0.000169	2.67E-07	9.12E-11	0.307872	-0.73273	-1.04061
UBE2L6	9.49E-05	2.29E-05	3.07E-10	0.338039	-0.50501	-0.84305
UBE2Q2	9.27E-07	5.86E-08	1.77E-11	0.545429	-0.5613	-1.10673
UBE2S	0.000274	9.27E-06	4.67E-10	0.344237	-0.55557	-0.89981
UBE2T	1.29E-05	1.30E-10	1.68E-11	0.437438	-0.91216	-1.3496
UBLCP1	1.42E-05	2.29E-05	3.07E-10	0.369643	-0.26499	-0.63464
UCP2	3.25E-06	1.38E-05	2.56E-10	0.4374	-0.5684	-1.0058
UGCG	0.00245	0.000401	9.52E-09	0.312212	-0.37504	-0.68725
UGT2A3	0.007606	0.231418	3.76E-05	0.48546	-0.29488	-0.78034
UHRF1	0.008665	9.93E-09	1.64E-10	0.393208	-1.43225	-1.82546
UPB1	0.000281	5.86E-08	6.59E-11	-0.20673	0.470006	0.676733
UPP2	0.001531	0.027147	1.79E-06	0.733981	-0.68412	-1.4181
UTS2	0.020512	3.53E-05	2.95E-09	0.276276	-0.77209	-1.04836
UXS1	1.40E-06	1.26E-09	1.91E-11	0.299878	-0.41055	-0.71043
VAMP5	0.002065	4.53E-08	3.23E-10	0.181448	-0.45671	-0.63816
VAV1	0.000238	0.002715	3.52E-08	0.249122	-0.30575	-0.55487
VCAN	1.11E-06	8.73E-07	1.69E-11	0.453988	-0.70119	-1.15518

VEGFC	0.000506	0.003375	6.26E-08	0.358296	-0.35261	-0.7109
VIL1	0.032081	0.181223	0.000202	-0.30287	0.239122	0.541992
VIM	2.02E-08	1.01E-09	1.57E-11	0.359706	-0.47983	-0.83953
VMO1	0.024816	0.0007	5.94E-07	0.154572	-0.35558	-0.51015
VMP1	0.002344	9.33E-11	1.70E-11	0.164232	-0.51073	-0.67497
VNN2	0.000742	0.000383	2.36E-08	0.235394	-0.35736	-0.59276
VOPP1	3.61E-09	1.05E-12	1.57E-11	0.373048	-0.59423	-0.96728
VRK1	3.17E-05	1.95E-09	1.69E-11	0.352812	-0.58393	-0.93674
VSIG10L	0.002668	0.000547	1.06E-07	0.271128	-0.35361	-0.62474
VSIG4	0.002837	1.78E-05	6.68E-10	0.251531	-0.45208	-0.70361
VSNL1	0.050444	0.000128	2.59E-07	-0.10966	0.471496	0.581158
VTCN1	0.091674	2.84E-08	9.63E-11	0.169711	-0.92826	-1.09798
VWA5A	1.06E-05	5.39E-06	3.07E-11	0.302049	-0.3636	-0.66565
WARS	0.000117	2.09E-07	2.87E-11	0.339076	-0.64161	-0.98068
WBP5	0.000173	2.51E-11	1.57E-11	0.422442	-0.92609	-1.34853
WDR54	0.000559	1.21E-05	4.23E-09	0.214972	-0.35338	-0.56836
WDYHV1	0.107949	2.15E-06	1.58E-08	0.132355	-0.41117	-0.54352
WFDC1	0.011247	0.000121	5.44E-08	0.333503	-0.58588	-0.91938
WIPF1	9.96E-10	5.79E-06	1.58E-11	0.449554	-0.42483	-0.87439
WIPI1	0.054414	1.73E-06	1.87E-09	0.1742	-0.47473	-0.64893
WISP1	0.001799	2.15E-06	1.72E-10	0.198654	-0.44553	-0.64419
WLS	0.004031	0.047733	1.28E-05	0.347803	-0.24936	-0.59716
WSB2	7.94E-05	2.15E-08	3.07E-11	0.247942	-0.34303	-0.59097
WWC3	1.55E-06	5.10E-07	2.96E-11	0.29453	-0.33428	-0.62881
XAF1	0.010243	0.901448	0.00448	0.426722	-0.08205	-0.50877
XCL1	3.58E-06	0.0009	8.19E-09	0.435839	-0.42802	-0.86385
XCL2	4.58E-06	0.000153	7.70E-10	0.616181	-0.5972	-1.21338
YAP1	2.14E-05	2.58E-05	5.62E-11	0.363408	-0.31838	-0.68178
YWHAB	9.25E-06	4.53E-08	3.21E-11	0.247916	-0.28918	-0.5371
YWHAZ	8.67E-08	1.95E-09	1.57E-11	0.289858	-0.39566	-0.68552
ZAP70	9.13E-08	0.000236	1.88E-10	0.403813	-0.38873	-0.79254
ZBTB16	0.972676	0.002715	0.001261	-0.01505	0.674967	0.690019
ZC3H12D	1.68E-08	1.06E-05	2.87E-11	0.594376	-0.5771	-1.17147
ZCCHC6	0.97743	1.26E-07	1.90E-05	0.06556	0.525951	0.460391
ZDHHC11	0.807852	2.87E-06	1.06E-06	0.042264	0.639066	0.596802
ZDHHC11B	0.626408	8.10E-06	4.44E-06	0.078421	0.730268	0.651846
ZEB2	1.74E-06	8.10E-06	1.79E-10	0.278669	-0.25123	-0.5299
ZFP3	0.006128	0.000737	1.06E-07	0.302746	-0.39173	-0.69447
ZFPM2	6.55E-06	0.003375	9.05E-09	0.603282	-0.40607	-1.00935
ZG16	0.207421	3.29E-06	2.48E-08	-0.16987	0.717828	0.8877
ZG16B	0.789185	1.47E-05	1.27E-06	0.034225	-0.48731	-0.52153
ZGPAT	0.001461	2.35E-08	7.58E-11	-0.25546	0.748727	1.004183
ZKSCAN1	0.175754	3.53E-10	1.76E-08	-0.06054	0.62027	0.68081
ZMAT3	3.67E-07	0.013186	1.23E-09	0.700315	-0.30603	-1.00635
ZNF101	1.87E-07	6.47E-07	3.21E-11	0.232881	-0.27394	-0.50682
ZNF124	0.00047	0.00297	2.68E-09	0.32048	-0.33089	-0.65137
ZNF14	3.48E-06	7.10E-06	1.15E-10	0.303502	-0.35696	-0.66047
ZNF165	0.087816	3.45E-08	1.43E-08	0.09051	-0.58411	-0.67462
ZNF200	0.000183	1.28E-06	2.66E-10	0.221779	-0.28304	-0.50482
ZNF267	1.33E-08	5.97E-07	2.13E-11	0.531464	-0.51861	-1.05008
ZNF367	6.55E-06	6.97E-07	1.21E-10	0.561463	-0.87344	-1.43491
ZNF385B	0.644005	8.07E-07	5.20E-08	0.037618	0.614826	0.577207
ZNF395	0.05777	6.28E-11	5.62E-11	-0.11574	0.51227	0.628011
ZNF430	8.73E-09	5.51E-07	1.64E-11	0.28584	-0.30815	-0.59399
ZNF439	1.11E-06	0.000223	3.40E-10	0.353315	-0.33521	-0.68852
ZNF468	0.006252	6.97E-07	2.43E-10	0.336981	-0.60424	-0.94122
ZNF511	0.000173	2.25E-07	3.97E-10	-0.23341	0.454008	0.687416

ZNF532	0.006384	1.10E-06	5.72E-10	0.205966	-0.39634	-0.60231
ZNF83	8.48E-05	0.000115	3.58E-10	0.298887	-0.37033	-0.66922
ZNFX1	9.25E-06	3.33E-05	5.17E-11	0.325307	-0.36306	-0.68837
ZUFSP	0.000434	0.000277	1.23E-09	0.261239	-0.30606	-0.5673
ZWILCH	6.64E-05	1.10E-06	1.72E-10	0.257138	-0.42487	-0.68201
ZWINT	3.58E-06	3.39E-07	1.57E-11	0.907146	-1.12058	-2.02773
