# Supplementary material for: Transcriptome Classification Reveals Molecular Subgroups in Patients with Hepatitis B Virus
Source: Comput Math Methods Med. 2021 Mar 30;2021:5543747. doi: 10.1155/2021/5543747 (PMC8028738; doi:10.1155/2021/5543747)
Supplement: Supplementary 2 — Supplementary Table S2: the functional module-specific genes in CHB patients. [file 5543747.f2.pdf]

Table S2. The genes within the functional modules

Module	gene	ENTREZID
M1	A1CF	29974
M2	AACS	65985
M1	AADACP1	201651
M1	AADAT	51166
M2	AAED1	NA
M1	AASS	10157
M1	ABCA6	23460
M2	ABCB1	5243
M2	ABCB4	5244
M3	ABCC1	4363
M1	ABCG2	9429
M1	ABHD15	116236
M2	abParts	NA
M3	ABRACL	58527
M1	AC004538.	NA
M1	AC012065.	NA
M2	AC017002.	NA
M2	AC079767.	NA
M2	AC128677.	NA
M1	ACACB	32
M1	ACADL	33
M1	ACADS	35
M1	ACADSB	36
M2	ACE2	59272
M2	ACLY	47
M1	ACMSD	130013
M1	ACOT13	55856
M2	ACOT9	23597
M1	ACOX2	8309
M3	ACP5	54
M4	ACSL4	2182
M1	ACSM2A	123876
M1	ACSM2B	348158
M1	ACSM3	6296
M1	ACSM5	54988
M3	ACTB	60
M3	ACTG1	71
M3	ACYP1	97
M2	ADA	100
M2	ADAMDEC	27299
M2	ADAMTS2	9509
M2	ADAMTSL:	9719
M2	ADAP2	55803
M1	ADCY1	107
M1	ADCY10	55811
M3	ADCY7	113
M1	ADH6	130
M1	ADHFE1	137872
M3	ADIRF	10974
M1	ADORA2A	646023
M3	ADORA3	140
M1	ADRA1A	148
M2	ADRA2A	150
M2	ADRBK2	NA
M2	AEBP1	165
M4	AF070581	NA

M2	AGA	175
M1	AGBL2	79841
M1	AGL	178
M1	AGXT2	64902
M2	AIF1	199
M2	AIM2	9447
M3	AJUBA	84962
M3	AKIP1	56672
M2	AKR1B1	231
M2	AKR1B10	57016
M1	AKR1D1	6718
M1	ALAD	210
M1	ALDH1L1	10840
M1	ALDH6A1	4329
M2	ALDOA	226
M2	ALOX5	240
M2	ALOX5AP	241
M1	ALPK2	115701
M2	AMICA1	NA
M3	AMIGO2	347902
M1	AMN	81693
M1	AMT	275
M2	ANGPTL2	23452
M2	ANKRD22	118932
M2	ANKRD29	147463
M1	ANKRD35	148741
M2	ANLN	54443
M2	ANXA1	301
M1	ANXA10	11199
M3	ANXA13	312
M2	ANXA2	302
M2	ANXA2P2	304
M2	ANXA3	306
M2	ANXA4	307
M2	ANXA5	308
M3	AOAH	313
M1	AOX1	316
M1	AP000253.	NA
M2	AP1S2	8905
M2	APCDD1	147495
M2	APH1B	83464
M1	APOA5	116519
M2	APOBEC3A	200315
M2	APOBEC3A	1.01E+08
M2	APOBEC3E	9582
M2	APOBEC3C	60489
M1	APOF	319
M2	APOL3	80833
M2	AQP1	358
M1	AR	367
M2	ARF4	378
M1	ARHGAP1(	79658
M2	ARHGAP18	93663
M2	ARHGAP25	9938
M2	ARHGAP4	393
M2	ARHGAP9	64333
M2	ARHGDIB	397
M3	ARHGEF18	23370

M1	ARHGEF26	26084
M2	ARHGEF3	50650
M3	ARHGEF6	9459
M2	ARL4C	10123
M3	ARL6IP5	10550
M3	ARMCX1	51309
M2	ARMCX2	9823
M3	ARMCX3	51566
M1	ARNTL	406
M2	ARNTL2	56938
M2	ARPC1B	10095
M2	ARPC2	10109
M2	ARPC3	10094
M2	ARRDC2	27106
M1	ARRDC3	57561
M2	ARRDC4	91947
M1	ARSE	NA
M4	ARSJ	79642
M1	ASB9	140462
M3	ASCC3	10973
M4	ASNS	440
M1	ASPA	443
M1	ASPG	374569
M2	ASPHD2	57168
M2	ASPM	259266
M3	ASPN	54829
M3	ASRGL1	80150
M2	ATAD2	29028
M1	ATF5	22809
M1	ATF7IP2	80063
M3	ATP10D	57205
M2	ATP6V0B	533
M2	ATP6V1F	9296
M2	ATP8B2	57198
M3	ATP8B4	79895
M2	AURKA	6790
M1	AVPR1A	552
M1	AX747132	NA
M1	AX747135	NA
M1	AZGP1	563
M1	B3GAT1	27087
M1	BAAT	570
M2	BACE2	25825
M3	BAG3	9531
M2	BAMBI	25805
M2	BARD1	580
M2	BATF	10538
M2	BATF2	116071
M3	BAX	581
M3	BAZ1A	11177
M1	BBOX1	8424
M2	BBS12	166379
M2	BBS7	55212
M4	BC034319	NA
M1	BC038731	NA
M1	BC040327	NA
M2	BCAT1	586
M1	BCHE	590

M2	BCL11B	64919
M2	BCL2A1	597
M3	BEX1	55859
M2	BEX2	84707
M3	BEX4	56271
M3	BEX5	340542
M2	BICC1	80114
M2	BIN2	51411
M2	BIRC3	330
M2	BLM	641
M3	BLOC1S2	282991
M2	BLVRA	644
M2	BMS1P20	96610
M2	BORA	79866
M2	BTG2	7832
M3	BTK	695
M2	BTLA	151888
M2	BTN3A2	11118
M2	BTN3A3	10384
M2	BUB1B	701
M2	C10orf54	NA
M1	C11orf71	54494
M2	C11orf80	79703
M2	C11orf96	387763
M2	C12orf4	57102
M2	C12orf5	NA
M2	C12orf75	387882
M1	C14orf132	56967
M1	C15orf43	NA
M2	C15orf48	84419
M2	C15orf52	NA
M2	C16orf54	283897
M1	C17orf67	339210
M4	C1orf106	NA
M2	C1orf162	128346
M2	C1orf198	84886
M3	C1orf216	127703
M2	C1orf54	79630
M2	C1QA	712
M2	C1QB	713
M2	C1QC	714
M1	C1R	715
M3	C3AR1	719
M1	C3P1	388503
M2	C4orf48	401115
M2	C5AR1	728
M1	C5orf27	NA
M1	C6	729
M2	C7	730
M2	C7orf31	136895
M1	C7orf55	NA
M1	C8A	731
M1	C8B	732
M1	C8orf46	NA
M4	C9orf152	401546
M1	CACNA1H	8912
M2	CALHM2	51063
M2	CAP1	10487

M2	CAP2	10486
M4	CAPG	822
M2	CAPN2	824
M1	CAPN3	825
M1	CAPN5	726
M3	CAPNS1	826
M2	CARD16	114769
M2	CARD6	84674
M1	CASC10	NA
M2	CASP1	834
M2	CASP4	837
M3	CAV2	858
M1	CBFA2T3	863
M1	CBLN4	140689
M1	CBS	875
M4	CCDC102E	79839
M2	CCDC109E	NA
M2	CCDC146	57639
M2	CCDC3	83643
M2	CCDC80	151887
M1	CCL14	6358
M2	CCL18	6362
M2	CCL19	6363
M2	CCL2	6347
M4	CCL20	6364
M2	CCL21	6366
M2	CCL3	6348
M2	CCL3L1	6349
M2	CCL3L3	414062
M2	CCL4	6351
M2	CCL5	6352
M2	CCL8	6355
M2	CCNA2	890
M2	CCNB1	891
M3	CCND1	595
M2	CCNE2	9134
M2	CCR1	1230
M2	CCR2	729230
M2	CCR5	1234
M2	CCR7	1236
M2	CCRL2	9034
M1	CCS	9973
M3	CCT6A	908
M2	CD163	9332
M2	CD163L1	283316
M2	CD1E	913
M3	CD2	914
M2	CD200	4345
M2	CD226	10666
M2	CD24	1E+08
M3	CD247	919
M2	CD27	939
M2	CD300LF	146722
M2	CD38	952
M2	CD3D	915
M2	CD3G	917
M2	CD44	960
M2	CD48	962

M2	CD52	1043
M2	CD53	963
M2	CD58	965
M2	CD5L	922
M2	CD69	969
M2	CD72	971
M2	CD74	972
M2	CD83	9308
M2	CD86	942
M2	CD8A	925
M2	CD96	10225
M2	CD97	NA
M1	CDA	978
M2	CDC20	991
M2	CDC25B	994
M2	CDC42EP3	10602
M2	CDC42EP5	148170
M2	CDC42SE1	56882
M2	CDC42SE2	56990
M2	CDC45	8318
M2	CDC6	990
M2	CDC7	8317
M2	CDCA3	83461
M2	CDCA5	113130
M3	CDCA7L	55536
M2	CDH11	1009
M3	CDHR2	54825
M1	CDHR5	53841
M2	CDK1	983
M1	CDK3	1018
M2	CDKN1A	1026
M2	CDKN1C	1028
M2	CDKN2C	1031
M2	CDKN3	1033
M1	CDO1	1036
M2	CECR1	NA
M1	CECR2	27443
M3	CELF2	10659
M2	CENPA	1058
M2	CENPE	1062
M4	CENPH	64946
M2	CENPK	64105
M2	CENPM	79019
M2	CENPU	79682
M2	CENPW	387103
M2	CEP55	55165
M1	CES3	23491
M1	CES4A	283848
M1	CES5A	221223
M2	CFD	1675
M1	CFHR3	10878
M1	CFHR4	10877
M1	CFHR5	81494
M4	CFTR	1080
M1	CGN	57530
M2	CH17-132	NA
M2	CH25H	9023
M1	CHAD	1101

M2	CHEK2	11200
M4	CHI3L1	1116
M2	CHMP3	51652
M4	CHRDL1	91851
M2	CHST11	50515
M2	CHST4	10164
M2	CHST9	83539
M3	CHSY1	22856
M1	CIDEB	27141
M2	CKAP2	26586
M2	CKLF	51192
M2	CKLF-CMT	1.01E+08
M4	CKMT2	1160
M2	CKS2	1164
M2	CLDN10	9071
M2	CLDN11	5010
M1	CLDN14	23562
M2	CLDN7	1366
M2	CLEC11A	6320
M2	CLEC2B	9976
M2	CLEC2D	29121
M2	CLEC4A	50856
M2	CLEC7A	64581
M2	CLIC1	1192
M3	CLIC2	1193
M2	CLIC3	9022
M2	CLIC6	54102
M2	CLIP4	79745
M1	CMBL	134147
M2	CMPK2	129607
M2	CMTM7	112616
M1	CMTM8	152189
M1	CNDP1	84735
M1	CNGA1	1259
M3	CNOT6	57472
M3	CNRIP1	25927
M1	COBLL1	22837
M4	COL15A1	1306
M2	COL16A1	1307
M2	COL1A1	1277
M2	COL1A2	1278
M2	COL3A1	1281
M2	COL4A1	1282
M2	COL4A2	1284
M2	COL4A4	1286
M2	COL5A1	1289
M2	COL5A2	1290
M2	COL6A2	1292
M2	COL6A3	1293
M2	COMMD8	54951
M1	COQ10A	93058
M2	CORO1A	11151
M2	CORO1C	23603
M2	COTL1	23406
M4	COX7A1	1346
M4	CPA3	1359
M1	CPS1-IT1	29034
M2	CPVL	54504

M3	CRIM1	51232
M2	CRIP1	1396
M2	CRISPLD2	83716
M3	CRLF3	51379
M2	CRP	1401
M2	CRTAM	56253
M4	CRYAB	1410
M3	CRYBG3	131544
M2	CSF1R	1436
M3	CSF2RB	1439
M2	CST7	8530
M1	CTD-2284.	NA
M1	CTD-3080	1.02E+08
M1	CTD-3193	NA
M2	CTGF	NA
M4	CTHRC1	115908
M1	CTNNA3	29119
M3	CTR9	9646
M3	CTSA	5476
M2	CTSC	1075
M3	CTSD	1509
M2	CTSK	1513
M2	CTSS	1520
M2	CTTNBP2N	55917
M1	CUX2	23316
M4	CXCL1	2919
M2	CXCL10	3627
M2	CXCL11	6373
M3	CXCL12	6387
M2	CXCL13	10563
M2	CXCL16	58191
M1	CXCL2	2920
M2	CXCL6	6372
M4	CXCL8	3576
M2	CXCL9	4283
M2	CXCR4	7852
M2	CXCR6	10663
M3	CXorf23	NA
M3	CXorf38	159013
M2	CYAT1	1E+08
M2	CYBRD1	79901
M3	CYP11A1	1583
M1	CYP1A1	1543
M1	CYP1A2	1544
M1	CYP26A1	1592
M1	CYP2A13	1553
M1	CYP2A6	1548
M1	CYP2A7	1549
M1	CYP2C19	1557
M1	CYP2D7P	NA
M1	CYP39A1	51302
M1	CYP3A4	1576
M1	CYP3A43	64816
M4	CYP3A7	1551
M1	CYP4A11	1579
M1	CYP4A22	284541
M1	CYP4F12	66002
M1	CYP4F3	4051

M1	CYP7A1	1581
M2	CYR61	NA
M2	CYS1	192668
M2	CYSLTR1	10800
M2	CYTIP	9595
M2	DACT1	51339
M1	DAK	NA
M3	DBF4	10926
M1	DBH-AS1	138948
M2	DBN1	1627
M2	DBNDD2	55861
M1	DCDC1	341019
M2	DCDC2	51473
M1	DCDC5	NA
M2	DCK	1633
M2	DCN	1634
M2	DDB2	1643
M2	DDIAS	220042
M2	DDX26B	NA
M2	DDX39A	10212
M3	DDX60	55601
M2	DEFB1	1672
M2	DENND1C	79958
M3	DENND2D	79961
M3	DENND6A	201627
M2	DEPDC1	55635
M4	DEPDC1B	55789
M3	DFNA5	NA
M1	DGAT2	84649
M1	DHRS1	115817
M1	DHRS2	10202
M1	DHRS4-AS	55449
M2	DHRS9	10170
M1	DIRAS3	9077
M2	DKFZp667.	NA
M2	DKK3	27122
M1	DLEU1	10301
M2	DLGAP5	9787
M1	DMGDH	29958
M3	DMXL2	23312
M1	DNAJC12	56521
M1	DNAJC25	548645
M1	DNAJC25-	552891
M2	DNAJC5B	85479
M1	DNM1	1759
M2	DNM3OS	1.01E+08
M3	DNMT1	1786
M1	DNMT3L	29947
M2	DOCK10	55619
M2	DOCK11	139818
M2	DOCK2	1794
M3	DOCK8	81704
M2	DOK2	9046
M2	DOK3	79930
M2	DPT	1805
M3	DPYSL2	1808
M2	DRAM1	55332
M3	DRAM2	128338

M1	DSG1	1828
M2	DSN1	79980
M2	DTL	51514
M2	DUSP2	1844
M2	DYNLT1	6993
M2	DZIP1	22873
M2	E2F3	1871
M4	E2F8	79733
M2	EBI3	10148
M1	EBPL	84650
M1	ECHDC2	55268
M2	ECT2	1894
M2	EDNRB	1910
M3	EFCAB7	84455
M2	EFEMP1	2202
M2	EFEMP2	30008
M1	EFHD1	80303
M1	EFNA1	1942
M2	EGR1	1958
M2	EGR2	1959
M2	EGR3	1960
M2	ELF3	1999
M2	ELF4	2000
M2	ELOVL1	64834
M2	ELOVL2	54898
M2	ELOVL7	79993
M2	EMILIN1	11117
M2	EMP3	2014
M2	EMR1	NA
M2	EMR2	NA
M2	ENDOD1	23052
M1	ENHO	375704
M1	ENPP1	5167
M2	ENPP2	5168
M4	ENPP5	59084
M3	ENPP7	339221
M2	ENTPD1	953
M3	EOGT	285203
M3	EOMES	8320
M2	EPB41L2	2037
M2	EPB41L3	23136
M4	EPCAM	4072
M2	EPDR1	54749
M2	EPHA3	2042
M1	EPHB1	2047
M1	EPHX2	2053
M2	EPSTI1	94240
M2	ERAP2	64167
M3	ERI1	90459
M2	ERICH5	203111
M3	ERMP1	79956
M2	ERP27	121506
M1	ERRFI1	54206
M4	ESRP1	54845
M1	ETNK2	55224
M2	ETV7	51513
M2	EVI2A	2123
M3	EVI2B	2124

M3	EXOC1	55763
M1	EXPH5	23086
M2	EZH2	2146
M1	F11	2160
M2	F13A1	2162
M3	F2RL1	2150
M2	F3	2152
M4	FABP3	2170
M4	FABP4	2167
M2	FABP5	2171
M3	FADS1	3992
M2	FAIM	55179
M2	FAIM3	NA
M2	FAM102B	284611
M4	FAM111B	374393
M3	FAM122B	159090
M4	FAM124B	79843
M3	FAM127A	NA
M2	FAM129A	NA
M1	FAM134B	NA
M2	FAM150B	NA
M1	FAM151A	338094
M2	FAM169A	26049
M1	FAM198A	NA
M2	FAM213B	NA
M2	FAM26F	NA
M4	FAM3B	54097
M3	FAM46C	NA
M2	FAM49A	81553
M2	FAM57A	NA
M2	FAM60A	NA
M2	FAM65B	NA
M2	FAM72A	729533
M2	FAM72B	653820
M2	FAM72C	554282
M2	FAM72D	728833
M2	FAM78A	286336
M1	FAM9B	171483
M2	FANCI	55215
M4	FAP	2191
M2	FAR1	84188
M3	FAS	355
M2	FAT1	2195
M2	FBLN5	10516
M2	FBN1	2200
M3	FBXO6	26270
M3	FCAMR	83953
M2	FCER1G	2207
M2	FCGR1A	2209
M2	FCGR1B	2210
M2	FCGR1C	NA
M3	FCGR3A	2214
M3	FCGR3B	2215
M2	FCN1	2219
M2	FCRL3	115352
M3	FDXR	2232
M2	FEN1	2237
M3	FERMT3	83706

M2	FGF13	2258
M1	FGF14-AS2	283481
M2	FGL2	10875
M2	FGR	2268
M2	FHL2	2274
M2	FHOD1	29109
M3	FIG4	9896
M3	FIGNL1	63979
M2	FILIP1L	11259
M1	FITM1	161247
M2	FJX1	24147
M2	FKBP11	51303
M4	FKBP1A-SI	1.01E+08
M2	FKBP1B	2281
M2	FLJ12120	NA
M1	FLJ22763	NA
M2	FLJ32255	643977
M2	FLNA	2316
M3	FLRT3	23767
M2	FLVCR1	28982
M2	FMO1	2326
M2	FMO2	2327
M1	FMO5	2330
M2	FMOD	2331
M4	FNDC1	84624
M1	FNIP2	57600
M1	FOLH1	2346
M1	FOLH1B	219595
M2	FOS	2353
M2	FOXM1	2305
M4	FOXQ1	94234
M2	FPR3	2359
M2	FRMD3	257019
M2	FRMD6	122786
M2	FSTL3	10272
M1	FTCD	10841
M3	FUNDC1	139341
M2	FUT4	2526
M2	FUT8	2530
M1	FXYD1	5348
M2	FXYD2	486
M2	FXYD5	53827
M2	FXYD6-FX	1.01E+08
M2	FYB	NA
M2	FZD6	8323
M2	FZD7	8324
M1	G6PC	2538
M3	GABBR1	2550
M2	GABRE	2564
M4	GABRP	2568
M1	GALK1	2584
M3	GALNT7	51809
M2	GAPT	202309
M1	GAS2	2620
M2	GAS2L3	283431
M1	GBA3	57733
M3	GBAS	NA
M2	GBP1	2633

M2	GBP2	2634
M3	GBP3	2635
M2	GBP4	115361
M2	GBP5	115362
M2	GCA	25801
M1	GCAT	23464
M1	GCDH	2639
M1	GCHFR	2644
M4	GCK	2645
M1	GCKR	2646
M2	GDF15	9518
M2	GEM	2669
M1	GFRA1	2674
M1	GGACT	87769
M2	GGTA1P	2681
M3	GIMAP2	26157
M3	GIMAP4	55303
M3	GIMAP6	474344
M3	GIMAP7	168537
M2	GINS1	9837
M2	GINS3	64785
M2	GJA1	2697
M2	GK	2710
M2	GK3P	2713
M2	GLA	2717
M2	GLIPR1	11010
M2	GLIPR2	152007
M2	GLIS2	84662
M4	GLIS3	169792
M1	GLS2	27165
M2	GLT8D2	83468
M1	GLYAT	10249
M1	GLYCTK	132158
M2	GMFB	2764
M2	GMFG	9535
M2	GMNN	51053
M2	GMPR	2766
M1	GNE	10020
M1	GNMT	27232
M3	GNPDA1	10007
M3	GNPDA2	132789
M3	GNS	2799
M2	GOLM1	51280
M2	GOLT1B	51026
M3	GORAB	92344
M1	GPAM	57678
M4	GPC3	2719
M2	GPC4	2239
M2	GPCPD1	56261
M1	GPER1	2852
M2	GPNMB	10457
M2	GPR124	NA
M1	GPR125	NA
M3	GPR137B	7107
M2	GPR137C	283554
M2	GPR160	26996
M2	GPR171	29909
M2	GPR18	2841

M3	GPR34	2857
M2	GPR56	NA
M3	GPR65	8477
M2	GPR82	27197
M1	GPR88	54112
M2	GPRC5B	51704
M2	GPRIN3	285513
M2	GPX2	2877
M3	GPX3	2878
M2	GPX7	2882
M4	GPX8	493869
M4	GRAMD1B	57476
M1	GRAMD1C	54762
M1	GRAMD3	NA
M1	GRB14	2888
M1	GREM1	26585
M1	GREM2	64388
M4	GRHL2	79977
M3	GRN	2896
M1	GRTP1	79774
M1	GSDMB	55876
M3	GSKIP	51527
M2	GSTP1	2950
M2	GUCY1A3	NA
M2	GYG1	2992
M2	GYPC	2995
M1	GYS2	2998
M2	GZMA	3001
M2	GZMB	3002
M2	GZMH	2999
M3	GZMK	3003
M3	H19	283120
M2	H2AFY2	NA
M2	H2AFZ	NA
M1	HAAO	23498
M1	HAGH	3029
M1	HAO2	51179
M1	HAPLN4	404037
M3	HAUS3	79441
M1	HBA1	3039
M1	HBA2	3040
M1	HBB	3043
M2	HCAR3	8843
M2	HCK	3055
M3	HCLS1	3059
M2	HCP5	10866
M2	HCST	10870
M1	HDHD3	81932
M2	HEG1	57493
M2	HELLS	3070
M2	HENMT1	113802
M1	HEPACAM	220296
M2	HEPH	9843
M1	HEPN1	641654
M2	HES4	57801
M2	HGF	3082
M1	HGFAC	3083
M3	HIF1A	3091

M2	HJURP	55355
M2	HK1	3098
M2	HK3	3101
M2	HKDC1	80201
M2	HLA-A	3105
M2	HLA-B	3106
M2	HLA-C	3107
M2	HLA-DMA	3108
M2	HLA-DMB	3109
M2	HLA-DPA1	3113
M2	HLA-DPB1	3115
M2	HLA-DPB2	3116
M2	HLA-DQA[illegible]	3117
M3	HLA-DQA[illegible]	3118
M2	HLA-DQB[illegible]	3119
M2	HLA-DRA	3122
M4	HLA-DRB1	3123
M4	HLA-DRB[illegible]	3125
M2	HLA-DRB4	3126
M4	HLA-DRB[illegible]	3127
M2	HLA-DRB[illegible]	3128
M2	HLA-E	3133
M2	HLA-F	3134
M2	HLA-G	3135
M2	HLA-J	3137
M1	HLX	3142
M2	HMCN1	83872
M2	HMGB2	3148
M1	HMGCS2	3158
M2	HMGN1	3150
M2	HMGN4	10473
M2	HMMR	3161
M3	HMOX1	3162
M2	HN1	NA
M1	HNF4A-A[illegible]	1.02E+08
M1	HOGA1	112817
M1	HOMER2	9455
M3	HOTS	1.03E+08
M1	HPN	3249
M1	HPR	3250
M3	HPS5	11234
M3	HPSE	10855
M4	HRCT1	646962
M4	HS3ST2	9956
M1	HSBP1L1	440498
M1	HSD11B1	3290
M1	HSD17B14	51171
M2	HSPA2	3306
M2	HSPA4L	22824
M2	HSPA6	3310
M1	HSPB9	94086
M3	HSPBAP1	79663
M2	HTR2B	3357
M1	IAPP	3375
M2	IARS	NA
M2	ICAM1	3383
M2	ICAM2	3384
M2	ICOS	29851

M3	ID1	3397
M2	ID3	3399
M2	ID4	3400
M2	IDO1	3620
M4	IER3	8870
M3	IER5	51278
M2	IER5L	389792
M3	IFI16	3428
M2	IFI27	3429
M2	IFI30	10437
M2	IFI35	3430
M2	IFI44	10561
M2	IFI44L	10964
M2	IFI6	2537
M2	IFIT3	3437
M2	IFIT5	24138
M2	IFITM1	8519
M2	IFNG	3458
M2	IFNGR1	3459
M3	IFNGR2	3460
M1	IGFALS	3483
M2	IGFBP6	3489
M2	IGFBP7	3490
M2	IGH	3492
M2	IGHA1	3493
M2	IGHA2	3494
M2	IGHD	3495
M2	IGHG2	3501
M2	IGHG3	3502
M2	IGHG4	3503
M2	IGHM	3507
M2	IGHV3-23	28442
M2	IGHV4-31	28396
M2	IGJ	NA
M2	IGK	50802
M2	IGKC	3514
M2	IGKV1-17	28937
M2	IGKV1-37	28931
M2	IGKV1D-3[illegible]	28894
M2	IGKV1OR-	NA
M2	IGKV1OR1	642424
M2	IGKV1OR2	28862
M2	IGKV1OR2	1.06E+08
M2	IGKV2-28	28921
M2	IGKV2D-2[illegible]	28883
M2	IGKV4-1	28908
M2	IGLC1	3537
M2	IGLJ2	28832
M2	IGLJ3	28831
M2	IGLL3P	91353
M2	IGLL5	1E+08
M2	IGLV@	3546
M2	IGLV1-36	28826
M2	IGLV1-40	28825
M2	IGLV1-44	28823
M2	IGLV1-50	28821
M2	IGLV2-14	28815
M2	IGLV3-1	28809

M2	IGLV3-10	28803
M2	IGLV3-19	28797
M2	IGLV3-25	28793
M4	IGLV9-49	28773
M2	IGSF6	10261
M1	IGSF9	57549
M2	IKBIP	121457
M2	IKZF3	22806
M2	IL10RA	3587
M2	IL15	3600
M2	IL15RA	3601
M2	IL18	3606
M2	IL1B	3553
M1	IL1RAP	3556
M4	IL20RB	53833
M2	IL21R	50615
M2	IL2RB	3560
M2	IL2RG	3561
M3	IL32	9235
M1	IL6R	3570
M2	IL7	3574
M2	IL7R	3575
M2	INMT	11185
M2	INPP5F	22876
M2	IPCEF1	26034
M3	IQGAP1	8826
M2	IRF1	3659
M2	IRF8	3394
M1	IRS1	3667
M1	IRS2	8660
M2	ISG15	9636
M2	ISG20	3669
M2	ISLR	3671
M2	ITGA2	3673
M2	ITGA4	3676
M2	ITGA6	3655
M2	ITGAL	3683
M2	ITGAM	3684
M2	ITGAV	3685
M2	ITGB2	3689
M2	ITGB2-AS1	1.01E+08
M2	ITGB3BP	23421
M2	ITGBL1	9358
M1	ITIH1	3697
M2	ITIH5	80760
M2	ITK	3702
M4	ITLN1	55600
M2	ITM2A	9452
M2	JAG1	182
M2	JAK2	3717
M3	JAZF1	221895
M2	JUN	3725
M2	JUNB	3726
M2	KAL1	NA
M1	KANK4	163782
M1	KBTBD11	9920
M2	KBTBD8	84541
M2	KCNJ10	3766

M2	KCNJ16	3773
M2	KCNJ2	3759
M1	KCNK5	8645
M1	KCNN2	3781
M3	KCNS3	3790
M3	KCTD12	115207
M3	KCTD9	54793
M2	KDELR3	11015
M1	KHK	3795
M2	KIAA0101	NA
M3	KIAA0226L	NA
M4	KIAA1211	NA
M3	KIAA1598	NA
M1	KIAA1804	NA
M2	KIF11	3832
M2	KIF14	9928
M2	KIF15	56992
M3	KIF16B	55614
M2	KIF18A	81930
M2	KIF18B	146909
M2	KIF20A	10112
M2	KIF21B	23046
M2	KIF2A	3796
M2	KIF4A	24137
M2	KIF5B	3799
M1	KLB	152831
M3	KLF6	1316
M2	KLHL29	114818
M2	KLHL6	89857
M1	KLKB1	3818
M2	KLRB1	3820
M2	KLRC1	3821
M2	KLRC2	3822
M2	KLRC4-KLI	1.01E+08
M2	KLRK1	22914
M2	KNTC1	9735
M2	KPNA2	3838
M2	KRT18	3875
M2	KRT19	3880
M2	KRT222	125113
M4	KRT23	25984
M3	KRT8	3856
M2	LAIR1	3903
M2	LAIR2	3904
M2	LAMA2	3908
M3	LAMB1	3912
M2	LAMP3	27074
M2	LAPTM5	7805
M2	LAX1	54900
M2	LBH	81606
M1	LCAT	3931
M2	LCK	3932
M2	LCN2	3934
M2	LCP1	3936
M2	LCP2	3937
M2	LDHB	3945
M1	LDHD	197257
M2	LDLRAD3	143458

M2	LDOC1	23641
M2	LEPROTL1	23484
M2	LGALS2	3957
M2	LGALS3	3958
M3	LGALS3BP	3959
M2	LGALS4	3960
M2	LGALS9	3965
M3	LGMN	5641
M1	LGSN	51557
M2	LHFP	NA
M2	LHFPL2	10184
M4	LIF	3976
M2	LILRB1	10859
M2	LILRB2	10288
M3	LIMA1	51474
M1	LIME1	54923
M2	LINC00152	NA
M1	LINC00844	1.01E+08
M2	LINC00924	145820
M1	LINC00939	400084
M1	LINC01018	255167
M1	LINC01093	1.01E+08
M2	LINC01094	1.01E+08
M1	LINC01127	1.01E+08
M4	LINC01279	1.01E+08
M2	LINC01420	NA
M1	LIPC	3990
M1	LIPG	9388
M2	LMNB1	4001
M1	LNP1	348801
M1	LOC10013	NA
M2	LOC10013	NA
M2	LOC10013	NA
M2	LOC10028	NA
M1	LOC10028	NA
M1	LOC10028	NA
M2	LOC10050	1.01E+08
M2	LOC10050	NA
M1	LOC10050	1.01E+08
M2	LOC10050	1.01E+08
M2	LOC10050	1.01E+08
M2	LOC10050	1.01E+08
M1	LOC10050	1.01E+08
M2	LOC10050	NA
M2	LOC10050	NA
M1	LOC10099	1.01E+08
M4	LOC10099	NA
M1	LOC10106	NA
M2	LOC10106	NA
M4	LOC10192	1.02E+08
M2	LOC10192	1.02E+08
M2	LOC10192	1.02E+08
M1	LOC10192	NA
M1	LOC10192	NA
M2	LOC10192	NA
M1	LOC10192	1.02E+08
M3	LOC10192	NA
M1	LOC10192	NA

M1	LOC10192	1.02E+08
M2	LOC10192	1.02E+08
M2	LOC10192	1.02E+08
M1	LOC10192	1.02E+08
M3	LOC10192	1.02E+08
M2	LOC10192	NA
M2	LOC10192	NA
M2	LOC10192	NA
M1	LOC10192	NA
M4	LOC10192	NA
M3	LOC10192	NA
M1	LOC10192	1.02E+08
M2	LOC10193	NA
M1	LOC10272	NA
M2	LOC10272	1.03E+08
M1	LOC10272	NA
M2	LOC10272	NA
M1	LOC14583	NA
M1	LOC14970	NA
M1	LOC15727	157273
M2	LOC15840	NA
M3	LOC20202	NA
M1	LOC28466	NA
M1	LOC28518	NA
M2	LOC28562	NA
M2	LOC37444	374443
M1	LOC38983	389831
M2	LOC72839	728392
M2	LOC72871	728715
M4	LOC72968	NA
M1	LOC73010	730101
M4	LOXL1	4016
M4	LOXL4	84171
M1	LPA	4018
M1	LPAL2	80350
M2	LPAR1	1902
M2	LPAR5	57121
M2	LPCAT1	79888
M2	LRMP	4033
M4	LRRC1	55227
M1	LRRC19	64922
M3	LRRC25	126364
M2	LRRC32	2615
M3	LSP1	4046
M2	LST1	7940
M2	LTBP2	4053
M2	LUM	4060
M2	LXN	56925
M3	LY75	4065
M2	LY86	9450
M2	LY9	4063
M2	LY96	23643
M2	LYN	4067
M2	LYSMD2	256586
M1	LYVE1	10894
M2	LYZ	4069
M2	MAB21L2	10586
M2	MAD2L1	4085

M4	MAFF	23764
M1	MAGI2-AS	1.01E+08
M1	MAMDC4	158056
M2	MAML2	84441
M2	MAN2B1	4125
M3	MANF	7873
M1	MAOA	4128
M2	MAP1LC3E	81631
M2	MAP2	4133
M1	MAP2K1	5604
M3	MAP3K1	4214
M2	MAP3K8	1326
M2	MAP4K1	11184
M3	MAP7D1	55700
M4	MAPK13	5603
M2	MAPRE1	22919
M1	1-Mar	64757
M1	2-Mar	54996
M2	1-Mar	NA
M2	MARCKS	4082
M1	MAT1A	4143
M1	MBL1P	8512
M3	MBOAT1	154141
M2	MCAM	4162
M1	MCEE	84693
M4	MCM10	55388
M2	MCM2	4171
M2	MCM3	4172
M2	MCM4	4173
M2	MCM5	4174
M2	MCM6	4175
M2	MCM7	4176
M2	MCOLN2	255231
M2	MCTP1	79772
M2	MDFIC	29969
M1	MDN1	23195
M3	ME1	4199
M2	ME2	4200
M2	MELK	9833
M4	MEOX1	4222
M2	METRNL	284207
M2	MFAP4	4239
M2	MFSD1	64747
M2	MFSD6	54842
M1	MGMT	4255
M2	MGP	4256
M2	MICAL1	64780
M3	MICALL1	85377
M3	MICB	4277
M2	MID1IP1	58526
M2	MIR155	406947
M2	MIR155HG	114614
M3	MIR1908	1E+08
M2	MIR21	406991
M2	MIR224	407009
M2	MIR34A	407040
M2	MIR4435-:	NA
M2	MIR452	574412

M2	MIR6734	1.02E+08
M3	MIR675	1E+08
M2	MIR6756	1.02E+08
M1	MIR6778	1.02E+08
M2	MIR7703	1.02E+08
M2	MIR8071-1	1.02E+08
M2	MIR8071-2	1.02E+08
M2	MKI67	4288
M2	MLKL	197259
M2	MLLT11	10962
M1	MLPH	79083
M1	MLXIPL	51085
M1	MMAB	326625
M2	MMD	23531
M1	MME	4311
M3	MMGT1	93380
M4	MMP7	4316
M2	MMP9	4318
M2	MND1	84057
M2	MNDA	4332
M4	MNS1	55329
M2	MOB1A	55233
M1	MOGAT1	116255
M1	MOGAT2	80168
M2	MORC4	79710
M4	MOXD1	26002
M1	MPDZ	8777
M3	MPEG1	219972
M1	MPND	84954
M1	MPPED1	758
M2	MPV17	4358
M3	MR1	3140
M1	MROH2A	339766
M2	MS4A1	931
M2	MS4A4A	51338
M2	MS4A7	58475
M3	MSH2	4436
M2	MSR1	4481
M1	MT1F	4494
M1	MT1G	4495
M1	MT1H	4496
M1	MT1M	4499
M1	MT1X	4501
M2	MTCL1	23255
M2	MTHFD2	10797
M2	MTMR11	10903
M2	MTMR2	8898
M2	MUM1L1	NA
M3	MVP	9961
M2	MX1	4599
M2	MX2	4600
M1	MXI1	4601
M3	MXRA5	25878
M2	MYC	4609
M2	MYL12B	103910
M1	MYO16	23026
M2	MYO1F	4542
M2	MYO1G	64005

M2	MYO5A	4644
M2	MYOF	26509
M1	MYOM1	8736
M2	MZB1	51237
M2	NAA40	79829
M2	NABP1	64859
M2	NAGK	55577
M1	NAGS	162417
M2	NALCN	259232
M3	NAP1L1	4673
M3	NAP1L3	4675
M2	NAPSB	256236
M2	NARR	NA
M2	NBEA	26960
M3	NCAPD2	9918
M2	NCAPG	64151
M3	NCEH1	57552
M2	NCF1	653361
M2	NCF1B	654816
M2	NCF1C	654817
M2	NCF2	4688
M3	NCF4	4689
M2	NCK2	8440
M2	NCKAP1L	3071
M2	NDC80	10403
M2	NDN	4692
M4	NEB	4703
M2	NEDD9	4739
M2	NELL2	4753
M2	NETO2	81831
M1	NEU4	129807
M3	NEURL1B	54492
M2	NEURL3	93082
M4	NEXN	91624
M4	NFE2L3	9603
M2	NFKBIE	4794
M1	NFKBIZ	64332
M2	NKG7	4818
M3	NLRC3	197358
M2	NLRC5	84166
M2	NME5	8382
M2	NMI	9111
M1	NMRK1	54981
M3	NOD2	64127
M1	NOL4	8715
M2	NPC2	10577
M2	NPNT	255743
M4	NPTX2	4885
M3	NPW	283869
M1	NPY6R	4888
M4	NQO1	1728
M1	NR1I2	8856
M1	NR1I3	9970
M1	NR3C2	4306
M2	NRROS	375387
M1	NSUN6	221078
M2	NT5C3A	51251
M1	NTHL1	4913

M2	NTS	4922
M3	NUAK2	81788
M2	NUF2	83540
M1	NUPR1	26471
M2	NUSAP1	51203
M2	OAS1	4938
M2	OAS2	4939
M1	OAT	4942
M2	ODC1	4953
M2	OGFRL1	79627
M2	OIP5	11339
M3	OLFML1	283298
M2	OLFML2B	25903
M3	OLFML3	56944
M2	ORC6	23594
M2	OSBPL3	26031
M1	OSBPL6	114880
M2	OSCAR	126014
M2	OSMR	9180
M3	OTUD6B	51633
M1	OVGP1	5016
M2	OVOS	408186
M2	OVOS2	144203
M2	OXCT1	5019
M2	P2RX5	5026
M2	P2RX5-TA	1.01E+08
M2	P2RY12	64805
M3	P2RY13	53829
M2	P2RY14	9934
M2	P2RY8	286530
M1	PACSIN3	29763
M3	PAG1	55824
M1	PAIP2B	400961
M2	PALLD	23022
M1	PALM2	NA
M1	PALM3	342979
M3	PAM	5066
M1	PAN2	9924
M1	PANK1	53354
M2	PAPLN	89932
M2	PAPSS1	9061
M2	PAQR5	54852
M2	PAQR8	85315
M2	PARP12	64761
M2	PARP8	79668
M2	PARVG	64098
M2	PBK	55872
M1	PBLD	64081
M1	PCBD1	5092
M2	PCDH17	27253
M3	PCED1B	91523
M1	PCK1	5105
M2	PCNA	5111
M1	PCOLCE2	26577
M2	PCYOX1L	78991
M2	PDGFA	5154
M2	PDGFD	80310
M1	PDK4	5166

M2	PDP1	54704
M4	PDZK1IP1	10158
M2	PDZRN3	23024
M3	PEA15	8682
M2	PECAM1	5175
M2	PEG10	23089
M3	PFKFB3	5209
M2	PFKP	5214
M1	PFN2	5217
M2	PHF19	26147
M4	PHLDA1	22822
M2	PHLDA2	7262
M1	PHYHD1	254295
M3	PIGR	5284
M1	PIK3C2G	5288
M2	PIK3CG	5294
M2	PIK3R3	8503
M2	PILRA	29992
M2	PIM2	11040
M4	PIR	8544
M2	PIWIL4	143689
M2	PJA1	64219
M2	PKDCC	91461
M2	PKIB	5570
M1	PKLR	5313
M2	PKM	5315
M2	PLA2G2A	5320
M2	PLA2G4A	5321
M2	PLA2G7	7941
M2	PLAC8	51316
M2	PLAGL1	5325
M2	PLAT	5327
M2	PLAUR	5329
M2	PLBD1	79887
M2	PLCXD3	345557
M2	PLEK	5341
M2	PLEKHO1	51177
M2	PLEKHO2	80301
M1	PLG	5340
M1	PLIN1	5346
M2	PLIN3	10226
M3	PLK2	10769
M2	PLP2	5355
M2	PLSCR1	5359
M3	PLTP	5360
M2	PLXDC2	84898
M2	PLXNC1	10154
M4	PMEPA1	56937
M2	PNMA1	9240
M2	PNMA2	10687
M2	PNMAL1	NA
M2	PODXL	5420
M3	POLA1	5422
M2	POLE2	5427
M1	PON1	5444
M2	POU2AF1	5450
M2	PPA1	5464
M4	PPAP2C	NA

M2	PPAPDC1A	NA
M1	PPBP	5473
M2	PPDPF	79144
M2	PPIH	10465
M1	PPM1E	22843
M2	PPM1M	132160
M3	PPP1R12A	4659
M2	PPP1R14A	94274
M2	PPP1R16B	26051
M2	PPP1R18	170954
M1	PPP1R1A	5502
M1	PPP1R3C	5507
M3	PPP4R1	9989
M2	PPT1	5538
M2	PRC1	9055
M2	PREX1	57580
M2	PRF1	5551
M1	PRG4	10216
M3	PRKAR2B	5577
M2	PRKCD	5580
M2	PRKX	5613
M2	PRKY	5616
M2	PROCR	10544
M2	PROM1	8842
M1	PROX1	5629
M1	PROZ	8858
M4	PRR15L	79170
M2	PRSS23	11098
M2	PRTFDC1	56952
M2	PSMB10	5699
M2	PSMB8	5696
M2	PSMB9	5698
M2	PSMD14	10213
M2	PSME2	5721
M3	PSTPIP1	9051
M3	PSTPIP2	9050
M2	PTGDS	5730
M2	PTGER2	5732
M2	PTGER4	5734
M3	PTGFRN	5738
M2	PTPN22	26191
M2	PTPRC	5788
M2	PTPRCAP	5790
M2	PTTG1	9232
M2	PVRIG	79037
M2	PXDN	7837
M1	PXMP2	5827
M2	PYCARD	29108
M2	PYHIN1	149628
M1	PZP	5858
M2	QPCT	25797
M1	RAB11B-A	1.01E+08
M4	RAB25	57111
M3	RAB31	11031
M2	RAB32	10981
M2	RAB34	83871
M2	RAB38	23682
M2	RAB8B	51762

M2	RAC2	5880
M2	RACGAP1	29127
M2	RAD51AP1	10635
M3	RAI2	10742
M2	RALGDS	5900
M1	RAMP1	10267
M1	RANBP3L	202151
M1	RAPH1	65059
M2	RARRES1	5918
M2	RARRES3	NA
M2	RASGEF1A	221002
M3	RASGRP1	10125
M2	RASSF2	9770
M2	RASSF3	283349
M2	RBBP8	5932
M3	RBM3	5935
M1	RBP5	83758
M2	RCAN2	10231
M2	RCAN3	11123
M2	RCC2	55920
M1	RCL1	10171
M3	RCN2	5955
M3	RCSD1	92241
M2	RDH12	145226
M1	RDH16	8608
M3	RECQL	5965
M2	RELB	5971
M2	RERG	85004
M2	RFC4	5984
M2	RFX5	5993
M2	RGCC	28984
M3	RGL1	23179
M2	RGS1	5996
M2	RGS10	6001
M2	RGS18	64407
M3	RGS19	10287
M2	RGS2	5997
M2	RGS4	5999
M3	RHBDF2	79651
M2	RHNO1	83695
M2	RHOBTB1	9886
M2	RHOG	391
M2	RHOH	399
M2	RHOQ	23433
M2	RIPK2	8767
M2	RMI2	116028
M2	RNASE2	6036
M2	RNASE6	6039
M2	RNASEH2A	10535
M1	RND2	8153
M2	RND3	390
M2	RNF103-C	1.01E+08
M2	RNF135	84282
M3	RNF166	115992
M2	ROBO1	6091
M1	RORC	6097
M1	RP1-151F1	NA
M1	RP1-193H	NA

M2	RP1-93H1	NA
M4	RP11-11N	NA
M1	RP11-250E	NA
M1	RP11-327J	NA
M1	RP11-355E	NA
M1	RP11-384L	NA
M3	RP11-389(	NA
M2	RP11-38P2	NA
M2	RP11-401F	NA
M1	RP11-456H	NA
M1	RP11-458D	NA
M1	RP11-486(	NA
M2	RP11-488L	NA
M1	RP11-635N	NA
M2	RP11-642D	NA
M2	RP11-686D	NA
M1	RP11-740(	NA
M2	RP11-747H	NA
M1	RP11-863H	NA
M1	RP11-96D	NA
M1	RP3-406A	NA
M1	RP4-680D	NA
M1	RP4-791M	NA
M4	RP5-1154L	NA
M4	RPL22L1	200916
M2	RPL39L	116832
M3	RPS6KA1	6195
M2	RRAS	6237
M2	RRM2	6241
M3	RRM2B	50484
M2	RSAD2	91543
M2	RTN1	6252
M2	RTP4	64108
M2	RUNX1-IT	80215
M2	RUNX3	864
M1	RXRA	6256
M2	S100A11	6282
M2	S100A11P	729659
M4	S100A14	57402
M2	S100A4	6275
M2	S100A6	6277
M2	S100A8	6279
M2	S100A9	6280
M2	SACS	26278
M2	SAMD11	148398
M2	SAMD9	54809
M2	SAMD9L	219285
M2	SAMHD1	25939
M2	SAMSN1	64092
M2	SASH3	54440
M1	SAT2	112483
M1	SC5D	6309
M1	SCARA5	286133
M3	SCD	6319
M1	SCG5	6447
M2	SCGN	10590
M2	SCRN1	9805
M1	SDC2	6383

M4	SDCBP2	27111
M2	SECTM1	6398
M2	SEL1L3	23231
M1	SELENBP1	8991
M2	SELL	6402
M2	SELM	NA
M2	SEMA3C	10512
M2	SEMA4D	10507
M3	6-Sep	NA
M1	SERPINA5	5104
M2	SERPINB1	1992
M2	SERPINB8	5271
M2	SERPINB9	5272
M2	SERPINE2	5270
M1	SERPINF2	5345
M2	SERPINH1	871
M2	SFRP5	6425
M2	SFXN3	81855
M2	SGCB	6443
M3	SGK1	6446
M2	SGK223	NA
M2	SGOL2	NA
M2	SH3BGRL	6451
M3	SH3BGRL3	83442
M2	SHCBP1	79801
M1	SHD	56961
M1	SHF	90525
M2	SIDT1	54847
M1	SIGIRR	59307
M2	SIGLEC1	6614
M2	SIGLEC10	89790
M2	SIRPG	55423
M2	SKA2	348235
M1	SKAP1	8631
M1	SKIDA1	387640
M3	SLA	6503
M2	SLAMF6	114836
M2	SLAMF7	57823
M2	SLAMF8	56833
M2	SLC12A2	6558
M1	SLC13A5	284111
M2	SLC15A3	51296
M1	SLC16A10	117247
M2	SLC16A7	9194
M1	SLC17A2	10246
M3	SLC18B1	116843
M1	SLC19A2	10560
M1	SLC22A1	6580
M4	SLC22A15	55356
M1	SLC22A25	387601
M3	SLC25A33	84275
M2	SLC26A2	1836
M1	SLC27A5	10998
M4	SLC28A3	64078
M3	SLC29A3	55315
M2	SLC2A3	6515
M2	SLC2A6	11182
M2	SLC35G1	159371

M1	SLC37A4	2542
M2	SLC38A1	81539
M1	SLC38A4	55089
M2	SLC38A6	145389
M3	SLC39A10	57181
M1	SLC39A5	283375
M3	SLC44A2	57153
M3	SLC44A3	126969
M1	SLC45A3	85414
M2	SLC45A4	57210
M1	SLC47A1	55244
M2	SLC51A	200931
M4	SLC51B	123264
M1	SLC6A1	6529
M1	SLC6A13	6540
M1	SLC6A16	28968
M2	SLC7A6	9057
M2	SLC7A7	9056
M1	SLC9B2	133308
M1	SLCO1B1	10599
M2	SLFN11	91607
M4	SLFN12	55106
M4	SLFN13	146857
M2	SLFN5	162394
M2	SLIT2	9353
M1	SLITRK3	22865
M2	SMAD9	4093
M3	SMARCA5	8467
M3	SMC2	10592
M2	SMC4	10051
M1	SMIM1	388588
M1	SMIM14	201895
M1	SMIM2-AS	1.02E+08
M3	SMIM3	85027
M1	SMOC1	64093
M2	SMOC2	64094
M4	SMPX	23676
M3	SMS	6611
M2	SNAP25	6616
M2	SNHG12	85028
M2	SNN	8303
M2	SNORA16A	692073
M2	SNORA44	677825
M2	SNORA61	677838
M3	SNX7	51375
M1	SORD	6652
M1	SORL1	6653
M2	SORT1	6272
M1	SOWAHC	65124
M2	SOX4	6659
M2	SOX9	6662
M2	SP110	3431
M2	SP140	11262
M4	SPA17	53340
M3	SPAG1	6674
M3	SPARC	6678
M4	SPATS2	65244
M2	SPC25	57405

M3	SPDL1	54908
M3	SPIN4	139886
M4	SPINK1	6690
M4	SPINT2	10653
M2	SPON1	10418
M2	SPON2	10417
M2	SPP1	6696
M3	SPSB1	80176
M3	SPTSSA	171546
M1	SRD5A1	6715
M1	SRD5A2	6716
M2	SRGN	5552
M2	SRPX	8406
M4	SRXN1	140809
M1	SS18L1	26039
M2	SSPN	8082
M1	ST3GAL1	6482
M1	ST3GAL6	10402
M2	ST8SIA4	7903
M2	STAMBPL1	57559
M2	STARD3NL	83930
M2	STAT1	6772
M2	STEAP4	79689
M2	STIL	6491
M3	STK10	6793
M3	STK17A	9263
M2	STK17B	9262
M2	STK39	27347
M4	STMN2	11075
M2	STX11	8676
M2	SUCO	51430
M3	SULF2	55959
M1	SULT1E1	6783
M2	SUSD1	64420
M2	SWAP70	23075
M2	SYK	6850
M1	SYPL2	284612
M2	SYS1-DBN	767557
M2	SYT13	57586
M1	SYT17	51760
M2	SYTL1	84958
M2	TACC3	10460
M2	TAGAP	117289
M2	TAGLN	6876
M2	TAGLN2	8407
M2	TAP1	6890
M2	TAP2	6891
M3	TARP	445347
M1	TAT	6898
M2	TAX1BP3	30851
M2	TBC1D10C	374403
M1	TBC1D2	55357
M2	TC2N	123036
M1	TCEA3	6920
M2	TCEAL3	85012
M3	TCEAL7	56849
M2	TCEAL8	90843
M1	TCEB3-AS:	NA

M2	TCF19	6941
M2	TCF4	6925
M2	TCHH	7062
M2	TCIRG1	10312
M2	TCL1A	8115
M2	TCTEX1D2	255758
M2	TDGF1	6997
M2	TDGF1P3	6998
M1	TDRD6	221400
M2	TDRD7	23424
M1	TENM1	10178
M1	TENM2	57451
M2	TES	26136
M4	TESC	54997
M2	TFEC	22797
M3	TFPI2	7980
M1	TFR2	7036
M3	TGFB1I1	7041
M2	TGFBI	7045
M2	THBD	7056
M2	THBS1	7057
M2	THBS2	7058
M2	THEMIS	387357
M2	THEMIS2	9473
M1	THOP1	7064
M1	THRB-IT1	1.01E+08
M1	THRSP	7069
M4	THSD7A	221981
M4	THY1	7070
M2	TIGIT	201633
M3	TIMD4	91937
M2	TIMP1	7076
M3	TIMP2	7077
M2	TK1	7083
M2	TLCD1	116238
M3	TLR1	7096
M2	TLR2	7097
M3	TLR4	7099
M3	TLR5	7100
M2	TLR7	51284
M2	TLR8	51311
M3	TM6SF1	53346
M1	TM6SF2	53345
M4	TMC4	147798
M2	TMC6	11322
M2	TMEM100	55273
M1	TMEM120/	83862
M4	TMEM125	128218
M2	TMEM154	201799
M3	TMEM164	84187
M2	TMEM173	NA
M4	TMEM200/	114801
M1	TMEM220	388335
M2	TMEM243	79161
M4	TMEM255/	55026
M1	TMEM256	254863
M1	TMEM44-/	1.01E+08
M2	TMEM45B	120224

M2	TMEM50A	23585
M2	TMEM51	55092
M1	TMEM52	339456
M4	TMEM55A	NA
M1	TMEM56	NA
M3	TMEM87B	84910
M4	TMPRSS3	64699
M2	TMSB10	9168
M3	TMSB4X	7114
M2	TNF	7124
M2	TNFAIP3	7128
M2	TNFAIP8	25816
M2	TNFAIP8L2	79626
M2	TNFRSF11A	8792
M3	TNFRSF11B	4982
M2	TNFRSF12A	51330
M2	TNFRSF17	608
M3	TNFRSF1B	7133
M2	TNFRSF21	27242
M2	TNFSF13B	10673
M2	TOMM40L	84134
M2	TOP2A	7153
M2	TOX	9760
M3	TOX2	84969
M3	TOX3	27324
M3	TP53BP2	7159
M2	TP53I3	9540
M2	TPM1	7168
M1	TPPP2	122664
M2	TPX2	22974
M2	TRAC	28755
M2	TRAF3IP3	80342
M2	TRAF5	7188
M2	TRAJ17	28738
M2	TRAPPC2P	NA
M2	TRAT1	50852
M2	TRBC1	28639
M2	TRDV2	28517
M2	TREM2	54209
M2	TRG-AS1	1.01E+08
M3	TRGC2	6967
M3	TRGV9	6983
M2	TRIM21	6737
M3	TRIM22	10346
M4	TRIM31	11074
M1	TRIM55	84675
M2	TRIM6	117854
M2	TRIM69	140691
M2	TRIP13	9319
M4	TRNP1	388610
M1	TSLP	85480
M3	TSPAN8	7103
M2	TSPO	706
M2	TSPYL5	85453
M1	TTC36	143941
M2	TTK	7272
M1	TTPA	7274
M2	TUBA1A	7846

M2	TUBA1B	10376
M2	TUBA1C	84790
M2	TUBB6	84617
M3	TUBG1	7283
M2	TUSC3	7991
M2	TWSG1	57045
M2	TYMP	1890
M2	TYMS	7298
M2	TYMSOS	494514
M2	TYROBP	7305
M2	UAP1L1	91373
M2	UBASH3A	53347
M3	UBD	10537
M2	UBE2A	7319
M2	UBE2C	11065
M2	UBE2L6	9246
M3	UBE2Q2	92912
M2	UBE2S	27338
M2	UBE2T	29089
M3	UBLCP1	134510
M2	UCP2	7351
M3	UGCG	7357
M3	UGT2A3	79799
M2	UHRF1	29128
M1	UPB1	51733
M2	UPP2	151531
M2	UTS2	10911
M2	UXS1	80146
M2	VAMP5	10791
M2	VAV1	7409
M4	VCAN	1462
M3	VEGFC	7424
M1	VIL1	7429
M2	VIM	7431
M2	VMO1	284013
M2	VMP1	81671
M2	VNN2	8875
M2	VOPP1	81552
M3	VRK1	7443
M2	VSIG10L	147645
M2	VSIG4	11326
M1	VSNL1	7447
M4	VTCN1	79679
M2	VWA5A	4013
M2	WARS	NA
M2	WBP5	NA
M2	WDR54	84058
M3	WDYHV1	55093
M2	WFDC1	58189
M2	WIPF1	7456
M3	WIPI1	55062
M2	WISP1	NA
M3	WLS	79971
M2	WSB2	55884
M3	WWC3	55841
M2	XAF1	54739
M2	XCL1	6375
M2	XCL2	6846

M3	YAP1	10413
M3	YWHAB	7529
M2	YWHAZ	7534
M2	ZAP70	7535
M1	ZBTB16	7704
M2	ZC3H12D	340152
M1	ZCCHC6	NA
M1	ZDHHC11	79844
M1	ZDHHC11I	653082
M2	ZEB2	9839
M2	ZFP3	124961
M3	ZFPM2	23414
M1	ZG16	653808
M2	ZG16B	124220
M1	ZGPAT	84619
M1	ZKSCAN1	7586
M2	ZMAT3	64393
M2	ZNF101	94039
M2	ZNF124	7678
M2	ZNF14	7561
M4	ZNF165	7718
M3	ZNF200	7752
M2	ZNF267	10308
M2	ZNF367	195828
M1	ZNF385B	151126
M1	ZNF395	55893
M2	ZNF430	80264
M2	ZNF439	90594
M3	ZNF468	90333
M1	ZNF511	118472
M2	ZNF532	55205
M2	ZNF83	55769
M2	ZNFX1	57169
M2	ZUFSP	NA
M2	ZWILCH	55055
M2	ZWINT	11130
